# RNA helicase DHX29 controls the translation of transcription factors involved in germinal center response and plasma cell differentiation in mice

**DOI:** 10.1038/s44318-026-00805-0

**Published:** 2026-05-26

**Authors:** Jiayi Zhao, Xiaoyu He, Peicheng Hong, Lianghua Lin, Ying Du, Pengda Chen, Lixiao Zhang, Jiale Leng, Lihui Ma, Jun Xie, Xinyong Lin, Abidan Adilijiang, Jiazhen Wang, Yazhen Hong, Zhengtao Xiao, Wen-Hsien Liu, Changchun Xiao

**Affiliations:** 1 State Key Laboratory of Cellular Stress Biology, School of Life Sciences, Faculty of Medicine and Life Sciences, Xiamen University, Xiamen, China; 2 Center for Precision Cancer Medicine & Translational Research, National Clinical Research Center for Cancer, Key Laboratory of Cancer Prevention and Therapy, Tianjin’s Clinical Research Center for Cancer, Tianjin Medical University Cancer Institute & Hospital, Tianjin, China; 3 Department of Clinical Laboratory, Tianjin Central Hospital of Obstetrics and Gynecology, Nankai University Affiliated Maternity Hospital, Tianjin, China; 4 Institute of Molecular and Translational Medicine (IMTM), Department of Biochemistry and Molecular Biology, Xi’an Jiaotong University Health Science Center, Xi’an, China; 5 Department of Immunology and Microbiology, The Scripps Research Institute, La Jolla, CA USA

**Keywords:** Immunology, Translation & Protein Quality

## Abstract

The roles of translational control in the immune system are incompletely understood. Using a CRISPR/Cas9-mediated functional screen of RNA helicases in an in vitro system of plasma cell differentiation, we identify in this study DHX29 as a critical regulator of this process. Mice with B-cell-specific deletion of *Dhx29* exhibit severely impaired germinal center B-cell formation, plasma cell differentiation, and antibody production. Mechanistically, DHX29 promotes translation of *Tcf3* and *Tle3* mRNAs via binding to their 5’ untranslated regions (UTRs). In the absence of DHX29, B cells exhibit normal proliferation but fail to undergo class switch to IgG1 and differentiation into plasma cells, resulting in impaired antibody production. Ectopic expression of TCF3 and TLE3 largely restores plasma cell differentiation of *Dhx29*-deficient B cells. Our study provides insights into the functional importance of translational control in the immune system by unraveling critical roles of the RNA helicase DHX29 in the translation of key transcription factors controlling germinal center response and plasma cell differentiation.

## Synopsis

Translational control is crucial for the regulation of gene expression. This study identifies the RNA helicase DHX29 in mice as a key regulator of germinal center response and plasma cell differentiation by promoting translation of the transcriptional factors TCF3 and TLE3.

- A CRISPR screen identifies DHX29 as a regulator of plasma cell differentiation.
- B-cell-specific *Dhx29* deletion impairs germinal center formation, plasma cell differentiation, and antibody production in vivo.
- DHX29 binds the 5’ UTRs of *Tcf3* and *Tle3* mRNAs to promote their translation.
- Ectopic expression of TCF3 and TLE3 substantially rescue plasma cell differentiation of *Dhx29*-deficient B-cells, highlighting TLE3 as a previously unrecognized regulator of this process.

## Abstract (web summary)

DHX29 binds the 5’ UTRs of *Tcf3* and *Tle3* mRNAs to promote their translation.

## Graphical abstract

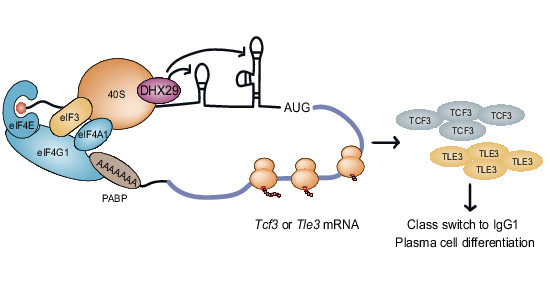

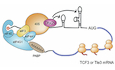

## Introduction

Regulation of translation is widely used to modulate gene expression. There are general modes of translational regulation—global control, in which the translation of most mRNAs in the cell is regulated; and mRNA-specific control, whereby the translation of a defined group of mRNAs is modulated without affecting general protein biosynthesis or the translational status of the cellular transcriptome as a whole. Global control mainly occurs by modulating translation machinery and translation initiation factors, whereas mRNA-specific translational control is driven by regulatory protein complexes recognizing *cis*-elements present in the 5’ and 3’ untranslated regions (UTRs) of the mRNA (Gebauer and Hentze, 2004).

The translation process can be divided into three phases: initiation, elongation, and termination. Translation initiation involves the positioning of an elongation-competent 80S ribosome at the start codon (AUG). The 40S small ribosomal subunit, together with other factors, forms a 43S pre-initiation complex that binds to the 5’ end of mRNA. The 43S complex contains eukaryotic initiation factors (eIFs) 3, 1, 1A, 5, and a ternary complex that consists of the methionine-loaded initiator tRNA that recognizes the AUG start codon during initiation and eIF2 that is coupled to GTP. The binding of the 43S complex to the mRNA involves bridging interactions between eIF3 and the eIF4F protein complex, which associates with the 5’ cap structure of the mRNA. The eIF4F complex contains three proteins: eIF4E, which binds to the 5’ cap structure; eIF4A, an RNA helicase that unwinds secondary structures in the 5’UTR so that the 43S pre-initiation complex (PIC) can bind and scan the mRNA; and eIF4G, which functions as a scaffold protein by interacting with eIF4E, eIF4A, and eIF3. eIF4G also interacts with the poly(A)-binding protein (PABP). The simultaneous interactions of eIF4G with eIF4E and PABP circularize the mRNA, bringing the 3’UTR to close proximity of the 5’ end of the mRNA. This provides a conceptual framework in which 3’UTR-binding factors can participate in the regulation of translation initiation (Gebauer and Hentze, 2004).

The 43S pre-initiation complex scans along the 5’UTR until it reaches and identifies the start codon through the formation of base pairs between the initiator tRNA and the start codon. This is followed by the hydrolysis of eIF2-bound GTP, the joining of the 60S ribosomal subunit, the release of most of the initiation factors from the 40S small ribosomal subunit, and the formation of an elongation-competent 80S ribosome ready for polypeptide synthesis (Gebauer and Hentze, 2004).

Most mechanisms of translational control affect the rate of initiation. Translation initiation is controlled by the complex and dynamic interplay between structures of the mRNA and trans-factors that include ribosome, translation initiation factors, and RNA-binding proteins (RBPs). The cellular levels of available ribosomes, initiation factors, RBPs, and Met-tRNAi all influence initiation rates. The trans-factors can also be modified by phosphorylation or other means, which can affect the activities of those factors and the rate of translation initiation (Gebauer and Hentze, 2004).

RNA helicases are critical trans-factors controlling translation initiation. They use ATP to bind, unwind, and disrupt RNA structures and RNA-protein complexes. It has long been known that secondary structures within 5’UTRs inhibit translation and, when located near the 5’ cap, negatively impact the interaction of eIF4F with mRNA. The complexity of structures within 5’UTR links translation initiation of mRNA to RNA helicase activity. The greater the complexity of 5’UTR structures, the more dependent of mRNA appears to be on RNA helicases (Gebauer and Hentze, 2004; Shen and Pelletier, 2020).

We are interested in the roles of RNA helicases in translational control in the immune system, with a focus on the humoral immune response. The humoral immune response is characterized by the generation of high-affinity antibody-secreting plasma cells. The production of high-affinity antibodies is not only critical for eliminating pathogens during infection, but also confers long-term protection against the same microorganisms. Generation of those plasma cells occurs within the unique microenvironment of germinal centers in secondary lymphoid tissues. After antigen binding and activation via B-cell receptor (BCR), B cells migrate to and establish germinal centers within B-cell follicles, where they receive help from T follicular helper (Tfh) cells, undergo somatic hypermutation (SHM), class-switch recombination (CSR), and affinity maturation (Mesin et al, 2016; Victora and Nussenzweig, 2012). Germinal center B cells (GCBs) with high antigen-binding affinity then differentiate into antibody-secreting plasma cells or memory B cells (Suan et al, 2017). The differentiation of germinal center B cells into plasma cells requires the coordinated regulation of hundreds of genes, which are controlled by a few key transcription factors. B lymphocyte-induced maturation protein 1 (BLIMP1, encoded by the *Prdm1* gene) has long been recognized as the master regulator of plasma cell differentiation. *Prdm1*^fl/fl^*CD19*^Cre/+^ mice, which delete *Prdm1* specifically in B cells, cannot form plasma cells or secrete immunoglobulins normally after antigen immunization, while ectopic expression of BLIMP1 drives plasma cell differentiation (Shapiro-Shelef et al, 2003; Turner et al, 1994). BLIMP1 functions as a transcriptional repressor and inhibits some critical transcription factors that control germinal center B-cell identity, such as PAX5 and BCL6 (Kallies et al, 2007; Lin et al, 2002; Shaffer et al, 2002). It also activates IRF4 expression, immunoglobulin gene transcription, and expression of many components of the unfolded protein response (UPR), including XBP1 (Minnich et al, 2016; Tellier et al, 2016). IRF4 is essential for the survival of plasma cells (Tellier et al, 2016), while both BLIMP1 and XBP1 play critical roles in inducing the expression of genes required for endoplasmic reticulum (ER) expansion and function (Lee et al, 2003; Shaffer et al, 2004). As plasma cells are the cellular factories specialized in producing antibodies, which must be correctly folded and modified within the ER, plasma cells are critically dependent on ER stress responses such as UPR to maintain homeostasis. It has been reported that many genes involved in UPR are directly regulated by BLIMP1, IRF4, or XBP1 (Lee et al, 2003; Low et al, 2019; Shaffer et al, 2004; Tellier et al, 2016).

Recent studies have begun to reveal the roles of RNA helicases in humoral immune responses. For example, DDX1 plays a critical role in CSR by binding to G-quadruplex structures within intronic switch transcripts and converting them into S-region R-loops, thereby facilitating the recruitment of the cytidine deaminase AID to S-regions to promote CSR (Ribeiro de Almeida et al, 2018). Another member of the DEAD-box family, DDX3X, is essential for B-cell development at the pro- and pre-B-cell stages and is required for the formation and expansion of germinal center B cells (Lacroix et al, 2022). Interestingly, in the context of MYC-driven lymphomagenesis, loss of DDX3X prevents lymphoma development in female but not male mice, where the Y-linked paralog DDX3Y compensates for DDX3X deficiency and supports lymphoma progression (Gong et al, 2021; Lacroix et al, 2022). Recent work from our group and others has shown that eIF4A1 is required for early B-cell development and germinal center responses by promoting global protein synthesis (Du et al, 2025; Screen et al, 2024). Despite these advances, the functions of many other RNA helicases in the immune system, under both physiological and pathological conditions, remain largely unexplored.

We established a CRISPR/Cas9-mediated functional screening platform to identify novel regulators of B-cell proliferation and plasma cell differentiation. In a recent study, we reported that *Dhx33*, an RNA helicase upregulated upon B-cell activation, plays an essential role in B-cell growth and proliferation (He et al, 2023). In the present study, we expanded this approach to systematically screen all known RNA helicases and identified *Dhx29* as a critical regulator of plasma cell differentiation. Mice with *Dhx29* specifically deleted in activated B cells exhibited profound defects in germinal center formation, plasma cell differentiation, and antibody production. Mechanistically, DHX29 promotes the translation of *Tcf3* and *Tle3* by binding directly to their mRNAs. These two transcriptional regulators, in turn, bind to the promoter region of *Prdm1*, facilitating its transcription. In the absence of *Dhx29*, B cells maintain normal proliferation but fail to undergo class switch recombination to IgG1 and differentiation into plasma cells, resulting in impaired humoral responses. Notably, ectopic expression of TCF3 and TLE3 largely rescues plasma cell differentiation in *Dhx29*-deficient B cells. Together, these findings identify *Dhx29* as a key translational regulator of transcription factors governing germinal center responses and plasma cell differentiation, underscoring the critical role of translational control in humoral immunity.

## Results

### CRISPR/Cas9 screen identified DHX29 as a critical regulator of plasma cell differentiation

**Figure 1:**
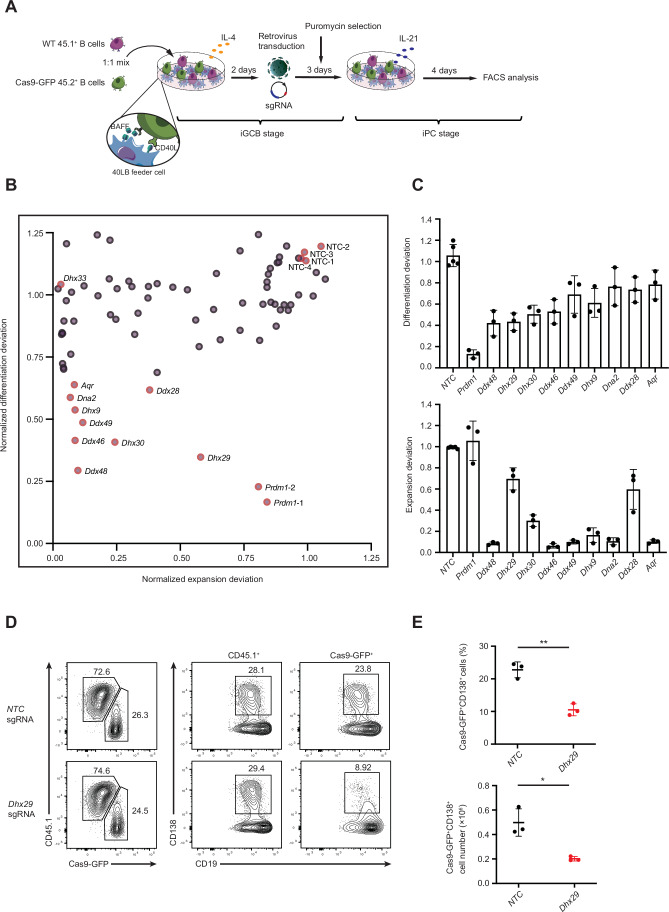
CRISPR/Cas9 screening identifies DHX29 as a critical regulator of plasma cell differentiation. (**A**) Experimental outline of CRISPR/Cas9 screening in the iGCB system. Naïve B cells from *Rosa26*^Cas9-GFP^ (CD45.2^+^) and CD45.1^+^ wild-type mice were mixed in a 1:1 ratio, cultured in the iGCB system, transduced with retroviruses encoding sgRNA (BFP^+^) at day 2.5, and treated with puromycin at day 3. iGCB cells at day 5 were replated on fresh 40LB cells with IL-21 to induce iPC differentiation for another 4 days and analyzed by flow cytometry. (**B**) Dot plot representation of CRISPR/Cas9 screening results. *X* axis shows normalized expansion deviation, while *Y* axis shows normalized differentiation deviation. Each symbol represents an individual sgRNA. See Fig. EV1B for the calculation of expansion and differentiation deviation. (**C**) Differentiation (upper) and expansion deviation (lower) caused by sgRNAs targeting indicated RNA helicases. Each symbol represents an individual sgRNA. (**D**, **E**) CRISPR/Cas9 screening results of sgRNA targeting *Dhx29*. Representative FACS plots (**D**) and percentage (upper) and number (lower) of Cas9-GFP^+^CD138^+^ iPCs at day 4 of iPC culture (**E**). Data were pooled from three independent experiments (percentage of Cas9-GFP^+^CD138^+^ iPCs: *P* = 0.0022; number of Cas9-GFP^+^CD138^+^ iPCs: *P*  =  0.0107). Small horizontal lines indicate the mean ( ± SD). Statistical significance was determined by unpaired, two-tailed Student’s *t* test in (**E**). *P  <  0.05, **P  <  0.01. Source data are available online for this figure.

We compiled a list of 72 mouse RNA helicases (excluding Dicer 1) from the HUGO Gene Nomenclature Committee (HGNC) website and a previous study (Table EV1) (Umate et al, 2011). SgRNAs targeting these RNA helicases (3 sgRNAs per gene) and *Prdm1* (positive control), as well as non-targeting control (NTC, negative control) sgRNAs, were cloned into a retroviral vector encoding the puromycin resistance gene as a selection marker. As shown in Fig. 1A, naïve B cells from *Rosa26*^Cas9-GFP^ (CD45.2^+^) and wild-type (CD45.1^+^) mice were mixed in a 1:1 ratio, co-cultured with 40LB cells and IL-4 for iGCB differentiation (Nojima et al, 2011), and transduced by retroviruses encoding puromycin-resistant gene and individual sgRNAs. Transduced B cells were selected by puromycin and replated on 40LB cells in the presence of IL-21 to induce plasma cell differentiation (Nojima et al, 2011). The percentage of Cas9-GFP^+^ B cells among total B cells, as well as the percentage of CD138^+^ cells among Cas9-GFP^+^ (CD45.2^+^) and wild-type (CD45.1^+^) B cells, was analyzed on day 4 of the iPC culture stage.

**Figure EV1:**
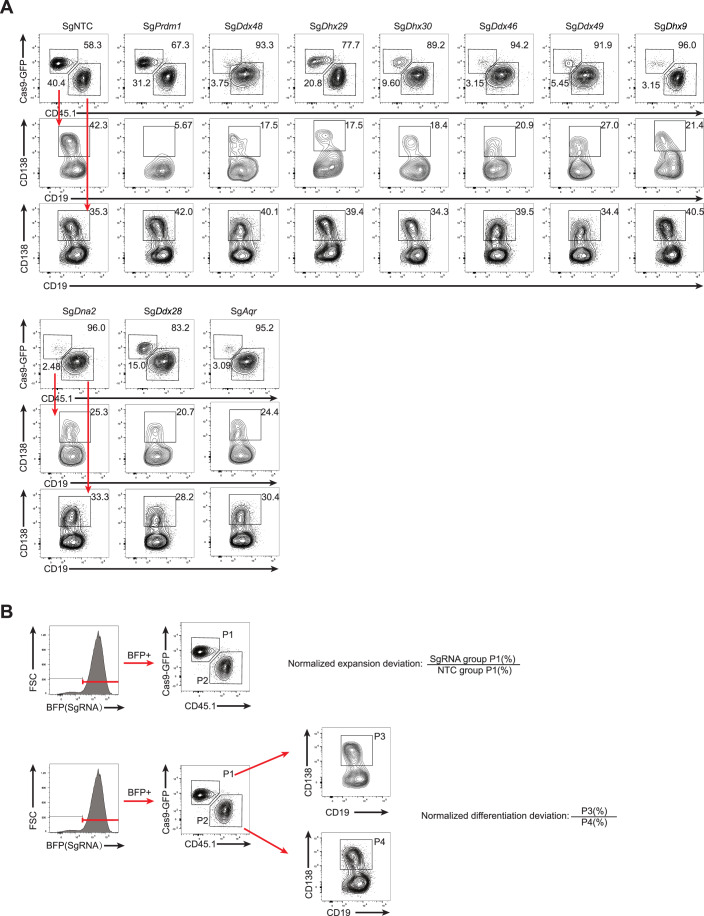
Validation of positive hits from CRISPR/Cas9 screening. (**A**) Naïve B cells from Cas9-GFP (CD45.2^+^) and CD45.1^+^ wild-type mice were mixed in a 1:1 ratio, cultured in the iGCB system, transduced with retroviruses encoding indicated sgRNAs, induced for iPC differentiation for 4 days, and analyzed by flow cytometry for the percentage of CD45.1^+^ and Cas9-GFP^+^ cells, as well as the percentage of iPCs (CD19^+^CD138^+^). (**B**) FACS gating strategy and calculation of normalized expansion deviation and differentiation deviation.

While deletion of many RNA helicases severely impaired B-cell expansion, deletion of nine of them compromised plasma cell differentiation (Figs. 1B,C and EV1). Among those nine RNA helicases, DHX29 deficiency led to a strong inhibition of plasma cell differentiation, accompanied by a mild effect on B-cell expansion (Figs. 1B–E and EV1), suggesting a critical role of DHX29 in plasma cell differentiation. We therefore focused on DHX29 in this study.

### Impaired germinal center response, plasma cell differentiation, and antibody production in the absence of DHX29

**Figure 2:**
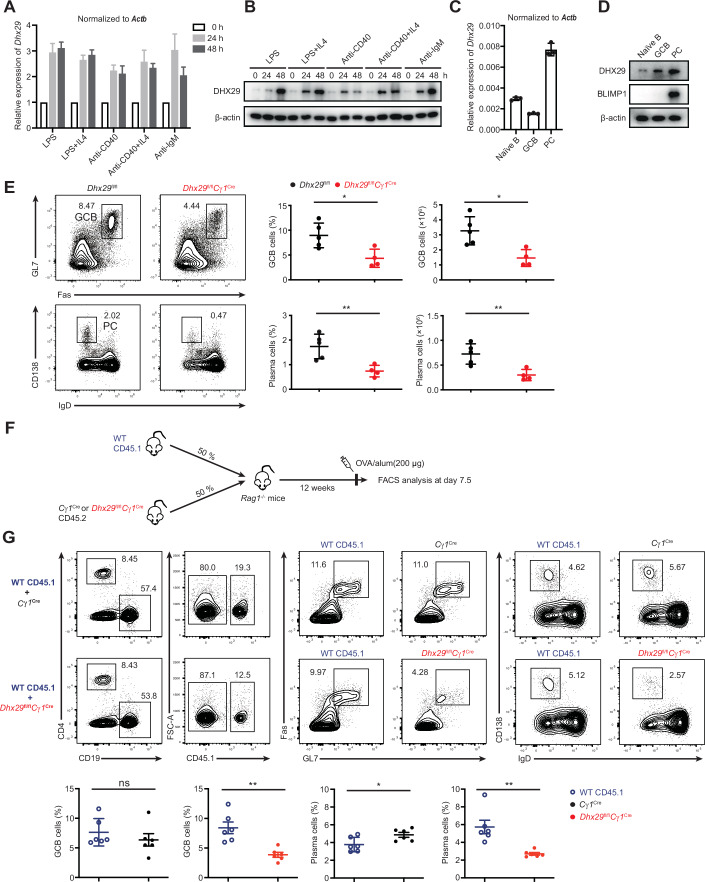
DHX29 plays a critical role in T-dependent immune responses. (**A**, **B**) RT‒qPCR (**A**) and immunoblot (**B**) analysis of DHX29 mRNA and protein expression in B cells activated with various stimuli for the indicated amounts of time. (**C**, **D**) RT‒qPCR(**C**) and immunoblot (**D**) analysis of DHX29 mRNA and protein expression in naïve B, GCB, and plasma cells (PCs) sorted from wild-type mice at day 7.5 post immunization with OVA/alum/LPS. Each symbol represents an individual mouse (*n* = 3 per group). (**E**) Flow cytometry analysis of GCB (GL7^+^Fas^+^, upper) and plasma cells (CD138^+^IgD^−^, lower) in the spleen of *Dhx29*^fl/fl^ and *Dhx29*^fl/fl^*Cγ1*^Cre^ mice at day 7.5 post immunization with OVA/alum (i.p). Dot plots summarizing the percentage and number of GCB (upper) and plasma cells (lower). Each symbol represents an individual mouse (*n* = 4–5 per group). GCB cells (%): *P* = 0.0179; GCB cells ( × 10^6^): *P* = 0.0119; Plasma cells (%): *P* = 0.0064; Plasma cells ( × 10^6^): *P* = 0.0069. (**F**) Experimental outline for the generation of mixed bone marrow chimeras. *Rag1*^−/−^ recipient mice were reconstituted with a 50:50 mixture of wild-type (CD45.1^+^) and *Cγ1*^Cre^ or *Dhx29*^fl/fl^*Cγ1*^Cre^ (CD45.2^+^) bone marrow cells. Twelve weeks after reconstitution, recipient mice were immunized with OVA/alum and analyzed by flow cytometry at day 7.5 post immunization. i.v., intravenous injection; i.p., intraperitoneal injection. (**G**) Flow cytometry analysis of splenic GCB and plasma cells from *Rag1*^−/−^ recipients at day 7.5 post immunization with OVA/alum (i.p.) (upper). Dot plots summarizing the percentages of GCB and plasma cells. Each symbol represents an individual mouse (*n* = 6 per group). *P* value from left to right: 0.0017, 0.0300, 0.0029. Small horizontal lines indicate the mean ( ± SD). Statistical significance was determined by unpaired, two-tailed Student’s *t* test in (**E**, **G**). *P  <  0.05, **P  <  0.01. Source data are available online for this figure.

**Figure 3:**
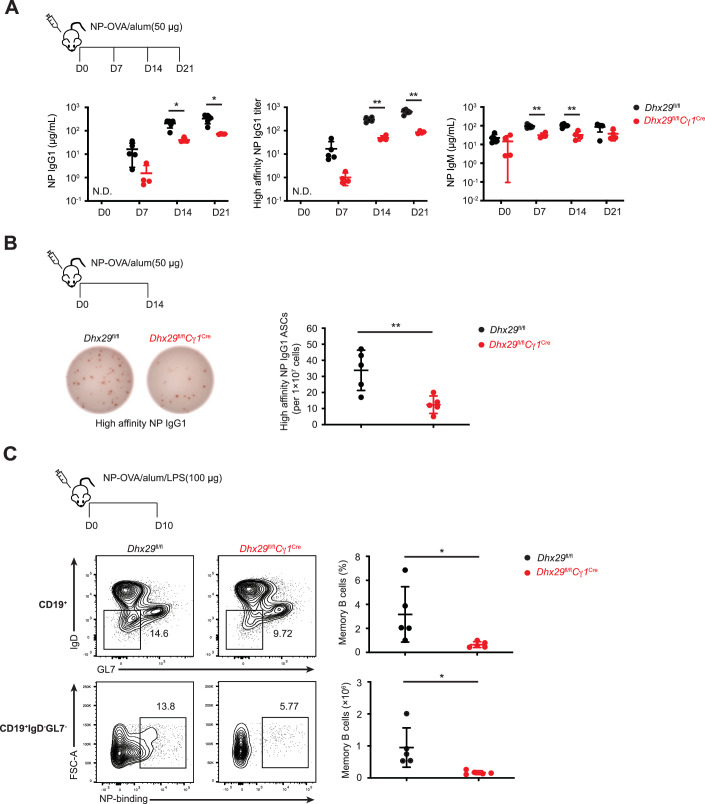
B-cell expression of DHX29 is required for antigen-specific antibody response. (**A**) Concentration of NP-specific IgM, IgG1, and high-affinity NP-IgG1 in the serum of *Dhx29*^fl/fl^ and *Dhx29*^fl/fl^*Cγ1*^Cre^ mice was determined by ELISA at day 0, 7, 14, and 21 post immunization with 50 μg NP-OVA in alum (i.p). Each symbol represents an individual mouse (*n *= 4–5 per group). NP-specific IgG1 14 day: *P* = 0.0227, 21 day: *P* = 0.0377; high-affinity NP-IgG1 14 day: *P* = 0.0027, 21 day: *P* = 0.0030; NP-specific IgM 7 day: *P* = 0.0070, 14 day: *P* = 0.0036. (**B**) NP-specific antibody-secreting cells (ASCs) in the spleen were measured by ELISpot assay at day 14 post immunization with NP-OVA/Alum (i.p.). Each symbol represents an individual mouse (*n* = 5 per group). *P* = 0.0080. (**C**) Flow cytometry analysis of NP-specific memory B cells (CD19^+^IgD^−^GL7^−^) in the spleen of *Dhx29*^fl/fl^ and *Dhx29*^fl/fl^*Cγ1*^Cre^ mice at 10 days post immunization with NP-OVA/alum/LPS (i.p). Dot plots summarizing the percentage (upper) and number (lower) of memory B cells. Memory B cells (%): *P* = 0.0415; Memory B cells ( × 10^6^): *P* = 0.0218. Each symbol represents an individual mouse. (*n* = 5 per group). Small horizontal lines indicate the mean ( ± SD). Statistical significance was determined by two-way ANOVA in (**A**); unpaired, two-tailed Student’s *t* test in (**B**, **C**). *P  <  0.05, **P  <  0.01. N.D. not detected. Source data are available online for this figure.

We first examined DHX29 mRNA and protein expression in B cells activated by various stimuli (Fig. 2A,B). Notably, robust expression of DHX29 was induced in activated B cells, suggesting a potential role of DHX29 in this process. DHX29 was expressed at much higher levels in plasma cells than naïve B and GCB cells sorted from splenocytes of immunized mice (Fig. 2C,D). To investigate the biological function of DHX29 in B cells, we generated a loxP-site flanked allele of DHX29 (Appendix Fig. S1A) and crossed the mutant mice with *Mb1*^Cre^ and *CD19*^Cre^ mice in which expression of the Cre recombinase is under the control of the *CD79a* and *CD19* loci, respectively (Hobeika et al, 2006; Rickert et al, 1997). As shown in Appendix Figs. S1B–H and S2, B cells in the spleen and peripheral lymph nodes were significantly reduced in *Dhx29*^fl/fl^*Mb1*^Cre^ and *Dhx29*^fl/fl^*CD19*^Cre^ mice, which prevented the study of DHX29 functions during B-cell immune responses. We therefore bred *Dhx29*^fl/fl^ with *Cγ1*^Cre^ mice in which the expression of Cre recombinase is under control of the *Cγ1* locus and turned on in activated B cells (Casola et al, 2006). The proportions and numbers of various B-cell subsets in the bone marrow, spleen, and peripheral lymph nodes of *Dhx29*^fl/fl^*Cγ1*^Cre^ mice were similar to those of *Dhx29*^fl/fl^ control mice (Appendix Fig. S3). We immunized those mice with ovalbumin (OVA) precipitated in alum (OVA/alum), a T cell-dependent antigen. At day 7.5 after immunization, the percentage and number of GCB and plasma cells were drastically decreased in *Dhx29*^fl/fl^*Cγ1*^Cre^ mice (Fig. 2E). Immunohistochemistry analysis showed a significant reduction in the size of germinal centers in the spleen of immunized *Dhx29*^fl/fl^*Cγ1*^Cre^ mice (Appendix Fig. S4). To investigate the cell-autonomous role of DHX29 in germinal center formation and plasma cell differentiation, we generated mixed bone marrow chimeras by reconstituting lethally irradiated *Rag1*^*−/−*^ mice with wild-type (CD45.1^*+*^) and *Cγ1*^Cre^ or *Dhx29*^fl/fl^*Cγ1*^Cre^ (CD45.2^+^) bone marrow cells at a 50:50 ratio. Twelve weeks after reconstitution, those chimeras were immunized with OVA/alum and analyzed by flow cytometry 7.5 days later (Fig. 2F). The frequencies of GCB and plasma cells derived from *Dhx29*^fl/fl^*Cγ1*^Cre^ (CD45.2^+^) B cells were much lower than those from WT (CD45.1^+^) and *Cγ1*^Cre^ (CD45.2^+^) B cells (Fig. 2G), demonstrating a cell-intrinsic role of DHX29 in GCB formation and plasma cell differentiation. We also measured NP-specific antibody responses in *Dhx29*^fl/fl^*Cγ1*^Cre^ mice immunized with 4-hydroxy-3-nitrophenyl hapten conjugated to ovalbumin (NP-OVA) precipitated in alum (NP-OVA/alum). Both NP-specific IgM and IgG1 antibodies were markedly decreased in *Dhx29*^fl/fl^*Cγ1*^Cre^ mice (Fig. 3A). Consistently, the number of NP-specific IgG1 antibody-secreting cells (ASC) was drastically reduced in those immunized *Dhx29*^fl/fl^*Cγ1*^Cre^ mice (Fig. 3B). To further investigate whether DHX29 is required for the generation of GC-dependent memory B cells, we immunized *Dhx29*^fl/fl^ and *Dhx29*^fl/fl^ *Cγ1*^Cre^ mice with NP-OVA/alum/LPS and analyzed 10 days later. The abundance of NP-specific memory B cells was significantly decreased in the spleen of *Dhx29*^fl/fl^*Cγ1*^Cre^ mice (Fig. 3C). Taken together, these results demonstrate that DHX29 is required for germinal center response, plasma cell differentiation, and antibody production.

### DHX29 promotes plasma cell differentiation and class switch to IgG1

**Figure 4:**
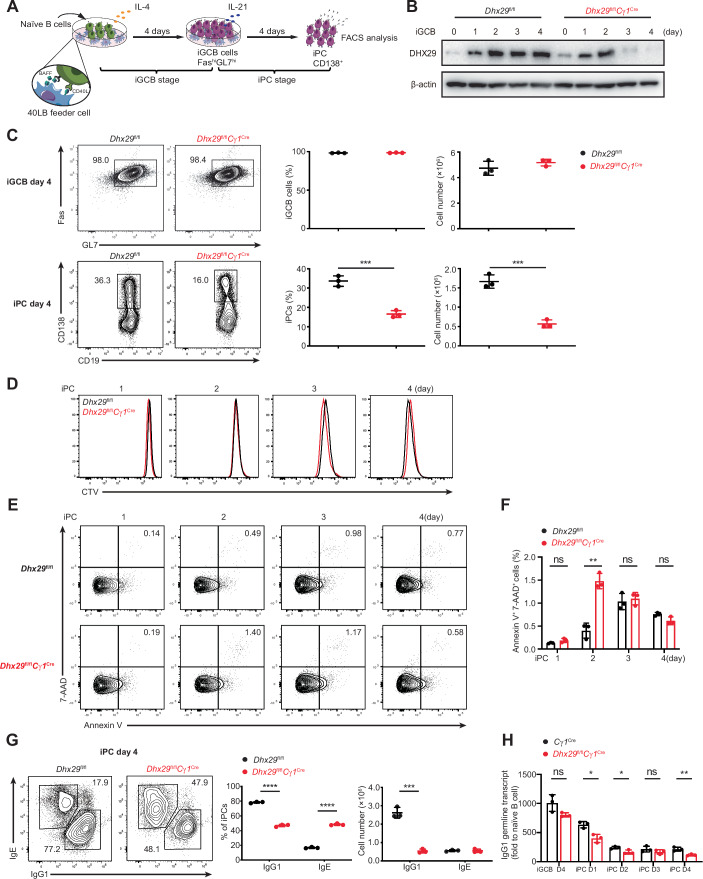
DHX29 promotes plasma cell differentiation and class switch to IgG1. (**A**) Schematic representation of the iGCB in vitro culture system. (**B**) Immunoblot analysis of DHX29 expression in *Dhx29*^fl/fl^ and *Dhx29*^fl/fl^*Cγ1*^Cre^ B cells during iGCB culture. (**C**) Flow cytometry analysis of iGCB cells (CD19^+^Fas^hi^GL7^hi^) at day 4 and iPCs (CD19^+^CD138^+^) at day 8 of culture. Dot plots summarizing the percentage (left) and number (right) of *Dhx29*^fl/fl^ and *Dhx29*^fl/fl^*Cγ1*^Cre^ iGCB cells and iPCs at the indicated time points of culture. Data from three independent biological replicates (iPC cells (%): *P* = 0.0008; iPC cells ( × 10^6^): *P* = 0.0007). (**D**) Proliferation of *Dhx29*^fl/fl^ and *Dhx29*^fl/fl^*Cγ1*^Cre^ B cells in iPC culture was measured by CTV dilution. (**E**, **F**) Flow cytometry analysis of Annexin V^+^7-AAD^+^ cells among *Dhx29*^fl/fl^ and *Dhx29*^fl/fl^*Cγ1*^Cre^ B cells at the indicated time points of iPC culture (**E**). (**F**) Bar graph summarizing percentages of Annexin V^+^7-AAD^+^cells in (**E**). Data from three independent biological replicates (iPC D2: *P *= 0.0013). (**G**) Flow cytometry analysis of IgG1^+^ and IgE^+^ cells among *Dhx29*^fl/fl^ and *Dhx29*^fl/fl^*Cγ1*^Cre^ B cells in iPC culture at day 4. Dot plots summarizing the percentage (left) and number (right) of IgG1^+^ and IgE^+^ cells. Data from three independent biological replicates (IgG1^+^ of iPC cells (%): *P* = 9.46E-06; IgE^+^ of iPC cells (%): *P* = 4.47E-06; IgG1^+^ cell number ( × 10^6^): *P* = 0.0003). (**H**) Quantitative RT-PCR analysis of germline IgG1 transcripts in *Cγ1*^Cre^ and *Dhx29*^fl/fl^*Cγ1*^Cre^ B cells at iGCB day 4 and iPC culture. Data from three independent biological replicates (iPC D1: *P* = 0.0101; iPC D2: *P* =  0.0328; iPC D4: *P* =  0.0095). Small horizontal lines indicate the mean ( ± SD). Statistical significance was determined by unpaired, two-tailed Student’s *t* test in (**C**, **F**, **G**, **H**). *P  <  0.05, **P  <  0.01, ***P < 0.001, ****P < 0.0001. Source data are available online for this figure.

**Figure EV2:**
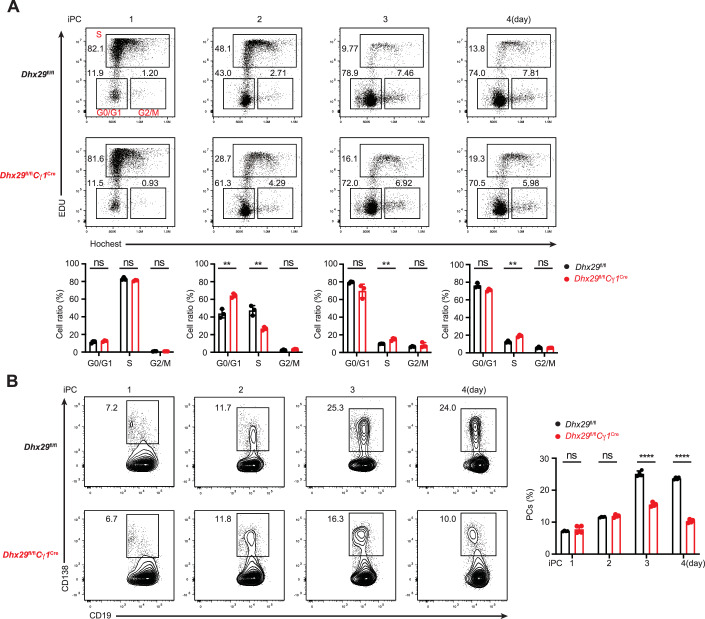
Proliferation and differentiation of *Dhx29*-deficient B cells. Flow cytometry analysis of cell cycle progression (**A**) and percentages of CD19^+^CD138^+^ iPC cells (**B**) among *Dhx29*^fl/fl^ and *Dhx29*^fl/fl^*Cγ1*^Cre^ B cells at the indicated time points of iPC culture. Upper, representative FACS plots. Lower, bar graphs summarizing percentages of cells at indicated phases of cell cycle (**A**) and percentages of CD19^+^CD138^+^ iPC cells (**B**). Data from three or four independent biological replicates (iPC D2 G0/G1: *P* =  0.0030; iPC D2 S: *P* =  0.0037; iPC D3 S: *P* =  0.0053; iPC D4 S: *P* =  0.0027; iPC D3 PCs (%): *P* =  1.67E-06; iPC D4 PCs (%): *P* =  1.29E-08). Small horizontal lines indicate the mean ( ± SD). Statistical significance was determined by unpaired, two-tailed Student’s *t* test in (**A**, **B**). **P  <  0.01, ****P < 0.0001.

We further dissected the roles of DHX29 in B-cell immune responses in the in vitro culture system. B cells from *Dhx29*^fl/fl^*Cγ1*^Cre^ and *Dhx29*^fl/fl^ mice were co-cultured with 40LB cells in the presence of IL-4 and IL-21 to differentiate into iGCB and iPC cells, respectively (Fig. 4A). As shown in Fig. 4B, DHX29 expression was robustly induced in the first 2 days of iGCB culture and remained at high levels afterward. In *Dhx29*^fl/fl^*Cγ1*^Cre^ B cells, Cre-mediated deletion occurred around day 2–3, and DHX29 protein largely disappeared by day 3 and 4. In this culture system, *Dhx29* deficiency led to significantly reduced percentage and number of iPC cells, with no obvious effect on iGCB cells (Fig. 4C). We examined cell proliferation, cell cycle progression, and cell death at every single day during the 4-day period of iPC differentiation. As shown in Figs. 4D–F and EV2A, while *Dhx29*^fl/fl^*Cγ1*^Cre^ and *Dhx29*^fl/fl^ B cells were highly similar at day 1, 3, and 4, the former showed delayed G1-S progression and increased death at day 2. Notably, *Dhx29*^fl/fl^*Cγ1*^Cre^ B cells exhibited a significant reduction in germline transcript of IgG1 (Fig. 4H) and B-cell differentiation into CD138^+^ plasma cells (Fig. EV2B), resulting in a drastic decrease in the percentage and number of IgG1^+^ iPC cells at the end of culture (Fig. 4G). These results, together with the much-reduced NP-IgG1 antibody levels and NP-IgG1 antibody-secreting cell numbers observed in *Dhx29*^fl/fl^*Cγ1*^Cre^ mice (Fig. 3A,B), demonstrate that DHX29 plays an indispensable role in class switch to IgG1 and plasma cell differentiation.

### DHX29 controls the expression of BLIMP1

**Figure 5:**
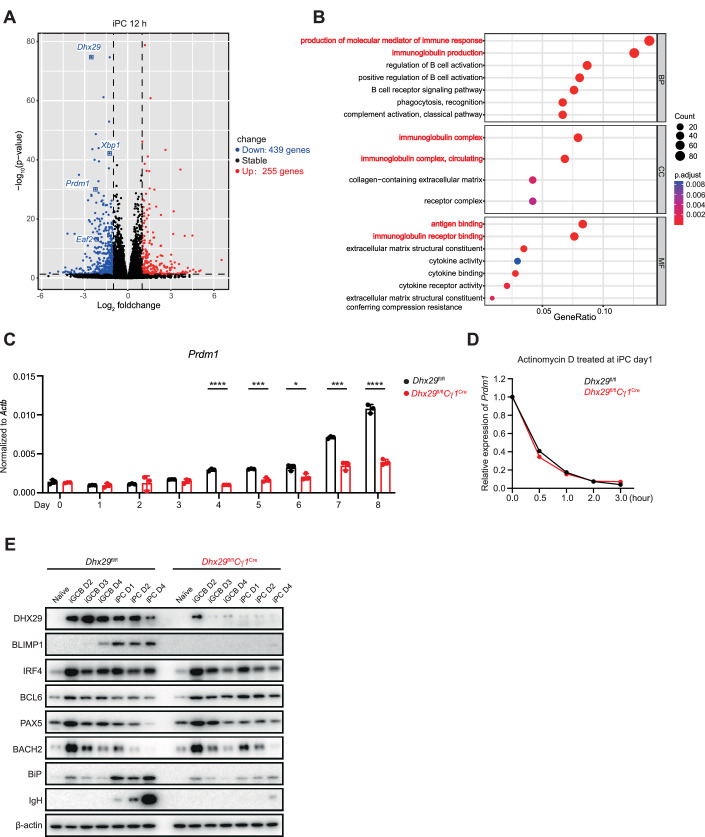
Diminished transcription of *Prdm1* in *Dhx29*-deficient B cells. (**A**) Volcano plots showing differentially expressed genes (DEGs) in *Dhx29*^fl/fl^*Cγ1*^Cre^ vs *Dhx29*^fl/fl^ B cells at iPC 12 h. Data from three independent biological replicates. Differential expression analysis was performed using DESeq2, with statistical significance determined by a two-sided Wald test. (**B**) Gene ontogeny (GO) analysis of differentially expressed genes in (**A**), performed using clusterProfiler with the hypergeometric test. *P* values were adjusted using the Benjamini–Hochberg method. BP biological processes, CC cellular components, MF molecular function. (**C**) Quantitative RT-PCR analysis of *Prdm1* mRNA in *Dhx29*^fl/fl^ and *Dhx29*^fl/fl^*Cγ1*^Cre^ B cells at the indicated time points. Data from three independent biological replicates (Day 4: *P* =  3.40E-05; Day 5: *P* =  0.0009; Day 6: *P* =  0.0218; Day 7: *P* =  0.0004; Day 8: *P* =  5.91E-05). (**D**) Quantitative RT-PCR analysis of *Prdm1* mRNA in *Dhx29*^fl/fl^ and *Dhx29*^fl/fl^*Cγ1*^Cre^ B cells at the indicated time points after actinomycin D treatment. *Prdm1* mRNA level at time 0 was set as 1. Data from three independent biological replicates. (**E**) Immunoblot analysis of indicated proteins in *Dhx29*^fl/fl^ and *Dhx29*^fl/fl^*Cγ1*^Cre^ B cells during iGCB culture. Small horizontal lines indicate the mean ( ± SD). Statistical significance was determined by unpaired, two-tailed Student’s *t* test in(**C**). *P  <  0.05, ***P  <  0.001, ****P < 0.0001. Source data are available online for this figure.

To elucidate the molecular mechanism underlying DHX29 regulation of plasma cell differentiation and class switch recombination, we performed RNA-sequencing (RNA-seq) analysis of iPC 12 h cells harvested from in vitro cultured *Dhx29*^fl/fl^ and *Dhx29*^fl/fl^*Cγ1*^Cre^ B cells. 439 genes were downregulated and 255 genes were upregulated in *Dhx29*^fl/fl^*Cγ1*^Cre^ iPC 12 h cells (Fig. 5A). Notably, the mRNA levels of *Prdm1* (encoding BLIMP1) and its downstream target genes *Xbp1* and *Eaf2* were significantly decreased in DHX29-deficient cells (Fig. 5A). While BLIMP1 is the master regulator of plasma cell differentiation, XBP1 controls UPR, drives ER expansion, and induces the expression of many genes involved in ER protein homeostasis. EAF2 is a critical activator of ELL2, which promotes choice of the distal *Igh* polyadenylation site and generation of *Igh* mRNA encoding the secreted antibody (Martincic et al, 2009; Park et al, 2014).

**Figure EV3:**
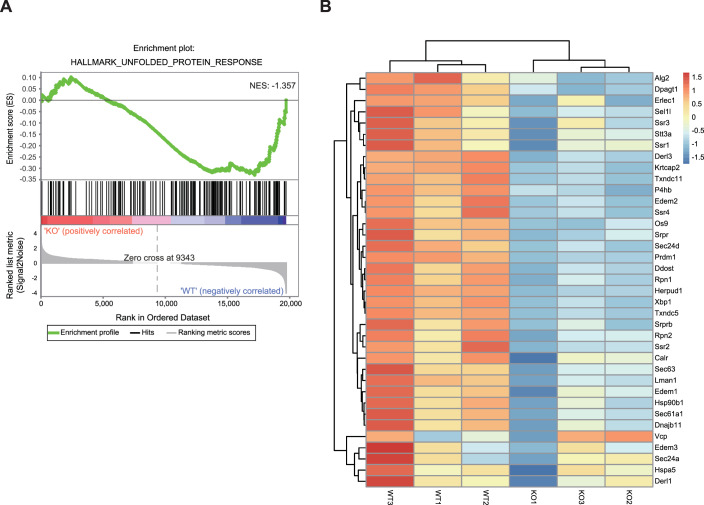
Impaired expression of genes related to ER protein processing in *Dhx29*-deficient B cells. (**A**) Gene enrichment pathway identified by GESA analysis. NES, normalized enrichment score. (**B**) Heatmap of select genes related to ER protein processing identified by RNA-Seq in Fig. 5A.

We performed Gene Ontology (GO) analysis of differentially expressed genes. Immunoglobulin production and immunoglobulin receptor binding were among the enriched gene sets (Fig. 5B). Gene set enrichment analysis (GSEA) revealed that many downregulated genes were mapped to the UPR pathway (Fig. EV3). Quantitative RT-PCR analysis showed that expression of the *Prdm1* mRNA was severely impaired in *Dhx29*^fl/fl^*Cγ1*^Cre^ B cells throughout the in vitro culture stages (Fig. 5C). When iPC day 1 cells were treated with actinomycin D to block synthesis of new mRNAs and to examine turnover of existing mRNAs, *Prdm1* mRNA showed similar half-life in *Dhx29*^fl/fl^*Cγ1*^Cre^ and *Dhx29*^fl/fl^ B cells (Fig. 5D), suggesting that decreased *Prdm1* mRNA levels in *Dhx29*^fl/fl^*Cγ1*^Cre^ B cells were not caused by increased mRNA decay in these cells. We speculated that reduced transcription led to impaired *Prdm1* expression. Accordingly, the BLIMP1 protein was completely absent during in vitro plasma cell differentiation of *Dhx29*^fl/fl^*Cγ1*^Cre^ B cells, accompanied by markedly decreased expression of BiP and IgH proteins and a slight decrease in IRF4 protein (Fig. 5E). Conversely, the expression of BCL6 and BACH2 proteins remained at higher levels in *Dhx29*-deficient B cells during the iPC stage of culture (Fig. 5E). Taken together, DHX29 promotes plasma cell differentiation by regulating the expression of key transcription factors controlling this cellular process.

### TCF3 and TLE3 mediate DHX29 regulation of plasma cell differentiation

**Figure 6:**
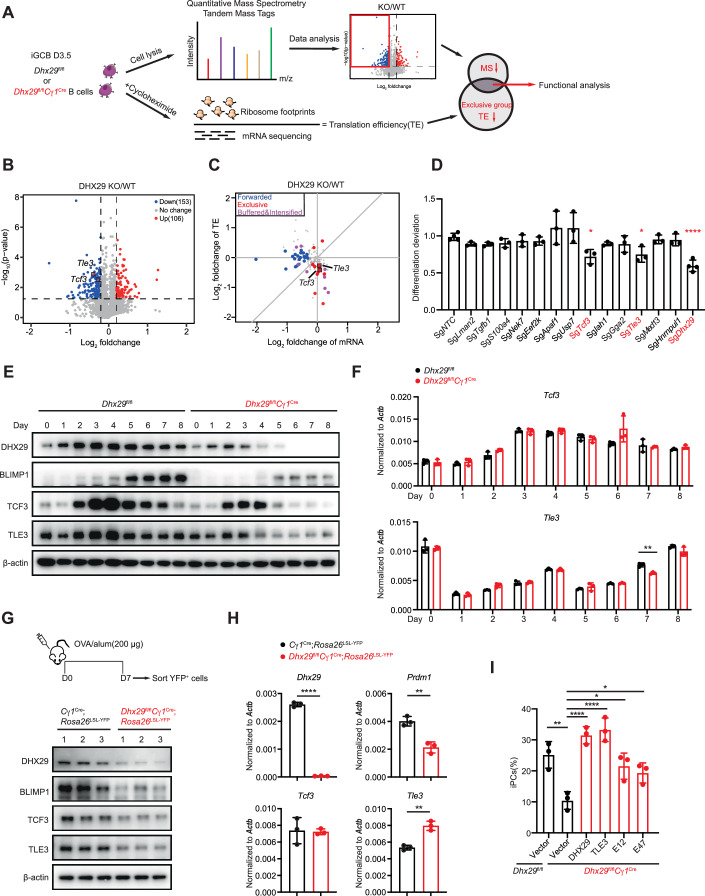
TCF3 and TLE3 mediate DHX29 regulation of plasma cell differentiation. (**A**) Experimental outline for identifying functional target genes of DHX29. (**B**) Volcano plots showing differentially expressed genes (DEGs) based on mass spectrometry analysis of *Dhx29*^fl/fl^*Cγ1*^Cre^ and *Dhx29*^fl/fl^ B cells at iGCB culture day 3.5. *Tcf3* and *Tle3* were highlighted. Data from three independent biological replicates. (**C**) Scatter plots showing log2 fold changes in translation efficiency (TE) and mRNA expression levels of downregulated genes identified in (**B**). Forwarded (blue): genes are mainly changed at the mRNA level; Exclusive (red): mRNA level does not change, while translation efficiency changes; Buffered and Intensified (purple): Buffered refers to genes whose mRNA level and translation efficiency change in opposite directions. Intensified refers to genes whose mRNA level and translation efficiency change in the same direction. (**D**) Differentiation deviation of Cas9-GFP^+^ B cells transduced with retroviruses encoding indicated sgRNAs and analyzed at iPC day 4. Each symbol represents a distinct sgRNA (Sg*Tcf3* vs Sg*NTC*: *P* = 0.0172; Sg*Tle3* vs Sg*NTC*: *P* =  0.0445; Sg*Dhx29* vs Sg*NTC*: *P* =  6.60E-05). (**E**) Immunoblot analysis of indicated proteins in *Dhx29*^fl/fl^ and *Dhx29*^fl/fl^*Cγ1*^Cre^ B cells during iGCB culture. (**F**) Quantitative RT-PCR analysis of *Tcf3* and *Tle3* mRNA expression in *Dhx29*^fl/fl^ and *Dhx29*^fl/fl^*Cγ1*^Cre^ B cells during iGCB culture. Data from three independent biological replicates (*Tle3* Day 7: *P* =  0.0038). (**G**, **H**) YFP^+^ cells were sorted from *Cγ1*^Cre^; *Rosa26*^LSL-YFP^ and *Dhx29*^fl/fl^*Cγ1*^Cre^; *Rosa26*^LSL-YFP^ mice immunized with OVA/alum. Expression of indicated proteins was analyzed by immunoblot (**G**). Relative mRNA expression of the indicated genes was measured by qRT-PCR (**H**). Each symbol represents an individual mouse (*n* = 3 per group). (*Dhx29*: *P* =  5.67E-07; *Prdm1*: *P* = 0.0034; *Tle3*: *P* = 0.0019). (**I**) *Dhx29*^fl/fl^ and *Dhx29*^fl/fl^*Cγ1*^Cre^ B cells were transduced with retroviruses encoding the indicated genes at iGCB day 2.5. The percentage of iPCs (GFP^+^CD19^+^CD138^+^) was analyzed by flow cytometry at iPC day 4. Data were pooled from three independent experiments (*Dhx29*^fl/fl^-Vector vs *Dhx29*^fl/fl^*Cγ1*^Cre^-Vector: *P* = 0.0013; *Dhx29*^fl/fl^*Cγ1*^Cre^-DHX29 vs *Dhx29*^fl/fl^*Cγ1*^Cre^-Vector: P =  4.84E-05; *Dhx29*^fl/fl^*Cγ1*^Cre^-TLE3 vs *Dhx29*^fl/fl^*Cγ1*^Cre^-Vector: *P* =  2.17E-05; *Dhx29*^fl/fl^*Cγ1*^Cre^-E12 vs *Dhx29*^fl/fl^*Cγ1*^Cre^-Vector: *P* = 0.0106; *Dhx29*^fl/fl^*Cγ1*^Cre^-E47 vs *Dhx29*^fl/fl^*Cγ1*^Cre^-Vector: *P* = 0.0403). Small horizontal lines indicate the mean ( ± SD.). Statistical significance was determined by One-way ANOVA in (**D**, **I**); unpaired, two-tailed Student’s *t* test in (**F**, **H**). *P  <  0.05, **P  <  0.01, ****P < 0.0001. Source data are available online for this figure.

**Figure EV4:**
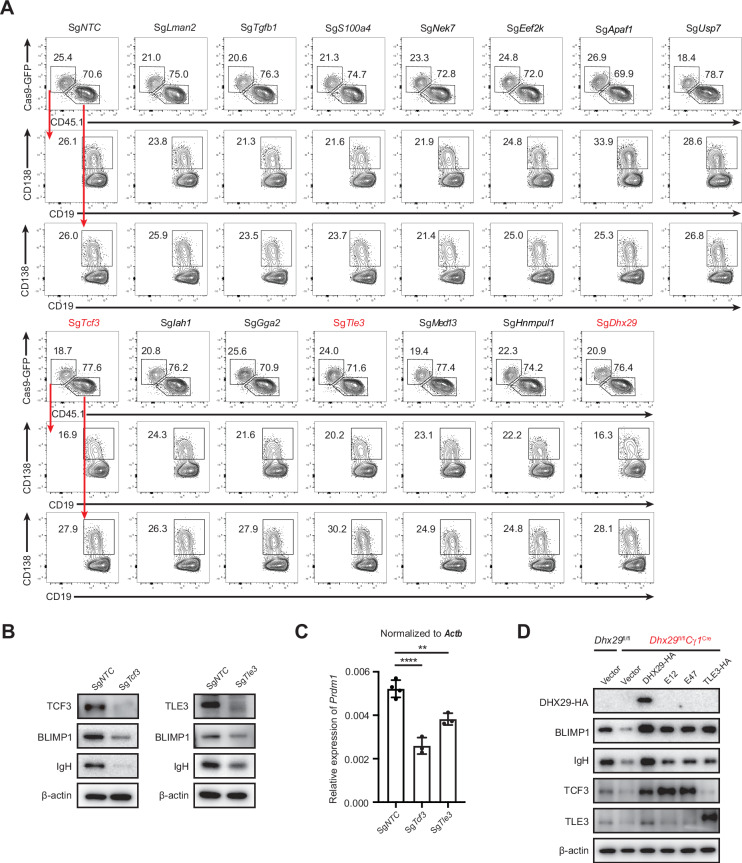
Functional analysis of potential DHX29 target genes identified by mass spectrometry and ribosome profiling. (**A**) Naïve B cells from Cas9-GFP (CD45.2^+^) and CD45.1^+^ wild-type mice were mixed in a 1:1 ratio, cultured in the iGCB system, transduced with retroviruses encoding indicated sgRNAs, and induced for iPC differentiation for 4 days. The percentages of CD45.1^+^ and Cas9-GFP^+^ cells, as well as iPCs (CD19^+^CD138^+^), were analyzed by flow cytometry. (**B**) Immunoblot analysis of indicated proteins in Cas9-GFP^+^ B cells transduced with retroviruses encoding *Tcf3* or *Tle3* sgRNA and analyzed at iPC day 4. (**C**) Quantitative RT-PCR analysis of BLIMP1 mRNA level in Cas9-GFP^+^ B cells transduced with retroviruses encoding NTC, *Tcf3*, or *Tle3* sgRNA and analyzed at iPC day 1. Data from three or four independent biological replicates (Sg*Tcf3* vs Sg*NTC*: *P* =  5.56E-05; Sg*Tle3* vs Sg*NTC*: *P* =  0.0027). (**D**) *Dhx29*^fl/fl^ and *Dhx29*^fl/fl^*Cγ1*^Cre^ B cells were transduced with retroviruses encoding the indicated genes at iGCB day 2.5. GFP^+^ cells were sorted and analyzed at iPC day 4 by immunoblot for HA-tagged DHX29 (by anti-HA antibody), BLIMP1, IgH, TCF3, and TLE3. Small horizontal lines indicate the mean ( ± SD). Statistical significance was determined by one-way ANOVA in (**C**). **P  <  0.01, ****P < 0.0001.

To further elucidate the molecular mechanism underlying DHX29 regulation of BLIMP1 transcription, we performed mass spectrometry (MS), RNA-seq, and Ribo-seq analysis of *Dhx29*^fl/fl^ and *Dhx29*^fl/fl^*Cγ1*^Cre^ B cells at iGCB day 3.5 (Fig. 6A–C). We chose this time point to identify early events that are controlled by DHX29 and lead to BLIMP1 downregulation in *Dhx29*-deficient B cells, as downregulation of BLIMP1 mRNA and protein manifested at iGCB day 4 (Fig. 5C,E). It was previously reported that DHX29 is an RNA helicase controlling translational initiation of mRNAs with structured 5’UTRs (Pisareva et al, 2008). We speculate that *Dhx29* deficiency would lead to downregulation of translation efficiency and protein levels of its target genes, without much effect on target mRNA expression levels. MS analysis showed that 153 proteins were downregulated and 106 proteins were upregulated in *Dhx29*^fl/fl^*Cγ1*^Cre^ B cells (Fig. 6B). Among the 153 downregulated proteins, RNA-seq and Ribo-seq identified 13 genes with reduced translation efficiency but unaltered mRNA levels (Fig. 6C). We performed CRISPR/Cas9-mediated functional analysis of those 13 genes to identify the ones regulating plasma cell differentiation (Figs. 6D and EV4A). Knockout of *Tcf3* and *Tle3* showed effect similar to, but milder than, *Dhx29* knockout in terms of impaired plasma cell differentiation and reduced BLIMP1 expression (Figs. 6D and EV4A–C). Immunoblot and qRT-PCR analyses confirmed that *Dhx29* deficiency led to significant reductions in the protein levels of TCF3 and TLE3, but little effect on their mRNA levels (Fig. 6E,F). We also assessed this regulation in vivo. YFP^+^ DHX29 KO cells were sorted from immunized *Dhx29*^fl/fl^*Cγ1*^Cre^; *Rosa26*^LSL-YFP^ mice, and protein expression was examined. Consistent with in vitro data, DHX29 deletion led to a substantial reduction in TCF3 and TLE3 protein expression without a decrease in their mRNA levels (Fig. 6G,H). Both the mRNA and protein levels of BLIMP1 were decreased. Importantly, retroviral expression of TCF3 (E12 and E47 isoforms) and TLE3 largely restored BLIMP1 expression and plasma cell differentiation of *Dhx29*-deficient B cells (Figs. 6I and EV4D), suggesting that TCF3 and TLE3 are functional target genes of DHX29.

It has been reported that TCF3 functions as a transcription factor and plays an indispensable role in the development of GCB and plasma cells during immune responses. By regulating 3′ *Igk* and *Igh* enhancers and a distal element at the *Prdm1* locus, TCF3 controls CSR in plasmablasts (Gloury et al, 2016; Wohner et al, 2016; Wu et al, 2022). Consistent with a previous study reporting that TCF3 is the dominant E protein controlling CSR to IgG1, the percentage and number of IgG1^+^ cells at iPC day 4 were significantly decreased when *Dhx29*^fl/fl^*Cγ1*^Cre^ B cells were differentiated in the 40LB in vitro culture system (Fig. 4G).

**Figure 7:**
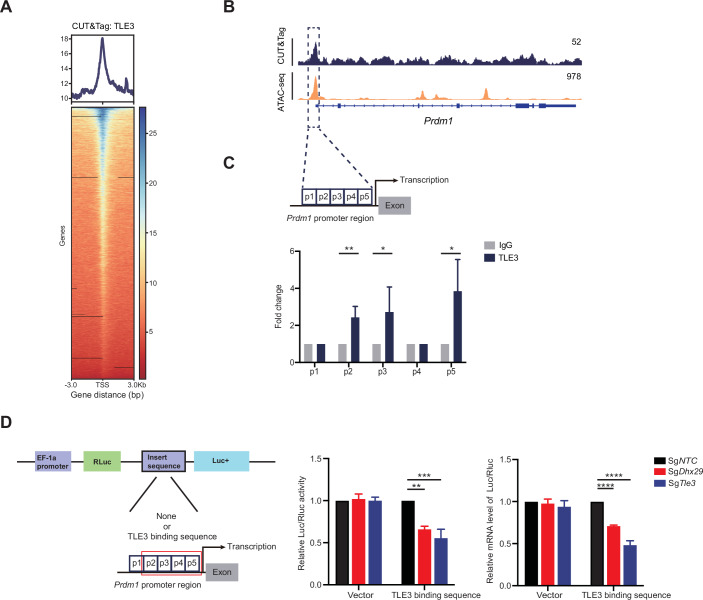
TLE3 associates with the promoter region of *Prdm1* and promotes its transcription. (**A**) CUT&Tag density heatmap of TLE3 binding peaks within 3 kb around transcription start sites. (**B**) Representative track view shows TLE3 binding peaks at the *Prdm1* genomic locus. (**C**) TLE3 binding to the *Prdm1* promoter region was measured by CUT&Tag followed by PCR. Data from four independent biological replicates (P2: *P* =  0.0027; P3: *P* =  0.0429; P5: *P* =  0.0156). (**D**) Luciferase reporter assay of DHX29 and TLE3 regulation of the *Prdm1* promoter activity. Schematic representation of the psiCheck2 reporter harboring TLE3 binding sequence in the *Prdm1* promoter region (left). Luciferase reporter activity (middle) and quantitative RT-PCR analysis of Firefly and Renilla luciferase mRNA levels (right) in Cas9-GFP^+^ B cells transduced with retroviruses encoding the indicated sgRNA were analyzed at day 5 of iGCB culture. Data were pooled from three independent experiments (Relative Luc/Rluc activity: Sg*Dhx29* vs Sg*NTC* *P* =  0.0011, Sg*Tle3* vs Sg*NTC* *P* =  0.0003; Relative mRNA level of Luc/Rluc: Sg*Dhx29* vs Sg*NTC* *P* =  3.73E-05, Sg*Tle3* vs Sg*NTC* *P* =  1.27E-06). Small horizontal lines indicate the mean ( ± SD.). Statistical significance was determined by unpaired, two-tailed Student’s *t* test in (**C**); one-way ANOVA in (**D**). *P  <  0.05, **P  <  0.01, ***P < 0.001, ****P < 0.0001. Source data are available online for this figure.

A previous study reported that TLE3 cooperates with Hhex to regulate the transcriptional circuitry governing GCB cell differentiation into memory B cells (Laidlaw et al, 2020). It is interesting to note that *Dhx29*^fl/fl^ *Cγ1*^Cre^ mice also showed decreased abundance of GC-dependent NP-specific memory B cells after immunization with NP-OVA/alum/LPS (Fig. 3C). Recent studies showed that TLE3 functions as a coactivator for Tbet to increase chromatin opening at CD8^+^ effector memory cell-specific sites and to activate transcription of CD8^+^ effector memory cell signature genes, including *Ifng*, *Gzma*, *Gzmb*, *Cx3cr1*, *Irf4*, and *Prdm1* (Zhao et al, 2024). We therefore examined the association of TLE3 with genomic DNA in iGCB cells by CUT&TAG assay. As shown in Fig. 7A, TLE3 binding sites were highly enriched near transcription start sites (TSS). CUT&Tag followed by PCR confirmed direct binding of TLE3 to the promoter regions of *Prdm1* (Fig. 7B,C). We further examined the regulatory role of TLE3 binding to the *Prdm1* promoter in a dual-luciferase reporter assay. The genomic DNA region harboring TLE3 binding sites (p2-p5 region) was cloned into the psiCheck2 reporter plasmid and placed immediately upstream of the firefly luciferase reporter gene (Luc + ) (Fig. 7D). Cas9-GFP^+^ B cells were co-transduced by retroviruses encoding the psiCheck2 reporter plasmid and NTC, Sg*Dhx29*, or Sg*Tle3*. Subsequent analyses showed that *Dhx29* and *Tle3*-deficiency significantly reduced the activity and mRNA level of the firefly luciferase reporter gene (Fig. 7D). Taken together, these results demonstrate that TCF3 and TLE3 are crucial downstream mediators of DHX29 regulation of plasma cell differentiation.

### DHX29 promotes translation of *Tcf3* and *Tle3* mRNAs

**Figure EV5:**
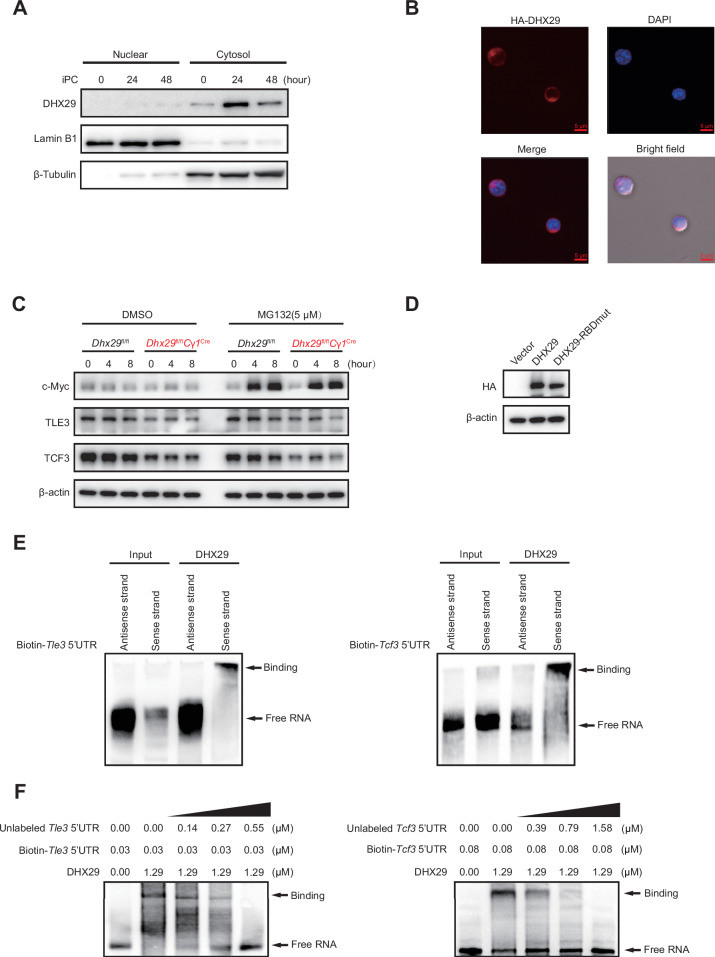
DHX29 promotes *Tcf3* and *Tle3* translation in the cytosol. (**A**) Immunoblot analysis of DHX29 in the cytosolic and nuclear fractions of B cells at different time points of iPC culture. Lamin B1 and β-tubulin were used as control for nuclear and cytosolic proteins, respectively. (**B**) Representative immunofluorescence images of B cells transduced with retroviruses encoding HA-tagged DHX29 and stained for HA (red) and DAPI (blue, nuclear staining). Scale bar, 5 μm. (**C**) Immunoblot analysis of *Dhx29*^fl/fl^ and *Dhx29*^fl/fl^*Cγ1*^Cre^ B cells treated with DMSO and MG132 at iGCB day 3.5 for indicated amounts of time. c-Myc was used as a control. (**D**) Immunoblot analysis of DHX29 in HEK293T cells transduced with retroviruses encoding HA-tagged DHX29 or its RBD binding domain deleted mutant (DHX29-RBDmut). (**E**) EMSA analysis of DHX29 binding to the 5’UTRs of *Tle3* (left) and *Tcf3* (right). 1.29 μM DHX29 protein was incubated with 0.03 μM *Tle3* 5’UTR or 0.08 μM *Tcf3* 5’UTR labeled with biotin. The mixtures were resolved on a native TBE gel. The antisense strand of each 5’UTR was used as a negative control. (**F**) Competition-binding assay. Increasing concentrations of unlabeled *Tle3* or *Tcf3* 5’UTR were added to the reaction mixture in (**E**). The mixtures were resolved on a native TBE gel.

**Figure 8:**
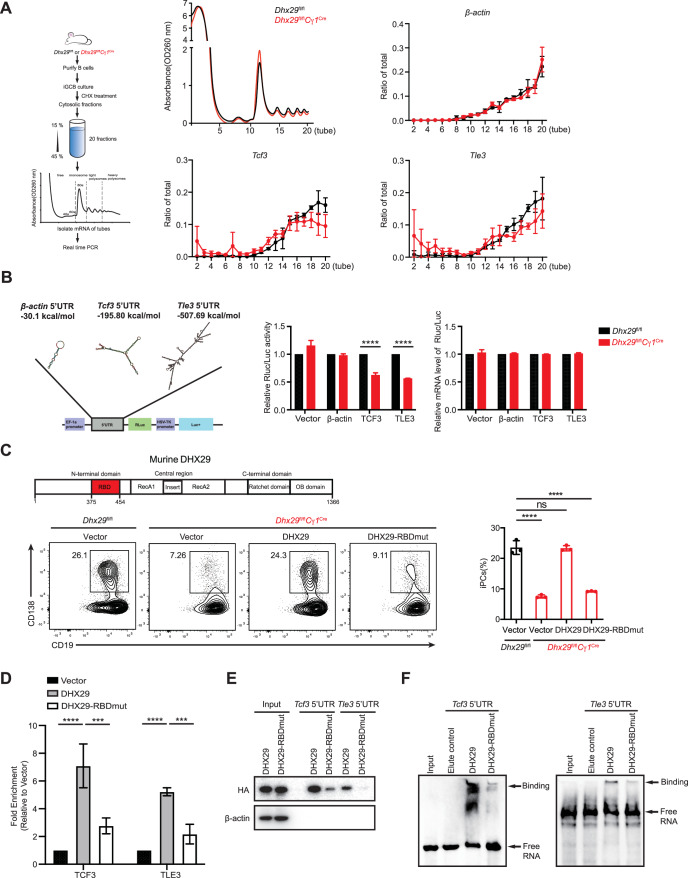
DHX29 promotes *Tle3* and *Tcf3* translation in B cells. (**A**) Polysome profiling analysis of *Dhx29*^fl/fl^ and *Dhx29*^fl/fl^*Cγ1*^Cre^ B cells at day 3.5 of iGCB culture. The distribution of *Tcf3*, *Tle3*, and *β-actin* mRNAs in the sucrose gradient of *Dhx29*^fl/fl^ and *Dhx29*^fl/fl^*Cγ1*^Cre^ B cells. Data were pooled from three independent experiments. (**B**) Luciferase reporter assay of DHX29 regulation of various 5’UTRs. Schematic representation of the psiCheck2 reporter harboring 5’UTRs of indicated genes (left). Luciferase reporter activity (middle) and quantitative RT-PCR analysis of Firefly and Renilla luciferase mRNA levels (right) in *Dhx29*^fl/fl^ and *Dhx29*^fl/fl^*Cγ1*^Cre^ B cells transduced with retroviruses encoding indicated reporters were analyzed at day 4 of iGCB culture. Data were pooled from three or four independent experiments (Relative Luc/Rluc activity: *Tcf3* 5’UTR *Dhx29*^fl/fl^ *Cγ1*^Cre^vs *Dhx29*^fl/fl^ *P* =  2.72E-06, *Tle3* 5’UTR *Dhx29*^fl/fl^ *Cγ1*^Cre^ vs *Dhx29*^fl/fl^ *P* =  3.43E-09). (**C**) Restoration of plasma cell differentiation of *Dhx29*-deficient B cells by wild-type and mutant DHX29. Schematic representation of DHX29 protein domain structure (upper left). *Dhx29*^fl/fl^ and *Dhx29*^fl/fl^*Cγ1*^Cre^ B cells were transduced with retroviruses encoding the indicated proteins at iGCB culture day 2.5, and the percentage of iPCs (GFP^+^CD19^+^CD138^+^) was analyzed at iPC day 4. Lower left, representative FACS plots. Right, bar graph summarizing percentages of iPCs. Data from three independent biological replicates (*Dhx29*^fl/fl^*Cγ1*^Cre^-Vector vs *Dhx29*^fl/fl^-Vector: *P* =  6.90E-07; *Dhx29*^fl/fl^*Cγ1*^Cre^-DHX29 vs *Dhx29*^fl/fl^-Vector: *P* = 0.9788; *Dhx29*^fl/fl^*Cγ1*^Cre^-DHX29-RBDmut vs *Dhx29*^fl/fl^-Vector: *P *=  1.64E-06). (**D**) RIP assay assessing DHX29-mRNA interactions. Quantitative RT-PCR analysis of indicated mRNAs pulled down by HA antibody from *Dhx29*^fl/fl^*Cγ1*^Cre^ B cells transduced with retroviruses encoding empty vector, HA-DHX29, or HA-DHX29-RBDmut. Data were pooled from three or four independent experiments (TCF3: Vector vs DHX29 *P* = 1.84E-05; DHX29-RBDmut vs DHX29 *P* = 0.0003; TLE3: Vector vs DHX29 *P* = 4.34E-05; DHX29-RBDmut vs DHX29 *P* = 0.0003). (**E**) Total cell lysates of *Dhx29*^fl/fl^*Cγ1*^Cre^ B cells transduced with retroviruses encoding HA-DHX29 and HA-DHX29-RBDmut were precipitated by biotinylated RNA encoding *Tcf3* and *Tle3* 5’UTRs and analyzed by immunoblot with HA antibody. (**F**) EMSA analysis of interactions between biotinylated RNA encoding *Tcf3* 5’UTRs (0.05 μM) and *Tle3* 5’UTRs (0.02 μM) with recombinant DHX29 (0.43 μM) and DHX29-RBDmut (0.46 μM) proteins. The vector-control eluate obtained during protein purification (see “Methods”) was used as a negative control. Small horizontal lines indicate the mean ( ± SD.). Statistical significance was determined by unpaired, two-tailed Student’s *t* test in (**B**); one-way ANOVA in (**C**, **D**). ***P < 0.001, ****P < 0.0001. Source data are available online for this figure.

We further investigated molecular mechanisms underlying DHX29 regulation of TCF3 and TLE3 expression. Consistent with its primary role in translation control, DHX29 protein was mainly found in the cytosolic compartment of iPC cells (Fig. EV5A,B). Treatment of *Dhx29*-deficient B cells with MG132, a proteasome inhibitor, did not restore TCF3 and TLE3 protein expression (Fig. EV5C), indicating that reduced TCF3 and TLE3 protein levels in *Dhx29*-deficient B cells were not caused by accelerated protein degradation. We then performed polysome profiling analysis of *Dhx29*-deficient B cells to examine the role of DHX29 in translation. As shown in Fig. 8A, there was a slight reduction in polysome fractions accompanied by accumulation of 80S monosome fractions in *Dhx29*-deficient B cells, indicating a mild decrease in the global translation rate. The distribution of *Tcf3* and *Tle3* mRNAs shifted from heavy polysomes to light polysomes and monosomes (Fig. 8A), consistent with reduced translation efficiency of these mRNAs (Fig. 6C). DHX29 was originally discovered as an RNA helicase controlling translational initiation of mRNAs with structured 5’UTRs (Pisareva et al, 2008). The 5’UTRs of *Tcf3* and *Tle3* mRNAs were therefore examined for the presence of complex secondary structures. Indeed, the RNAfold web server identified potential secondary structures in those 5’UTRs with free energy much lower than that of *β-actin* 5’UTR (Fig. 8B). We cloned those 5’UTRs into the psiCheck2 reporter plasmid and placed them immediately upstream of the Renilla luciferase gene (RLuc). *Dhx29*^fl/fl^ and *Dhx29*^fl/fl^*Cγ1*^Cre^ B cells were transduced with retroviruses encoding those reporter plasmids (Fig. 8B). Reporter assay showed that *Dhx29*-deficiency substantially reduced the activity of Renilla luciferase reporter gene containing the 5’UTRs of *Tcf3* and *Tle3* while exhibiting little effect on their mRNA levels (Fig. 8B).

The mouse DHX29 protein contains a dsRNA-binding domain (RBD, amino acids 375–454) in its N-terminus (Dhote et al, 2012). To determine whether RNA-binding activity of DHX29 is important for its function in B cells, we generated retroviruses encoding wild-type and mutant DHX29 with amino acids 375–454 deleted (termed DHX29-RBDmut) (Figs. 8C and EV5D). *Dhx29*^fl/fl^*Cγ1*^Cre^ B cells were transduced with those retroviruses during iGCB culture and plasma cell differentiation was subsequently analyzed. As shown in Figs. 8C and EV5D, while retroviral expression of wild-type DHX29 restored plasma cell differentiation of *Dhx29*^fl/fl^*Cγ1*^Cre^ B cells, DHX29-RBDmut showed little effect, indicating a critical role of the RNA-binding ability of DHX29 in controlling plasma cell differentiation. RIP assay and biotin-labeled RNA pull-down assay showed that DHX29 bound to 5’ UTR of *Tcf3* and *Tle3* mRNAs, while DHX29-RBDmut exhibited much reduced binding (Fig. 8D,E). Electrophoretic mobility shift assay (EMSA) further confirmed that wild-type DHX29 protein directly interacted with both 5’UTRs and that those interactions were attenuated upon deletion of DHX29 RNA-binding domain (Figs. 8F and EV5E,F). Taken together, these results show that DHX29 controls plasma cell differentiation by binding to 5’UTRs of *Tcf3* and *Tle3* mRNAs and promoting their translation.

## Discussion

In this study, we performed CRISPR/Cas9-mediated functional analysis of RNA helicases in an in vitro system of plasma cell differentiation and identified DHX29 as a critical regulator of this process. Mouse genetics studies showed that DHX29 plays important roles in GCB formation, class switch to IgG1, plasma cell differentiation, and antibody production. Molecular analysis revealed that DHX29 binds to 5’UTRs of *Tcf3* and *Tle3* mRNAs and promotes their translation. TCF3 and TLE3 then associate with the promoter region of the *Prdm1* gene to facilitate its transcription. In the absence of DHX29, B cells exhibit normal proliferation but impaired CSR to IgG1 and differentiation into plasma cells, resulting in severely compromised antibody production. Ectopic expression of TCF3 and TLE3 largely restored plasma cell differentiation of *Dhx29*-deficient B cells, demonstrating the crucial importance of TCF3 and TLE3 in mediating DHX29 function in B cells and unraveling a previously unrecognized role of TLE3 in controlling plasma cell differentiation.

This study highlights the functional importance of RNA helicases in controlling translation initiation (Parsyan et al, 2011). During this process, the 40S ribosome subunit is recruited to the 5’ terminus of mRNA and scans 5’UTR to find the initiation codon, where it is joined by the 60S ribosome subunit to form the translation-competent 80S ribosome. Secondary structures in the 5’UTR can impede binding of and scanning by the 40S ribosome. It is thought that RNA helicases unwind secondary structures in the 5’UTR to promote translation initiation. We speculate that there exist different pools of 40S ribosomes, with each pool associated with different RNA helicases and regulating translation initiation of different sets of mRNAs. Recent studies from us and another group demonstrated that eIF4A1 controls global translation and protein synthesis (Du et al, 2025; Screen et al, 2024), while this study showed that DHX29 controls translation initiation of a specific set of mRNAs in B cells. The functional difference between those two RNA helicases may be caused by different mechanisms underlying their activities and different stoichiometry between those RNA helicases and the 40S ribosome. eIF4A1 strictly requires eIF4G and eIF4B for its activity, whereas DHX29 requires the 40S ribosome, which stimulates its ATPase activity (Harms et al, 2014; Pisareva et al, 2008). In rabbit reticulocyte lysate (RRL), DHX29 is wholly associated with 40S ribosomes, but DHX29-bound 40S ribosomes constitute only ~10% of all 40S ribosomes (Pisareva et al, 2008). It is likely that in B cells, DHX29-bound 40S ribosomes also constitute a relatively small fraction of 40S ribosomes and control translation initiation of a specific set of mRNAs that include *Tcf3* and *Tle3*, while eIF4A1-bound 40S ribosomes constitute a large fraction of 40S ribosomes and exert global control of translation and protein synthesis. The mechanistic and stoichiometric differences underlie functional differences: eIF4A1 controls B-cell proliferation (Du et al, 2025; Screen et al, 2024), while DHX29 controls GCB formation and plasma cell differentiation with little effect on proliferation.

Previous studies reported that TCF3 functions as a transcription factor and plays an indispensable role in the generation of GCB and plasma cells during immune responses, and that TCF3 is the dominant E protein controlling CSR to IgG1 (Gloury et al, 2016; Wohner et al, 2016; Wu et al, 2022). DHX29 regulation of translation initiation of *Tcf3* mRNA should contribute substantially to DHX29 control of GCB formation, plasma cell differentiation, and IgG1 antibody production, as demonstrated by restoration of plasma cell differentiation of *Dhx29*-deficient B cells by ectopic expression of E12 and E47, two isoforms of TCF3.

This study also revealed a previously unrecognized role of TLE3 in controlling plasma cell differentiation. TLE3 is a member of the transducing-like enhancer of split family proteins (TLEs)/Groucho family of transcriptional cofactors. Those proteins do not have a DNA-binding domain but interact with multiple transcription factors and function as integrators of transcription factor complexes acting downstream of various external cues (Ren et al, 1999). A recent study showed that TLE3 functions as a transcriptional coactivator to increase chromatin opening at CD8^+^ effector memory cell-characteristic sites and to activate transcription of CD8^+^ effector memory cell signature genes (Zhao et al, 2024). Another study reported that TLE3 cooperates with Hhex to regulate the transcriptional circuitry governing GCB cell differentiation into memory B cells (MBCs)(Laidlaw et al, 2020). In this study, we showed that TLE3 associates with the promoter region of the *Prdm1* gene and promotes its transcription. Future investigations are warranted to elucidate molecular mechanisms underlying TLE3 control of *Prdm1* transcription.

## Methods

**Table:** Reagents and tools table

Reagent/resource	Reference or source	Identifier or catalog number
**Experimental models**		
*Rosa26*^Cas9-GFP^ mice	Jackson Lab	Jax stock #024858
Mb1-Cre mice	Jackson Lab	Jax stock #020505
CD19-Cre mice	Jackson Lab	Jax stock #006785
Cγ1-Cre mice	Jackson Lab	Jax stock #010611
CD45.1 mice	Jackson Lab	Jax stock #002014
Rag1^-/-^ mice	Jackson Lab	Jax stock #034159
*Rosa26*^LSL-YFP^ mice	Jackson Lab	Jax stock #006148
40LB cells	Nojima et al, 2011 Gift from Daisuke Kitamura lab	
HEK293T cells	Gift from Jiahuai Han lab	
**Recombinant DNA**		
MSCV-pU6-(BbsI)-CcdB-(BbsI)-Pgk-Puro-T2A-BFP	Addgene	#86457
pCL-Eco	Addgene	#12371
RV-EF1a-3HA-IRES-GFP	This paper	N/A
RV-EF1a-5’UTR-Renilla-HSV-TK-Firefly	This paper	N/A
RV-EF1a-Renilla-Binding region-Firefly	This paper	N/A
Pet28a-6His	This paper	N/A
**Antibodies**		
Anti-CD3	eBioscience	Clone 145-2C11
Anti-CD4	eBioscience	Clone GK1.5
Anti-CD8	eBioscience	Clone 53-6.7
Anti-CD19	eBioscience	Clone eBio1D3
Anti-CD38	eBioscience	Clone 90
Anti-IgM	eBioscience	Clone II/41
Anti-IgD	eBioscience	Clone IA6-2
Anti-BP1	eBioscience	Clone 6C3
Anti-CD24	BD	Clone M1/69
Anti-CD44	BD	Clone IM7
Anti-CD95	BD	Clone Jo2
Anti-IgG1	BD	Clone A85-1
Anti-CD138	BD	Clone 281-2
Anti-CD21	Biolegend	Clone 7E9
Anti-CD23	Biolegend	Clone B3B4
Anti-CD93	Biolegend	Clone AA4.1
Anti-CD43	Biolegend	Clone S11
Anti-CD117	Biolegend	Clone 2B8
Anti-GL7	Biolegend	Clone GL7
Anti-IgM	Biolegend	Clone RMM-1
Anti-IgE	Biolegend	Clone RME-1
Anti-B220	Biolegend	Clone RA3-6B2
Anti-CD45.1	Biolegend	Clone A20
Anti-HA	Santa Cruz	Catalog sc-7392
Anti-TCF3	Santa Cruz	Catalog sc-416
Anti-BLIMP1	Santa Cruz	Catalog sc-47732
Anti-DHX29	Proteintech	Catalog 13923-1-AP
Anti-IRF4	Proteintech	Catalog 11247-2-AP
Anti-TLE3	Proteintech	Catalog 11372-1-AP
Anti-Lamin B1	Proteintech	Catalog 66095-1-Ig
Anti-β-tubulin	Proteintech	Catalog 66240-1-Ig
Anti-DHX29	Cell Signaling Technology	Catalog 4648S
Anti-BCL6	Cell Signaling Technology	Catalog 4242S
Anti-PAX5	Cell Signaling Technology	Catalog 8970S
Anti-BiP	Cell Signaling Technology	Catalog 3177S
Anti-c-Myc	Cell Signaling Technology	Catalog 5605S
Anti-BACH2	Abcam	Catalog ab243148
Anti-β-actin	ABclonal	Catalog AC026
HRP-conjugated goat anti-rabbit IgG (H + L)	Abclonal	Catalog AS014
HRP-conjugated goat anti-mouse IgG (H + L)	Abclonal	Catalog AS003
Biotin anti-H-2Kd	Biolegend	Catalog 116604
Biotin anti-CD5	Biolegend	Catalog 100604
Biotin anti-CD9	BD	Catalog 558749
Biotin anti-CD43	Biolegend	Catalog 121204
Biotin anti-CD93	eBioscience	Catalog 13-5892-85
Biotin anti-Ter119	Biolegend	Catalog 116204
Biotin anti-IgG1	SouthernBiotech	Catalog 1070-08
Biotin anti-IgM	SouthernBiotech	Catalog 1020-08
**Oligonucleotides and other sequence-based reagents**		
sgRNAs	This study	Dataset EV1
Primers	This study	Table EV2
**Chemicals, enzymes, and other reagents**		
BeaverBeads Streptavidin	BEAVER	Catalog 22307
RPMI 1640	Gibco	Catalog 31800089
DMEM	Gibco	Catalog 12800082
FBS	ExCell	Catalog FSP500
NEAA	Gibco	Catalog 11140050
β-mercaptoethanol	Sigma	Catalog M3148
Penicillin/streptomycin	Gibco	Catalog 15140122
IL-4	Novoprotein	Catalog CK74
IL-21	Novoprotein	Catalog CK10
Polybrene	Sigma	Catalog TR-1003-G
Fixation/Permeabilization buffer	eBioscience	Catalog 00-5523-00
Cell Trace Violet	Thermo Scientific	Catalog C34557
PE Annexin V apoptosis detection kit	BD	Catalog 559763
BeyoClick™ EdU Cell Proliferation Kit with AF647	Beyotime	Catalog C0081S
Ovalbumin (257-264) chicken≥97% (HPLC)	Sigma	Catalog S7951
Imject™ Alum Adjuvant	Thermo Scientific	Catalog 77161
NP-OVA	Biosearch Technologies	Catalog N-5051-100
Lipopolysaccharides from Escherichia coli O55: B5	Sigma	Catalog L5418
Av-HRP	Vector Laboratories	Catalog A-2004
AEC substrate	Vector Laboratories	Catalog SK-4200
BD Fix/Perm Solution	BD	Catalog 51-2090KZ
Sucrose	Sigma	Catalog S0389
Tissue-Tek OCT	Sakura	Catalog 4583
AG RNAex Pro reagent	Accurate Biology	Catalog AG21102
TRIzol™ LS Reagent	Thermo Scientific	Catalog 10296028
HiScript III RT SuperMix for qPCR	Vazyme	R323-01
ChamQ Universal SYBR qPCR Master Mix	Vazyme	Q711-02
SDS loading buffer	BIO-RAD	1610747
Immobilon Western Chemiluminescent HRP Substrate	Merck Millipore	Catalog WBKLS0500
Rneasy® Mini Kit (50)	QIAGEN	Catalog 74104
EasyPep^TM^ MS kit	Thermo Scientific	Catalog A45733
Cycloheximide	Cell Signaling Technology	Catalog 2112S
SUPERase·In RNase inhibitor	Invitrogen	Catalog AM2696
Microspin™ S-400 HR	GE Healthcare	Catalog 27-5140-01
RNA Clean and Concentrator-25 kit	Zymo Research	Catalog R1017
VAHTS RNA Clean Beads	Vazyme	Catalog N412
Hyperactive Universal CUT&Tag Assay Kit	Vazyma	Catalog TD903
TruePrep Index Kit V2	Vazyme	Catalog TD202
VAHTS DNA Clean Beads	Vazyme	Catalog N411
Ethanol	Sigma	Catalog E7023
Hyperactive ATAC-Seq Library Prep Kit for Illumina	Vazyme	Catalog TD711
The Dual Luciferase Reporter Assay System	Promega	Catalog E1960
Protein A Dynabeads	Thermo Scientific	Catalog 10001D
Proteinase Inhibitor Cocktail	Thermo Scientific	Catalog 87786
Biotin RNA labeling mix	Roche	Catalog 11685597910
T7 high yield RNA transcription kit	Vazyme	Catalog DD4201
Pierce^TM^ Magnetic RNA-Protein Pull-Down Kit	Thermo Scientific	Catalog 20164
LightShift® Chemiluminescent RNA EMSA Kit	Thermo Scientific	Catalog 20158
Pierce IP lysis buffer	Thermo Scientific	Catalog 87787
Ni-NTA Agarose	QIAGEN	Catalog 1018244
**Software**		
FlowJo software	Treestar	
Imaris software	Oxford Instruments	
Thermo Proteome Discoverer (v2.5.0.400)	https://www.thermofisher.cn/cn/zh/home/industrial/mass-spectrometry/liquid-chromatography-mass-spectrometry-lc-ms/lc-ms-software/multi-omics-data-analysis/proteome-discoverer-software.html	
HISAT2 (v2.1.0)	Kim et al, 2015	
FeatureCounts (v2.0.1)	Liao et al, 2014	
Gene Ontology (GO)	clusterProfiler R package https://www.geneontology.org/	
GSEA (v4.1.0)	Subramanian et al, 2005 https://www.gsea-msigdb.org/gsea/index.jsp	
RiboCode (v1.2.13)	Xiao et al, 2018	
STAR (v2.7.10b)	Dobin et al, 2013	
deltaTE	Chothani et al, 2019	
FastQC (v0.11.9)	http://www.bioinformatics.babraham.ac.uk/projects/fastqc/	
Bowtie2 (v2.3.5.1)	Langmead and Salzberg, 2012 https://bowtie-bio.sourceforge.net/bowtie2/index.shtml	
Samtools (v1.18)	Li et al, 2009	
Deeptools (v2.0)	Ramirez et al, 2016	
MACS2 (v2.2.8)	Zhang et al, 2008	
Integrative Genomics Viewer (IGV) (v2.18.4)	Robinson et al, 2011	
**Others**		
LSRFortessa X-20	BD	
Quanteon	ACEA Biosciences	
BD Aria FUSION	BD	
Amersham Imager 600	GE Healthcare	
Leica TCS SP8 confocal microscope	Leica	
BIOCOMP 152-002	BIOCOMP	
BIO RAD CFX96	BIO RAD	
BIO RAD CFX384	BIO RAD	
Illumina HiSeq	Illumina	
Illumina NovaSeq platform	Illumina	

### Methods and protocols

#### Mice

*Dhx29*^fl/fl^ mice on the C57BL/7J background were generated by Xiamen University Laboratory Animal Center with the method of haploid embryonic stem cells (Zhong et al, 2015). Briefly, ribonucleoproteins (RNPs) consisting of Cas9 protein, sgRNA pairs targeting *Dhx29*, and loxP-flanked DNA template were injected into haploid embryonic stem cells. Haploid embryonic stem cells carrying the desired genetic modification were identified by PCR and injected into oocytes, followed by in vitro culture and embryo transfer into pseudo-pregnant female mice, whose offspring were screened for desired genetic modifications. *Rosa26*^Cas9-GFP^(Jax stock #024858) (Platt et al, 2014), Mb1-Cre (Jax stock #020505) (Hobeika et al, 2006), CD19-Cre (Jax stock #006785)(Rickert et al, 1997), Cγ1-Cre(Jax stock #010611) (Casola et al, 2006), CD45.1 (Jax stock #002014) (Janowska-Wieczorek et al, 2001; Schluns et al, 2002; Shen et al, 1985; Yang et al, 2002), *Rag1*^−/−^ (Jax stock #034159), *Rosa26*^LSL-YFP^ (Jax stock #006148) (Srinivas et al, 2001) were previously reported. B-cell development was analyzed at the age of 6-8 weeks. Mice were immunized and bled to measure immunoglobulin concentrations at the age of 8–12 weeks. All mice were bred and housed under specific pathogen-free conditions under a 12-h light–dark cycle. All animal experiments were approved by the Animal Care and Use Committee of Xiamen University.

#### Purification of naïve B cells

Single cell suspension of splenocytes was prepared following red blood cell lysis with ACK lysis buffer (150 mM NH_4_Cl, 10 mM KHCO_3_, 0.1 mM EDTA) and stained with biotin-conjugated antibodies against CD5, CD9, CD43, CD93, and Ter119 (eBioscience and Biolegend) for 10 min at 4 °C. Cells were washed with MACS buffer (PBS plus 2% FBS and 2 mM EDTA), and incubated with BeaverBeads Streptavidin (1 μM) (BEAVER) for 10 min at 4 °C. After incubation with BeaverBeads Streptavidin, B cells were negatively selected in a magnetic field and resuspended in B-cell medium.

#### Cell culture

B cells were cultured in B-cell medium, which includes RMPI-1640 (Gibco) supplemented with 10% FBS (ExCell), 1× NEAA (Gibco), 50 μM β-mercaptoethanol (Sigma), 100 U/mL penicillin and streptomycin (Gibco). HEK293T cells were maintained in DMEM (Gibco) complete medium with 10% FBS (ExCell), 1× NEAA (Gibco), and 100 U/mL penicillin and streptomycin (Gibco). NIH3T3-derived 40LB feeder cells expressing CD40L and BAFF were maintained in DMEM complete medium and were irradiated with 120 Gy before use. For iGCB culture, the culture medium of 40LB cells was aspirated following irradiation, and 8 × 10^4^ naïve B cells were plated with 10 mL B cell medium plus 1 ng/mL IL-4 (Novoprotein) to induce differentiation into iGCB cells. For iPC culture, 5 × 10^4^ iGCB cells were replated with freshly irradiated 40LB cells with 10 mL B cell medium plus 10 ng/mL IL-21 (Novoprotein) to induce differentiation into plasma cells (iPCs) for another 4 days in six-well plates.

#### Plasmids

For sgRNA expression, DNA oligonucleotides were cloned into a MSCV-pU6-(BbsI)-CcdB-(BbsI)-Pgk-Puro-T2A-BFP vector by T4 ligase-dependent ligation. The sequences of sgRNA oligos were listed in Dataset EV1. Coding sequences of *Dhx29*, *Tcf3*, and *Tle3* were amplified from mouse B cell cDNA library and inserted into a RV-EF1a-3HAtag-IRES-GFP vector by ligase-independent cloning. Coding sequences of *Dhx29* and *Dhx29*-RBDmut were inserted into a Pet28a-6His vector by ligase-independent cloning.

#### Retroviral transduction

For virus production, 293T cells were plated at 1.5 × 10^6^ cells per well in six-well plates 1 day before transfection to reach ~80% confluency on the day of transfection. Each well was transfected with 3 µg retroviral vector and 1 µg packaging plasmid (pCl-Eco, Addgene) by the calcium phosphate method. Medium was replaced with a fresh one 8–12 h after transfection. Retroviral supernatants were collected at 36–48 h after transfection, and cell debris was removed by centrifugation at 800× *g* for 3 min. For retroviral transduction, naïve B cells were cultured in the iGCB system as described above. On day 2.5 of culture, 9 mL B cell medium from each well of 6-well plates was collected and kept in 37 °C water bath. 1 mL retroviral supernatant with 10 ng/mL polybrene (Sigma) was added to B cells. Spinoculation was performed by centrifugation at 1500× *g* for 1 h at 34 °C and incubation at 37 °C for 4 h. The previously collected B-cell medium was added back to cells, followed by culture until analysis.

#### FACS analysis and sorting

Single cell suspension was freshly prepared from the spleen, draining lymph nodes, and bone marrow, depleted of red blood cells by ACK lysis, stained with antibodies for surface markers in FACS buffer (PBS with 0.5% BSA), and incubated at 4 °C for 20 min in the dark, washed, and resuspended in FACS buffer. After surface marker staining, cells were fixed with 4% PFA at room temperature for 15 min. A Foxp3 staining kit was used for staining intracellular IgG1 and IgE following the manufacturer’s instructions (eBioscience). For cell proliferation analysis, cells were labeled with Cell Trace Violet (Thermo Scientific) at room temperature for 10 min following the manufacturer’s instructions. A PE Annexin V apoptosis detection kit (BD Biosciences) was used for cell death detection. Cell cycle staining was performed following the instructions of the BeyoClick™ EdU Cell Proliferation Kit with an Alexa Fluor 647 Kit (Beyotime). For cell sorting, B cells were stained with anti-CD19, sorted into 15-mL tubes containing serum, and pelleted by centrifugation for immunoblot analysis. All flow cytometry data were acquired on LSRFortessa X-20 (BD), Quanteon (ACEA Biosciences, Agilent), and analyzed with FlowJo software (Treestar).

#### Immunization and ELISA

For OVA/alum immunization, solutions I (50 mg OVA in 25 mL PBS) and II (25 mL 10% KAl(SO_4_)_2_ in PBS) were mixed. pH was adjusted to 6.5–7.0 to precipitate OVA/alum. The mixture was aliquoted and frozen at −80 °C. Each mouse was immunized with 200 μL OVA/alum mixture by intraperitoneal injection. In all, 7.5 days post immunization, splenocytes were harvested for flow cytometry analysis. For NP-OVA immunization, each mouse was injected intraperitoneally with 50 μg of NP-OVA (Biosearch Technologies) precipitated in alum. 14 days after immunization, NP-specific antibody-secreting cells (ASCs) were measured by ELISpot assay. For antibody responses, each mouse was injected intraperitoneally with 50 μg NP-OVA/alum. Serum samples were collected at 0, 7, 14, and 21 days. Concentrations of NP-specific IgM (Southern Biotech) and IgG1 (Southern Biotech) were determined by ELISA. ELISA for NP-specific antibody was measured as previously reported (Kang et al, 2013). For NP-OVA/alum/LPS immunization, each mouse was injected intraperitoneally with 100 μg of NP-OVA (Biosearch Technologies) precipitated in alum and with 20 μg LPS (Sigma). Ten days post immunization, splenocytes were harvested for flow cytometry analysis.

#### ELISpot assay

NP-specific IgG1 ASCs were detected by ELISpot assay in a MultiScreen 96-well filtration plate (Millipore, MSIPS4W10) coated with 10 μg/mL NP_29_-BSA (SouthernBiotech). Cells were serially diluted and added to individual wells in triplicates, followed by incubation for 3 h at 37 °C with 5% CO_2_. Anti-NP IgG1 spots were detected by biotin-conjugated anti-mouse IgG1 antibody (SouthernBiotech) in combination with Av-HRP and AEC substrate (Vector Laboratories).

#### Immunohistochemistry

Spleens from immunized mice were fixed in Fix/Perm Solution (BD) at 4 °C overnight. The fixative solution was discarded, and fixed spleens were washed with PBS for 10 min on a shaker. 30% sucrose (Sigma) was added to tissues at 4 °C, followed by incubation overnight and embedding in OCT (Sakura). Tissue sections were cut with the Leica CM1950 machine at a set of 10 μm. Sections were immunostained for IgD, CD3, and GL7 at room temperature in a dark box for 24 h. Images were acquired by using a Leica TCS SP8 confocal microscope (Leica) and analyzed by Imaris software.

#### Bone marrow reconstitution

To generate chimeras with normal B cells, bone marrow cells from *Cγ1*^Cre^ control or *Dhx29*^fl/fl^*Cγ1*^Cre^ mice (CD45.2^+^) were mixed with bone marrow cells from wild-type CD45.1 mice (CD45.1^+^) with a 50:50 ratio and adoptively transferred into lethally irradiated (4.5 Gray) Rag1^−/−^ recipient mice. In this experiment, a total of 5 million bone marrow cells were transferred into each recipient. Twelve weeks after reconstitution, recipient mice were immunized with OVA/alum and analyzed by flow cytometry at day 7.5 post immunization.

#### RNA extraction and RT-PCR

In total, 1–2 × 10^6^ B cells were lysed with TRIzol reagent. Total RNA was extracted following the manufacturer’s instructions and quantified by NanoDrop2000 (Thermo Scientific). HiScript III RT SuperMix for qPCR (Vazyme) was used for reverse transcription. Quantitative real-time PCR was performed with ChamQ Universal SYBR qPCR Master Mix (Vazyme). All RT-PCR data were acquired on BIO RAD CFX96 (BIO RAD). Primers for RT-PCR were listed in Table EV2.

#### Immunoblot

For immunoblot, B cells and iPCs were lysed in the lysis buffer (20 mM Tris-HCl pH 7.5, 1 mM EDTA, 1 mM EGTA, 1% TritonX-100, 2.5 mM sodium pyrophosphate, 150 mM NaCl, 1 mM Na_3_VO_4_, and 1% SDS) supplemented with Halt Protease & Phosphatase Inhibitor Cocktail on ice for 30 min. SDS loading buffer (BIO-RAD) was added to cell lysates, followed by boiling at 100 °C for 10 min before immunoblot analysis. Cell lysates were resolved on SDS-PAGE gels and transferred to PVDF membranes (Merck Millipore). Primary antibodies were diluted in NCM Universal Antibody Diluent (NCM biotech), followed by overnight incubation with the membranes at 4 °C. After washing three times in TBS buffer with 0.5% Tween 20, horseradish peroxidase (HRP)-conjugated goat anti-rabbit or goat anti-mouse antibody (Abclonal) was incubated with the membrane for 1 h. After washing 3 times in TBS buffer with 0.1% Tween 20, protein bands were visualized with Immobilon Western Chemiluminescent HRP Substrate (Merck Millipore) following the manufacturer’s instructions (GE Healthcare). Images were acquired with Amersham Imager 600 (GE Healthcare).

#### RNA-seq and data analysis

*Dhx29*^fl/fl^ and *Dhx29*^fl/fl^*Cγ1*^Cre^ day 4 iGCB cells (iPC 0 h) or iGCB cells cultured with fresh 40LB cells plus IL-21 for 12 (iPC 12 h) or 24 h (iPC 24 h) were collected after FACS sorting. Total RNA from iPC 0, 12, and 24 h samples was isolated using the RNAeasy Kit (QIAGEN) following the manufacturer’s instructions. After total RNA isolation, sequencing libraries were constructed with an NEB Next® Ultra^TM^ RNA Library Prep Kit from Illumina® (NEB) following the manufacturer’s instructions. The clustering of index-coded samples was performed on a cBot Cluster Generation System via the TruSeq PE Cluster Kit v3-cBot-HS (Illumina) following the manufacturer’s instructions. After cluster generation, the library preparations were used for sequencing on an Illumina NovaSeq platform (Novogene). Quality assessment of sequencing data is provided in Appendix Figs. S5 and S6. The raw fastq files were treated with Trim Galore (v0.6.7) to remove adapter sequences and low-quality reads. Filtered reads were aligned to the mouse reference genome (*mm10*) by HISAT2 (v2.1.0) with default parameters (Kim et al, 2015). Raw counts were generated by featureCounts (v2.0.1) (Liao et al, 2014), normalized, and analyzed by DESeq2 in R. Differential expression analysis was performed with *P* value of 0.05. Gene Ontology (GO) enrichment of differentially expressed genes was conducted by the clusterProfiler R package with corrected *P* value < 0.05 (Yu et al, 2012). Differentially expressed genes were analyzed with GSEA (v4.1.0) to find enriched biological processes and signaling pathways (Subramanian et al, 2005). Heatmaps and volcano plots were visualized using the R package.

#### LC-MS/MS and data analysis

In all, 2 × 10^6^ *Dhx29*^fl/fl^ and *Dhx29*^fl/fl^*Cγ1*^Cre^ day 3.5 iGCB cells were harvested and processed for the EasyPep^TM^ MS kit (Thermo Scientific), which was used for TMT labeling following the manufacturer’s instructions. LC-MS/MS data were acquired on an Orbitrap Eclipse mass spectrometer coupled with a Vanquish Neo UPLC system. Data were searched and quantified utilizing the Thermo Proteome Discoverer (v2.5.0.400). Full MS and MS/MS spectra were searched against the Mus musculus Uniprot database (www.uniprot.org). The differential expression analysis was performed with Perseus software to determine protein expression changes between *Dhx29*^fl/fl^ and *Dhx29*^fl/fl^*Cγ1*^Cre^ cells. Quality assessment of sequencing data is provided in Appendix Figs. S5 and S6.

#### Ribosome profiling and data analysis

Day 3.5 iGCB cells were treated with cycloheximide (Cell Signaling Technology) at a final concentration of 100 μg/mL. Live B cells were sorted, snap frozen, and stored at −80 °C until further processing. Total RNA extraction from the same samples was performed with the RNeasy Kit (QIAGEN) in accordance with the manufacturer’s guidelines, followed by RNA-seq analysis. Ribosome profiling was conducted by Gene Denovo Biotechnology. Samples were lysed in lysis buffer and centrifuged at 20,000× *g* for 10 min at 4 °C. Supernatant was collected. In total, 10 µL RNase I (NEB) and 6 µL DNase I (NEB) were added to 400 µL lysate, followed by incubation for 45 min at room temperature with gentle mixing. Nuclease digestion was stopped by adding 10 µL SUPERase·In RNase inhibitor (Invitrogen). Size exclusion columns (GE Healthcare) were equilibrated with 3 mL of polysome buffer by gravity flow and centrifuged at 600× *g* for 4 min at room temperature. The digested products from the last step were added to the pre-equilibrated columns, which were centrifuged at 600× *g* for 2 min. In total, 10 μL of 10% (wt/vol) SDS was then added to eluate, and ribosome footprints greater than 17 nt were isolated using the RNA Clean and Concentrator-25 kit (Zymo Research). rRNA was removed using the previously reported method (Morlan et al, 2012). In brief, short (50–80 bases) antisense DNA probes complementary to rRNA sequences were added to solution containing ribosome footprints. RNase H (NEB) and DNase I (NEB) were added to digest rRNA and residual DNA probes. Finally, ribosome footprints were further purified using magnet beads following the manufacturer’s instructions (Vazyme). Ribo-seq libraries were constructed using NEB Next® Multiple Small RNA Library Prep Set for Illumina® (NEB). Briefly, adapters were added to both ends of ribosome footprints, followed by reverse transcription and PCR amplification. PCR products in the 140–160 bp size range were enriched to generate a cDNA library and sequenced using Illumina HiSeq.

Ribosome profiling data were analyzed following the RiboCode workflow (Xiao et al, 2018). Quality assessment of sequencing data is provided in Appendix Figs. S5 and S6. Adapters, low-quality reads, and reads aligned to rRNA were removed from the raw data. The remaining reads were aligned to the Mus musculus genome assembly GRCm38 (*mm10*) using STAR(v2.7.10b) (Dobin et al, 2013). The alignment files were then analyzed using RiboCode (v1.2.13) to identify translated ORFs. Read counts of ribosome footprints were generated using the ModifyHTseq script provided by RiboMiner (v0.2) (Li et al, 2020). Ribosome profiling data were integrated with matched RNA-seq data to reveal translational regulation. RNA-seq data obtained from the same samples were processed to read counts as described in the RNA-Seq session. Raw mRNA counts were integrated with RPF counts by deltaTE (Chothani et al, 2019) to calculate translation efficiency (TE).

#### CUT&Tag

The assay was performed following the manufacturer’s instructions of the Hyperactive In-Situ ChIP Library Prep Kit for Illumina (pA/G-Tn5) kit (Vazyme). Briefly, 1 × 10^5^ cultured cells were harvested and washed once with 500 μL Wash Buffer before they were bound to ConA beads for 10 min at room temperature. Cells were incubated overnight with anti-TLE3 antibody (Proteintech) and anti-rabbit IgG at 4 °C in antibody buffer, which contains 0.05% Digitonin. Cells were pelleted on a magnet and supernatant was removed, goat anti-rabbit IgG (Abcam) was added, followed by incubation for 1 h at room temperature. Then the cells were washed three times with Dig-wash Buffer and incubated with 0.05 μM Hyperactive pA/G-Tn5 Transposon for 1 h at room temperature. Cells were washed again three times with Dig 300 buffer, resuspended in tagmentation buffer, and incubated at 37 °C for 1 h. Tagmentation was stopped by adding proteinase K, buffer LB, and DNA extract beads. After incubation at 55 °C for 10 min, beads were pelleted on a magnet, and supernatant was removed. Beads were gently rinsed twice with 80% ethanol, and DNA was eluted with 20 μL nuclease free water. For library amplification, 15 μL of purified DNA was mixed with 25 μL of 2× CAM, along with 5 μL of uniquely barcoded i5 and i7 primers from the TruePrep Index Kit V2(Vazyme). To purify the PCR products, 2× volumes of VAHTS DNA Clean Beads (Vazyme) were added and incubated at room temperature for 15 min. Beads were gently rinsed twice with 80% ethanol and DNA was eluted with 20 μL nuclease free water. After quality inspection, 15 μL of eluted DNA libraries were sequenced on the Illumina PE150 platform to a depth of 15 G per sample by Majorbio Biotech, and the remaining samples were analyzed by PCR. PCR primers are listed in Table EV2.

#### ATAC-seq

The assay was performed following the manufacturer’s instructions of the Hyperactive ATAC-Seq Library Prep Kit for Illumina (Vazyme). Briefly, 1 × 10^5^ cultured cells were collected and processed for ATAC-sequencing. Cells were washed and lysed on ice in a tube containing 50 μL lysis buffer. Nuclei were collected and subjected to a transposition reaction in a preheated metal bath at 37 °C. DNA fragments were extracted and purified by ATAC DNA Extract Beads. Libraries were constructed using the TruePrep Index Kit V2(Vazyme) for sequencing. After quality inspection, the DNA libraries were sequenced on the Illumina PE150 platform to a depth of 10 G per sample by GENEWIZ.

#### CUT&Tag and ATAC-seq data analysis

For both CUT&Tag and ATAC-sequencing data analysis, raw paired-end sequenced reads were first cut for adapter sequences and trimmed using Trim Galore (v0.6.7). Sequencing quality was evaluated by FastQC (v0.11.9). The cleaned reads were aligned to the mouse reference genome *mm10* using Bowtie2(v2.3.5.1) (Langmead and Salzberg, 2012). Samtools(v1.18) was used to convert the SAM files into BAM format, filter out low-quality reads, sort, and fixmate reads (Li et al, 2009). Deeptools(v2.0) (Ramirez et al, 2016) was used for normalizing signals from BAM files via bamCoverage, and computeMatrix was used for calculating the overall signal distribution around the peak center called by MACS2 (v2.2.8) (Zhang et al, 2008). Duplicate reads were removed using Picard (v3.1.0). MACS2(v2.2.8) was used for peak calling and R package ChIPseeker (v1.36.0) was used for annotation. Data were visualized using plotHeatmap in Deeptools. Genome browser tracks were visualized with Integrative Genomics Viewer (IGV)(v2.18.4) (Robinson et al, 2011). Quality assessment of sequencing data is provided in Appendix Fig. S6.

#### Luciferase reporter assay

5’UTRs of *β-actin*, *Tcf3*, and *Tle3* were amplified from mouse B cell cDNA library and cloned into the RV-EF1a-5’UTR-Renilla-HSV-TK-Firefly vector by ligase-independent cloning. *Dhx29*^fl/fl^ and *Dhx29*^fl/fl^*Cγ1*^Cre^ B cells were cultured with 40LB cells and transduced with retroviruses encoding different 5’UTRs on day 2.5 of iGCB culture. In all, 2–3 × 10^6^ iGCB cells were used for each sample. Cells were collected at iGCB day 4, and luciferase activity was determined by the Dual Luciferase Reporter Assay System (Promega). Meanwhile, the TLE3 binding region of the *Prdm1* gene was amplified from mouse B cell genomic DNA and cloned into the RV-EF1a-Renilla-binding region-Firefly vector by ligase-independent cloning. Naïve B cells from *Rosa26*^Cas9-GFP^ mice were cultured with 40LB cells, transduced with 1:1 mix of retroviruses encoding NTC, Sg*Dhx29*, and Sg*Tle3* and retroviruses encoding luciferase reporters at day 2.5, and treated with puromycin (10 μg/mL) at day 3. Cells were harvested at iGCB day 5, and luciferase activity was determined by the Dual Luciferase Reporter Assay System (Promega).

#### Polysome profiling

Polysome profiling was performed as previously described (Jin et al, 2017). iGCB cells from one six-well plate were harvested with FACS buffer containing Cycloheximide (100 μg/mL), pelleted by centrifugation, and resuspended in 400 μL FACS buffer with Cycloheximide (100 μg/mL). To deplete 40LB cells, 3 μL biotin-conjugated anti-H-2Kd antibody (Biolegend) was added to the cell suspension and incubated with rotation at room temperature for 5 min. 20 μL BeaverBeads Streptavidin (1 μM) (BEAVER) was added to the mixture, followed by incubation at room temperature for 5 min. Beads were pelleted with a magnet. The supernatant, which contains iGCB cells, was collected and transferred to a new tube, followed by the addition of PBS containing Cycloheximide (100 μg/mL). iGCB cells were centrifuged at 600× *g* for 3 min, and the supernatant was discarded. Cell pellet was resuspended gently in 300 μL hypotonic buffer, followed by addition of an equal volume of hypotonic lysis buffer (2% sodium deoxycholate, 2% Triton X-100, 2.5 mM DTT, 10 units of RNase Inhibitors/mL, and 100 μg/mL CHX) and incubation on the ice for 20 min. The supernatant was collected as cytosolic fractions after centrifugation at 13,000× *g* for 10 min. Cytosolic fractions were loaded onto a 10–50% sucrose gradient containing RNase inhibitor and centrifuged at 38,000 rpm for 2 h using a Beckman SW41 rotor. The sucrose gradient was separated into 20 fractions from the top, and absorbance at 260 nm was monitored continuously by a Piston Gradient Fractionator (Biocomp). 1 μL 80 pg/uL exogenous RNA control (custom synthesized, full-length sequence: ACUUGCAAAGCCAAUUCCCGAAGAUCGUCUCAAUCGCACAGGAUCCCAAGCUUGAAUUCAUGUUAUAACACUUCUCUCAUGCCUGAAACUAAUGUCCUACUGUUCACUACAAUGG) was added to 150 μL samples aliquoted from each fraction, followed by extracting total RNA with TRIzol™ LS Reagent (Thermo Scientific) following the manufacturer’s instructions. Reverse transcription was performed with an equal amount of RNA samples using HiScript III RT SuperMix for qPCR (Vazyme) following the manufacturer’s instructions. Indicated mRNA was amplified and measured by ChamQ Universal SYBR qPCR Master Mix (Vazyme) for genes of interest. CT values were normalized to the spike-in exogenous RNA control. The ratio of the total in each fraction was calculated.

#### RNA immunoprecipitation

RNA immunoprecipitation (RIP) was performed following the immunoprecipitation procedure. *Dhx29*^fl/fl^*Cg1*^Cre^ B cells were transduced with retroviruses encoding empty vector, HA-DHX29, and HA-DHX29-RBDmut. In all, 1 × 10^7^ iGCB cells were used for each sample. In brief, anti-HA antibody (Santa Cruz) was incubated with Protein A Dynabeads (Thermo Scientific) for 8–12 h at 4 °C. When the incubation of the antibody and beads was nearly completed, cell samples were lysed in ice-cold lysis buffer supplemented with Proteinase Inhibitor Cocktail (Thermo Scientific) and SUPERase·In RNase inhibitor (Invitrogen), sonicated using Bioruptor plus (DIAGENODE), and centrifuged to remove nuclei. Supernatants were transferred to new tubes, followed by adding pre-washed antibody-bead slurry and rotating overnight at 4 °C. Before adding the antibody-bead slurry, 60 μL of each supernatant was removed and stored as input. After overnight incubation, beads were washed three times with high-salt buffer and twice with wash buffer. Beads were resuspended in 100 μL wash buffer, and 80 μL was transferred to a new tube for total RNA extraction and qPCR quantification. The remaining beads were mixed with SDS loading buffer (BIO-RAD) and boiled at 100 °C with for 10 min for immunoblot analysis.

#### In vitro transcription

The DNA templates were PCR amplified from a mouse B cell cDNA library with a T7 promoter and purified by DNA Clean Beads (Vazyme). For biotin-labeled RNA synthesis, biotin RNA labeling mix (Roche) was used. RNAs were synthesized using T7 high yield RNA transcription kit (Vazyme) and products were purified using TRIzol reagent (Accurate Biology).

#### RNA pull-down assay

The assay was performed following the manufacturer’s instructions of the Pierce^TM^ Magnetic RNA-Protein Pull-Down Kit (Thermo Scientific). 50 μL streptavidin magnetic beads were used per sample. Beads were washed twice with 20 mM Tris-HCl pH 7.5, resuspended in 1 × RNA capture buffer, and incubated with 50 pmol of biotin-labeled 5’UTRs of *Tcf3* and *Tle3* for 1 h at room temperature. 8 × 10^6^ iGCB cells were transduced with DHX29 or DHX29-RBDmut and lysed in Pierce IP lysis buffer (Thermo Scientific) with Proteinase Inhibitor Cocktail (Thermo Scientific) and SUPERase·In RNase inhibitor (Invitrogen) during the bead incubation time. Before adding the beads-RNA slurry, 45 μL of each supernatant was removed and stored as input. After incubation, the beads-RNA slurry was washed twice with 20 mM Tris-HCl pH 7.5, and resuspended in 1 × protein-RNA-binding buffer with 50% glycerol. Cell lysates were added to the beads-RNA slurry, followed by incubation at 4 °C with rotation for 4 h. Beads were washed four times with 1 × wash buffer. In total, 50 μL 1 × SDS-loading buffer was added to the washed beads, followed by boiling at 100 °C for 10 min and immunoblot analysis of proteins pulled down by RNA.

#### Protein purification

Coding sequences of *Dhx29* and *Dhx29*-RBDmut were inserted into a Pet28a-6His vector by ligase-independent cloning. DHX29-His and DHX29-RBDmut-His proteins were expressed in *Escherichia coli* BL21. Protein expression was induced with IPTG (1 mM) in 100 mL of bacterial culture at 20 °C for 24 h. The cultured BL21 cells were collected and sonicated in 4 mL lysis buffer (20 mM HEPES pH 7.4, 300 mM NaCl, 20 mM imidazole, 5% glycerin, 1 mM DTT) with protease inhibitors (Roche). Insoluble materials were removed by centrifugation, and the extracts were transferred to new tubes. 50 μL Ni-NTA agarose (QIAGEN) was added to 1 mL of the extract and the mixture was incubated with 0.4 mL 10% DDM (n-dodecyl-β-D-maltoside) at 4 °C for 2 h. After incubation, the resin was washed four times with wash buffer (20 mM HEPES pH 7.4, 200 mM NaCl, 20 mM imidazole, 5% glycerin, 1 mM DTT, 0.05% DDM) containing protease inhibitors. Protein was eluted with elution buffer (20 mM HEPES pH 7.4, 150 mM NaCl, 300 mM imidazole, 5% glycerin, 1 mM DTT, 0.05% DDM) containing protease inhibitors. The elution from Pet28a-6His empty vector obtained during protein purification was used as a negative control.

#### Electrophoretic mobility shift assay (EMSA)

Biotin-labeled RNA corresponding to the 5’UTRs of *Tcf3* or *Tle3* was incubated with purified DHX29, DHX29-RBDmut, or control eluate in binding buffer (150 mM KCl, 25 mM Tris-HCl, pH 7.4, 5 mM EDTA, 0.5 mM DTT, 5% glycerol, and 100 U/mL RNase inhibitor) at 30 °C for 30 min, followed by incubation on ice for 5 min. Native RNA loading buffer was then added, and samples were resolved on a native TBE gel. After electrophoresis, the gel was transferred to a nylon membrane and UV-crosslinked. Membranes were incubated with streptavidin-HRP, and signals were detected using an ECL chemiluminescent substrate. For competition-binding reactions, increasing concentrations of unlabeled *Tle3* or *Tcf3* 5’UTR RNA were added to the reaction mixture.

## Supplementary information

Appendix

Table EV1

Table EV2

Peer Review File

Dataset EV1

Source data Fig. 1

Source data Fig. 2

Source data Fig. 3

Source data Fig. 4

Source data Fig. 5

Source data Fig. 6

Source data Fig. 7

Source data Fig. 8

Expanded View Figures

## Data Availability

The datasets produced in this study are available in the following databases: RNA-Seq data: Gene Expression Omnibus GSE317658. Ribo-Seq data and paired RNA-seq data: Gene Expression Omnibus GSE317659.CUT&Tag data: Gene Expression Omnibus GSE317160. ATAC-seq data: Gene Expression Omnibus GSE317159. Mass spectrometry proteomics data: PRIDE PXD073591. The source data of this paper are collected in the following database record: biostudies:S-SCDT-10_1038-S44318-026-00805-0.
