# Supplementary material for: RNA helicase DHX29 controls the translation of transcription factors involved in germinal center response and plasma cell differentiation in mice
Source: EMBO J. 2026 May 26;45(13):4605–35. doi: 10.1038/s44318-026-00805-0 (PMC13324185; doi:10.1038/s44318-026-00805-0)
Supplement: Supplementary file 1 — Appendix [file 44318_2026_805_MOESM1_ESM.pdf]

Appendix for:

RNA helicase DHX29 controls the translation of transcription factors involved in germinal center response and plasma cell differentiation in mice

Contents

Appendix Figure S1……………………………………………………………...2

Appendix Figure S2…………………………………………………...………4

Appendix Figure S3……………………………………………………………...6

Appendix Figure S4……………………………………………………………...7

Appendix Figure S5……………………………………………………………...8

Appendix Figure S6……………………………………………………………...9

Appendix Figure S1

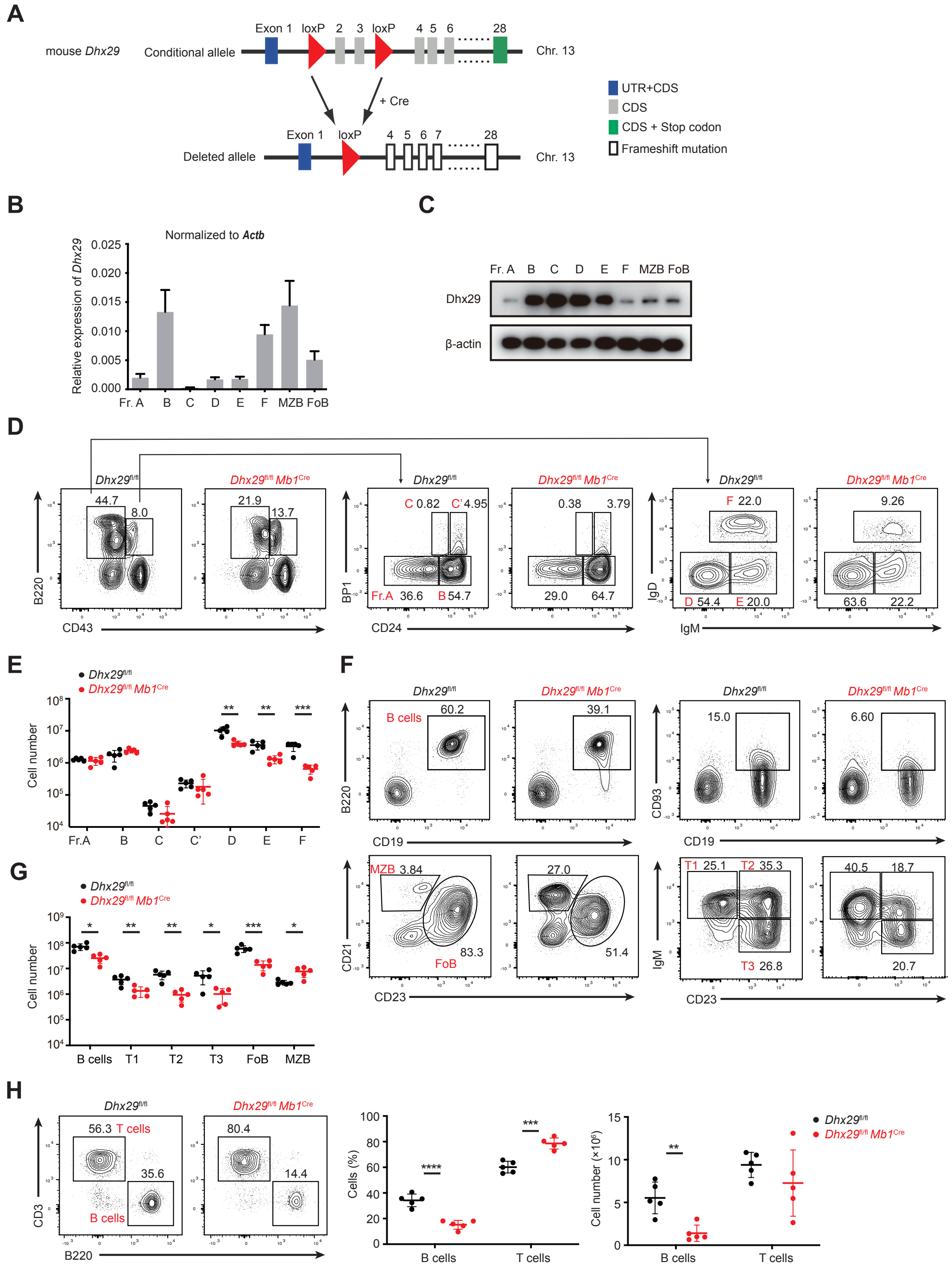

Appendix Figure S1. B cell development in *Dhx29*$^{fl/fl}$ and *Dhx29*$^{fl/fl}$*Mb1*^{Cre} mice

(**A**) Schematic representation of targeted Dhx29 alleles.

(**B, C**) RT–qPCR(**B**) and immunoblot (**C**) analysis of Dhx29 mRNA and protein expression during B cell development in the bone marrow and spleen. MZB, marginal zone B cells; FoB, follicular B cells.

(**D, E**) Flow cytometry analysis (**D**) and numbers (**E**) of B cell development in the bone marrow. Fr.A ($B220^{hi}CD43^{hi}CD24^{lo}BP1^{lo}$), Fr.B ($B220^{hi}CD43^{hi}CD24^{hi}BP1^{lo}$), Fr.C ($B220^{hi}CD43^{hi}CD24^{int}BP1^{hi}$), Fr.C'($B220^{hi}CD43^{hi}CD24^{hi}BP1^{hi}$), Fr.D ($B220^{hi}CD43^{lo}IgD^{lo}IgM^{lo}$), Fr.E ($B220^{hi}CD43^{lo}IgD^{lo}IgM^{hi}$), Fr.F ($B220^{hi}CD43^{lo}IgD^{hi}IgM^{hi}$). Each symbol represents an individual mouse. Fr.D: P= 0.0012; Fr.E: P= 0.0019; Fr.F: P= 0.0005.

(**F, G**) Flow cytometry analysis (**F**) and number (**G**) of immature B ($CD93^{hi}B220^{hi}$), transitional B (T1: $IgM^{hi}CD23^{lo}$, T2: $IgM^{hi}CD23^{hi}$, T3: $IgM^{lo}CD23^{hi}$), follicular B ($CD21^{hi}CD23^{hi}$) and marginal zone B ($CD21^{hi}CD23^{lo}$) cells in the spleen. Each symbol represents an individual mouse. B cells: P= 0.0179; T1: P= 0.0079; T2: P= 0.0011; T3: P= 0.0133; FoB: P= 0.0006; MZB: P= 0.0106.

(**H**) Flow cytometry analysis (left), percentage (middle), and numbers (right) of B and T cells in peripheral lymph nodes. Each symbol represents an individual mouse. B cells (%): P= 6.30E-06; T cells (%): P= 0.0001; B cells number ($\times 10^6$): P= 0.0033. Small horizontal lines indicate the mean (± SD.). Statistical significance was determined by unpaired, two-tailed student's t-test in (E, G and H). * $P < 0.05$, **$P < 0.01$, ***$P < 0.001$, ****$P < 0.0001$.

Appendix Figure S2

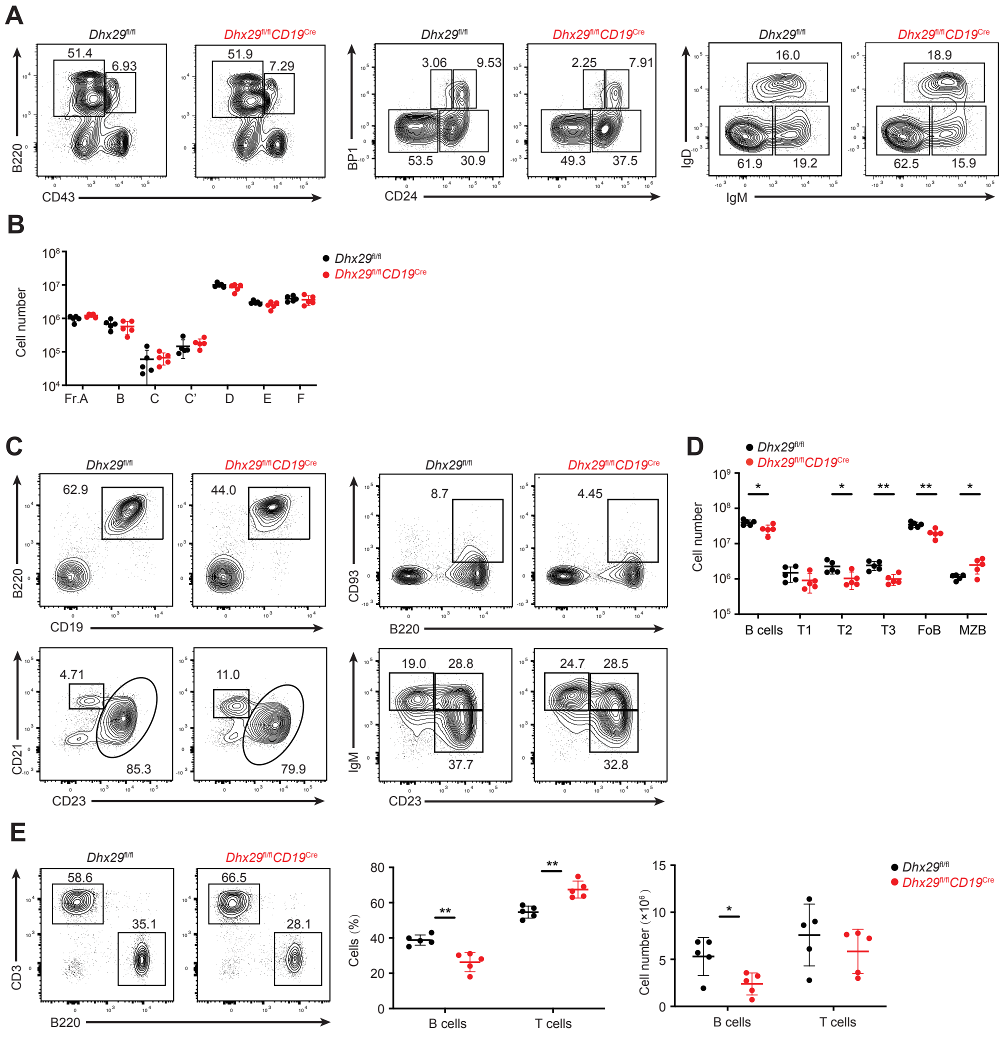

Appendix Figure S2. B cell development in *Dhx29*$^{fl/fl}$ and *Dhx29*$^{fl/fl}$*CD19*^{Cre} mice.

(**A, B**) Flow cytometry analysis (**A**) and numbers (**B**) of B cell development in the bone marrow. Fr.A (B220^{hi}CD43^{hi}CD24^{lo}BP1^{lo}), Fr.B (B220^{hi}CD43^{hi}CD24^{hi}BP1^{lo}), Fr.C (B220^{hi}CD43^{hi}CD24^{int}BP1^{hi}), Fr.C' (B220^{hi}CD43^{hi}CD24^{hi}BP1^{hi}), Fr.D (B220^{hi}CD43^{lo}IgD^{lo}IgM^{lo}), Fr.E (B220^{hi}CD43^{lo}IgD^{lo}IgM^{hi}), Fr.F (B220^{hi}CD43^{lo}IgD^{hi}IgM^{hi}).

(**C, D**) Flow cytometry analysis (**C**) and number (**D**) of immature B (CD93^{hi}B220^{hi}), transitional B (T1: IgM^{hi}CD23^{lo}, T2: IgM^{hi}CD23^{hi}, T3: IgM^{lo}CD23^{hi}), follicular B (CD21^{hi}CD23^{hi}) and marginal zone B (CD21^{hi}CD23^{lo}) cells in the spleen. B cells: P= 0.0152; T2: P= 0.0285; T3: P= 0.0031; FoB: P= 0.0034; MZB: P= 0.0279.

(**E**) Flow cytometry analysis (left), percentage (middle), and numbers (right) of B and T cells in peripheral lymph nodes. B cells (%): P= 0.0018; T cells (%): P= 0.0013; B cells number ($\times 10^6$):

P= 0. 0228. Each symbol represents an individual mouse. Small horizontal lines indicate the mean (± SD.). Statistical significance was determined by unpaired, two-tailed student's t-test in (D and E). *P < 0.05, **P < 0.01.

Appendix Figure S3

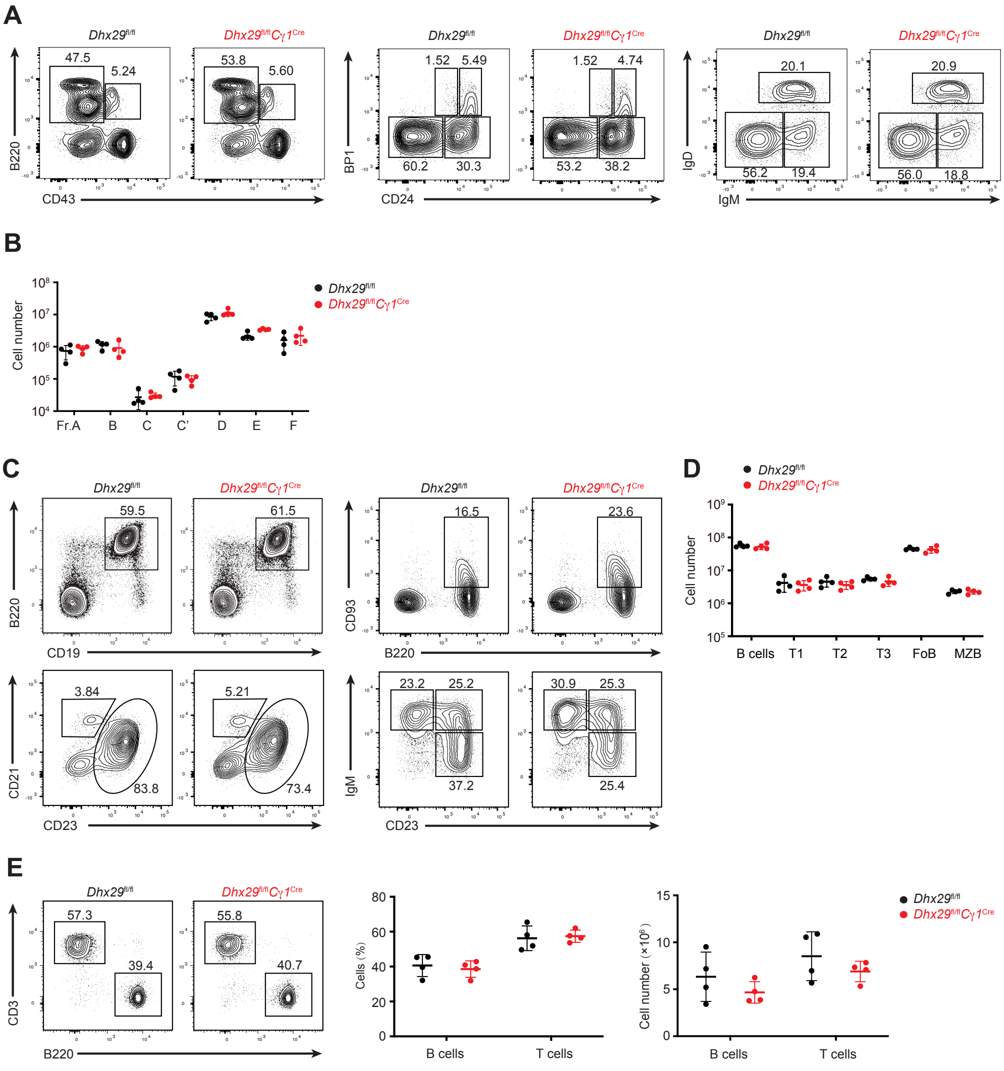

Appendix Figure S3. B cell development in *Dhx29*$^{fl/fl}$ and *Dhx29*$^{fl/fl}$*Cγ1*^{Cre} mice.

(**A, B**) Flow cytometry analysis (**a**) and numbers (**b**) of B cell development in the bone marrow. Fr.A ($B220^{hi}CD43^{hi}CD24^{lo}BP1^{lo}$), Fr.B ($B220^{hi}CD43^{hi}CD24^{hi}BP1^{lo}$), Fr.C ($B220^{hi}CD43^{hi}CD24^{int}BP1^{hi}$), Fr.C' ($B220^{hi}CD43^{hi}CD24^{hi}BP1^{hi}$), Fr.D ($B220^{hi}CD43^{lo}IgD^{lo}IgM^{lo}$), Fr.E ($B220^{hi}CD43^{lo}IgD^{lo}IgM^{hi}$), Fr.F ($B220^{hi}CD43^{lo}IgD^{hi}IgM^{hi}$).

(**C, D**) Flow cytometry analysis (**C**) and number (**D**) of immature B ($CD93^{hi}B220^{hi}$), transitional B (T1: $IgM^{hi}CD23^{lo}$, T2: $IgM^{hi}CD23^{hi}$, T3: $IgM^{lo}CD23^{hi}$), follicular B ($CD21^{hi}CD23^{hi}$) and marginal zone B ($CD21^{hi}CD23^{lo}$) cells in the spleen.

(**E**) Flow cytometry analysis (left), percentage (middle), and numbers (right) of T and B cells in peripheral lymph nodes. Each symbol represents an individual mouse. Small horizontal lines indicate the mean (± SD.).

Appendix Figure S4

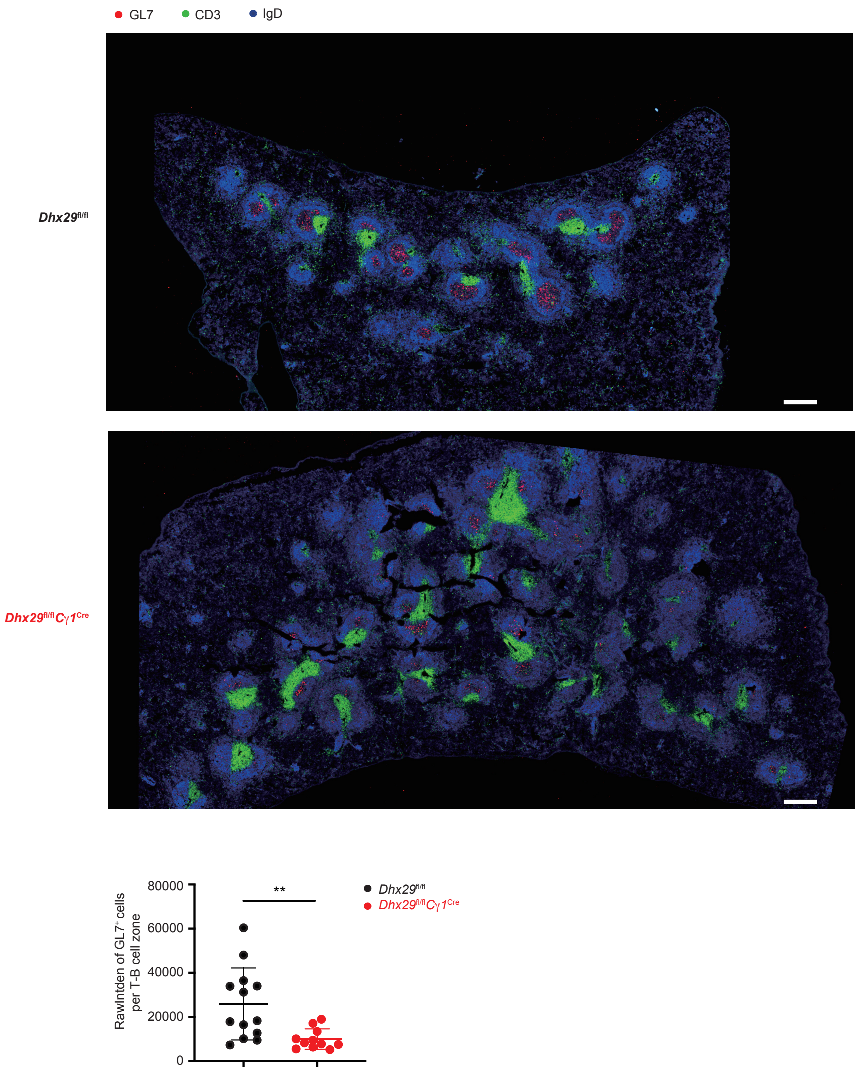

Appendix Figure S4. Germinal center formation in *Dhx29*$^{fl/fl}$ and *Dhx29*$^{fl/fl}$*Cγ1*^{Cre} mice. Immunohistochemistry staining of spleens of *Dhx29*$^{fl/fl}$ and *Dhx29*$^{fl/fl}$*Cγ1*^{Cre} mice. Red, GL7; green, CD3; blue, IgD. Scale bar, 300 μm. RawIntden of GL7^{+} B cells per T-B cell zone was quantified with Image J. Each symbol represents GL7^{+} B cells from a single T-B cell zone. Small horizontal lines indicate the mean (± SD.). Statistical significance was determined by unpaired, two-tailed student's t-test. P= 0.0050. **P < 0.01.

Appendix Figure S5

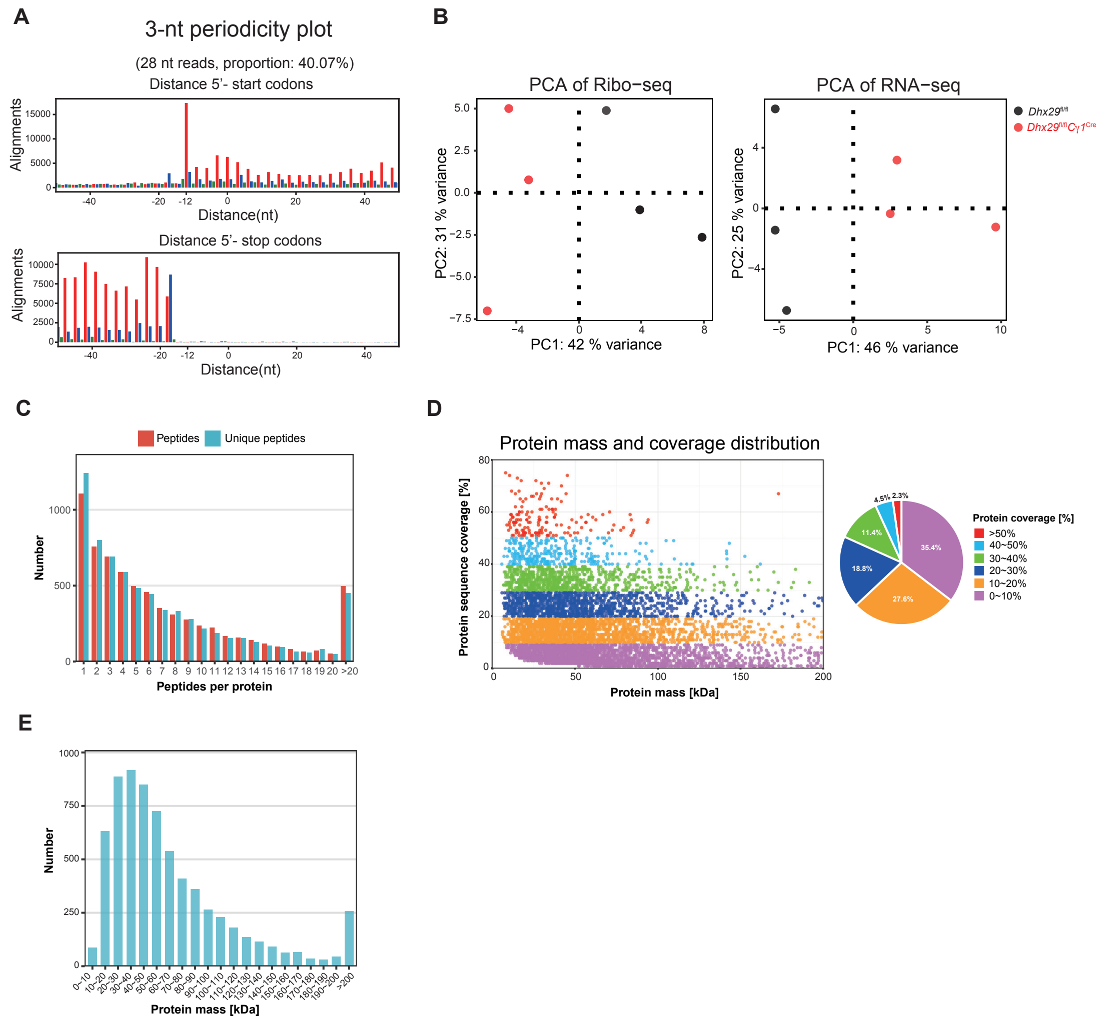

Appendix Figure S5. Data quality analysis of omics approaches used in Figure 6A.

(**A**) Ribosome profiling (Ribo-seq) data quality control: 3-nucleotide periodicity analysis by RiboCode.

(**B**) Comparative principal component analysis (PCA) of Ribo-seq and paired RNA-seq data. PCA plots compare the translatome (left) and transcriptome (right) of *Dhx29*$^{fl/fl}$ (black) and *Dhx29*$^{fl/fl}$*Cγ1*^{Cre} (red) iGCB day 3.5 cells.

(**C**) Distribution of peptides per protein in the mass spectrometry dataset. The X-axis represents the number of peptides detected for each protein, and the Y-axis represents the number of proteins. Red bars, peptides; blue bars, unique peptides.

(**D**) Protein mass and sequence coverage distribution in the mass spectrometry analysis.

(**E**) Mass distribution of identified proteins in the mass spectrometry analysis.

Appendix Figure S6

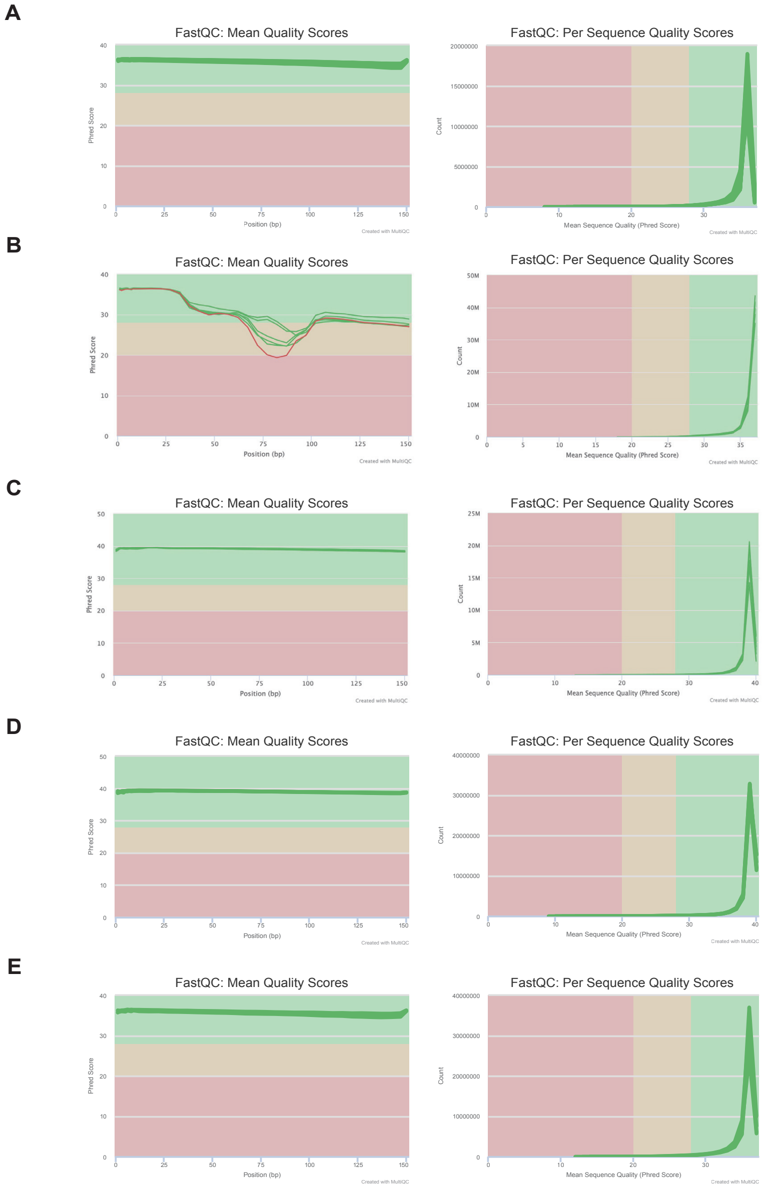

Appendix Figure S6. MultiQC results of different omics approaches in this study.

(**A**)Mean quality scores and per sequence quality scores of RNA-seq data in Figure 5A-B.

(**B**) Mean quality scores and per sequence quality scores of Ribosome profiling (Ribo-seq) data in Figure 6C.

(**C**) Mean quality scores and per sequence quality scores of Ribo-seq paired RNA-seq data in Figure 6C.

(**D**) Mean quality scores and per sequence quality scores of CUT&Tag data in Figure 7A-B.

(**E**) Mean quality scores and per sequence quality scores of ATAC-Seq data in Figure 7B.
