# Supplementary figures and images for: RNA helicase DHX29 controls the translation of transcription factors involved in germinal center response and plasma cell differentiation in mice
Source: EMBO J. 2026 May 26;45(13):4605–35. doi: 10.1038/s44318-026-00805-0 (PMC13324185; doi:10.1038/s44318-026-00805-0)

Figure 2B

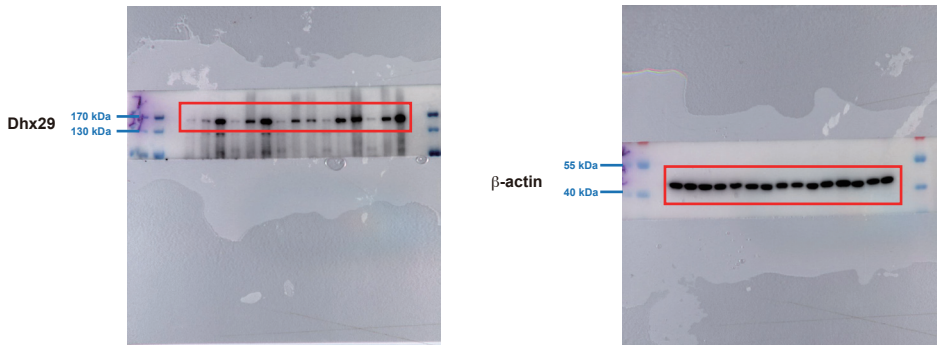

Supplement: Supplementary file 7 — Source data Fig. 2 [file 44318_2026_805_MOESM7_ESM.zip › MOESM7 Figure 2 new/Fig2B/Fig2B.pdf]

Figure 2D

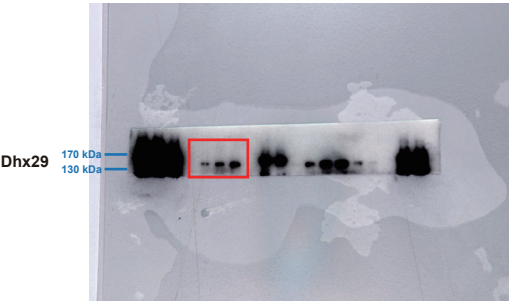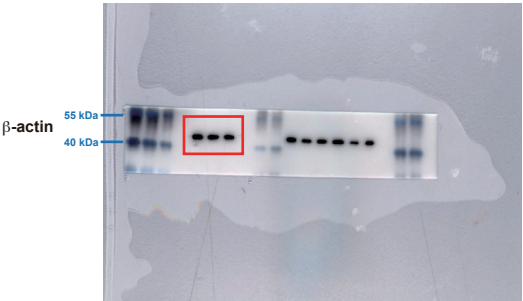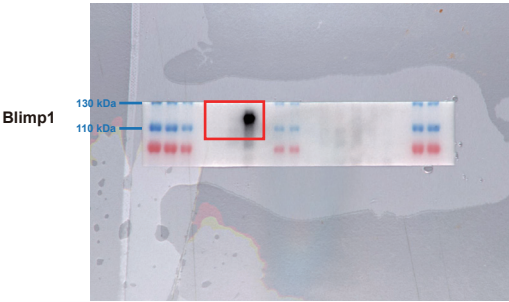

Supplement: Supplementary file 7 — Source data Fig. 2 [file 44318_2026_805_MOESM7_ESM.zip › MOESM7 Figure 2 new/Fig2D/Fig2D.pdf]

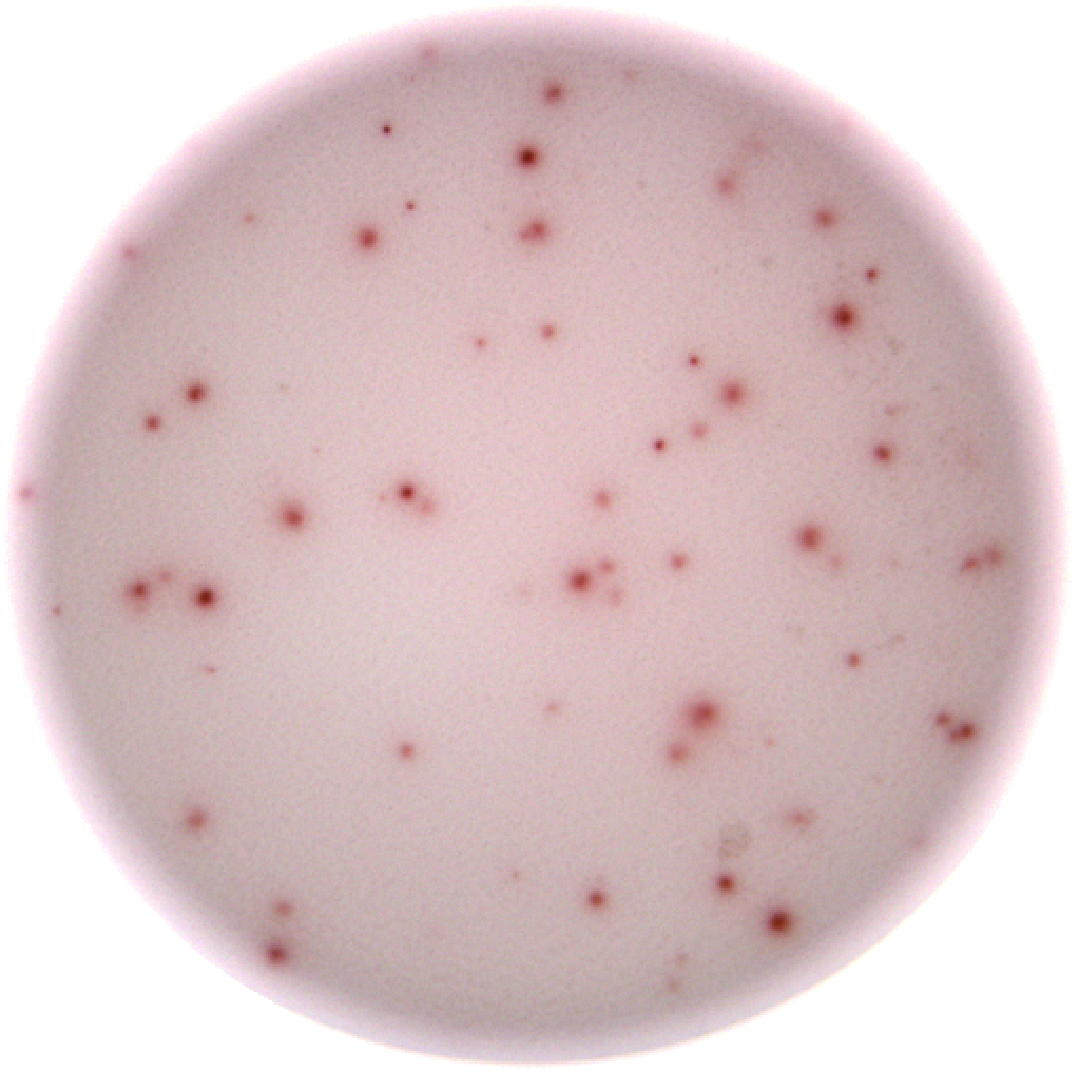

Supplement: Supplementary file 8 — Source data Fig. 3 [file 44318_2026_805_MOESM8_ESM.zip › Figure 3/Fig3B/Elispot Dhx29fl:fl.jpg]

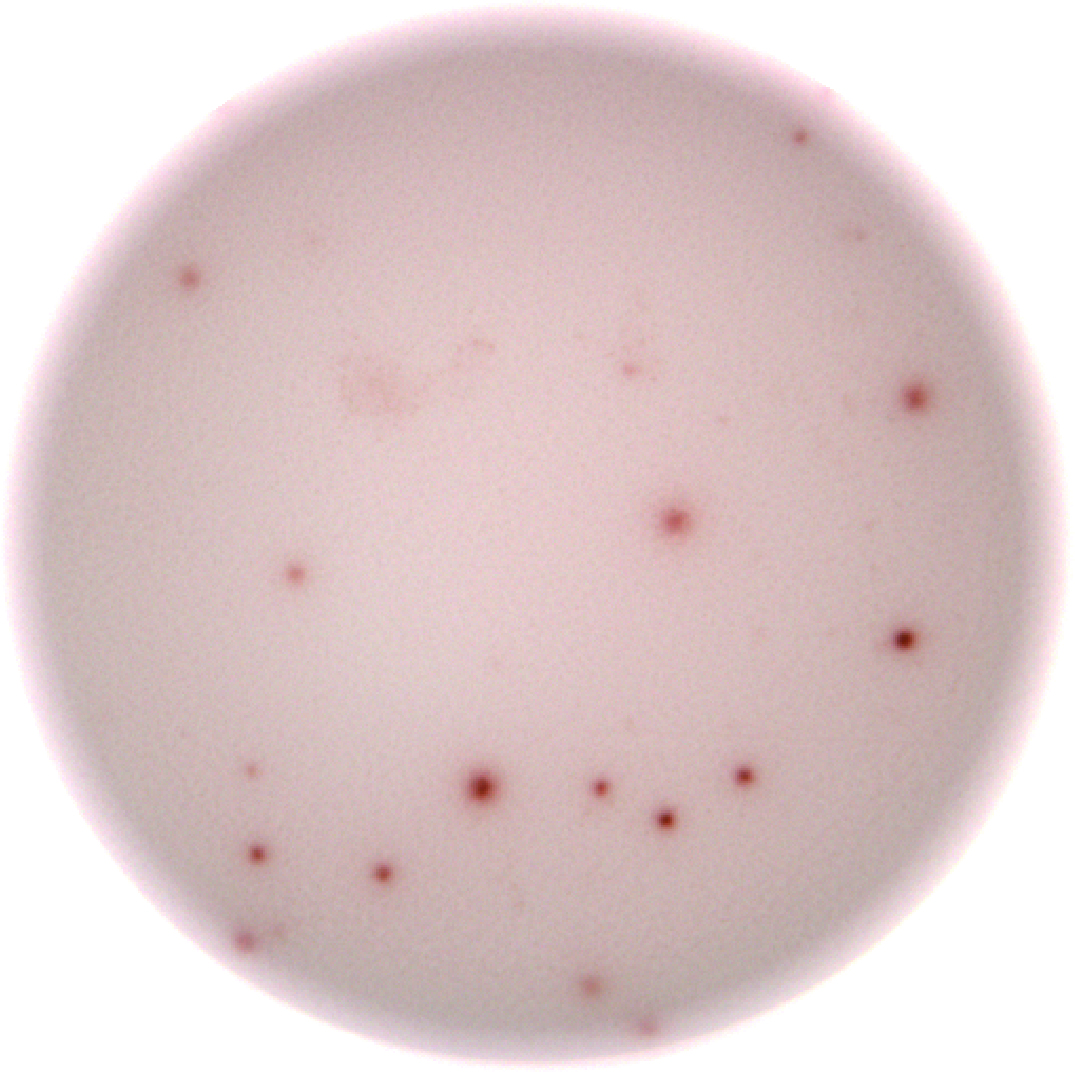

Supplement: Supplementary file 8 — Source data Fig. 3 [file 44318_2026_805_MOESM8_ESM.zip › Figure 3/Fig3B/Elispot Dhx29fl:flCg1Cre.jpg]

Figure 4B

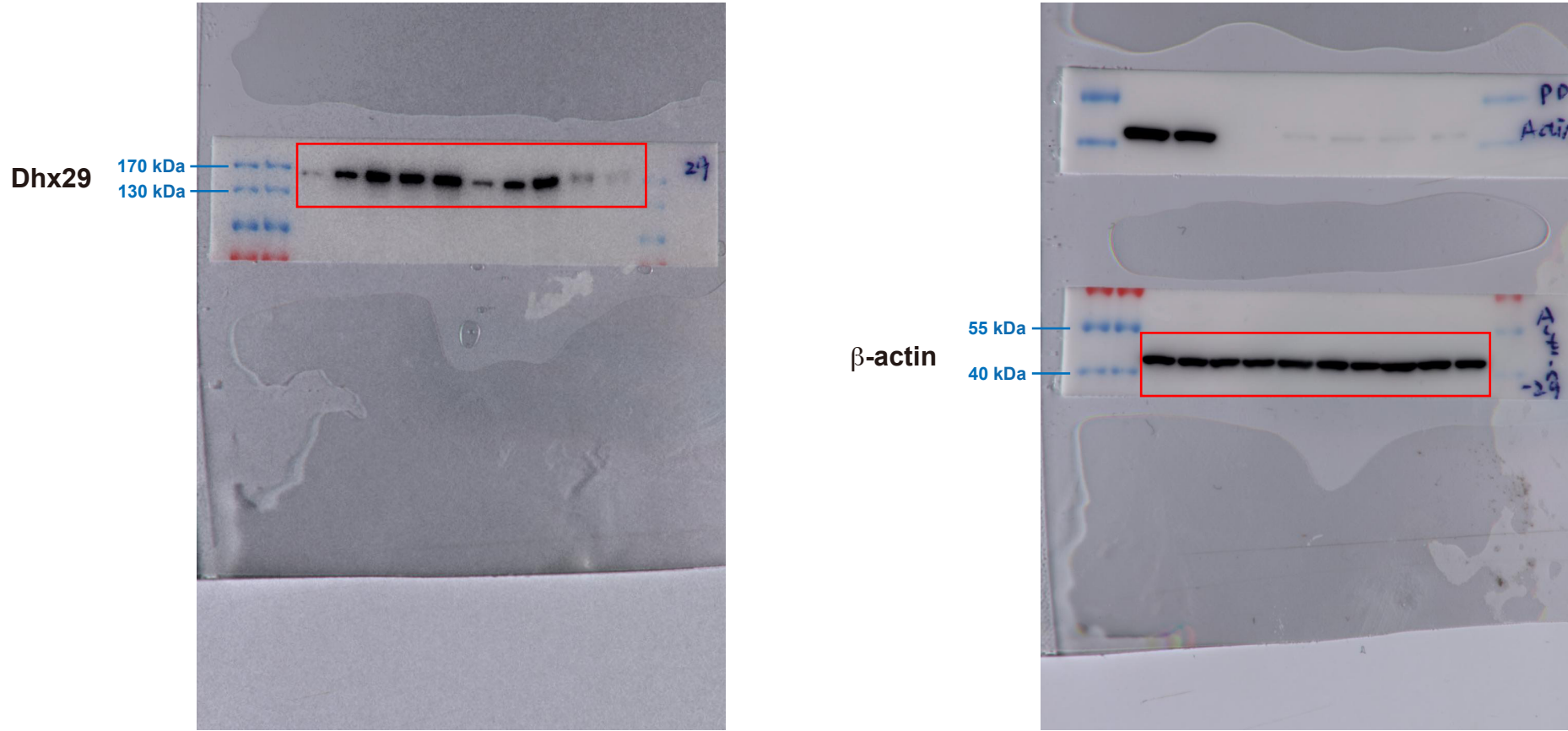

Supplement: Supplementary file 9 — Source data Fig. 4 [file 44318_2026_805_MOESM9_ESM.zip › Figure 4 /Fig4B/Fig4B.pdf]

Figure 5E

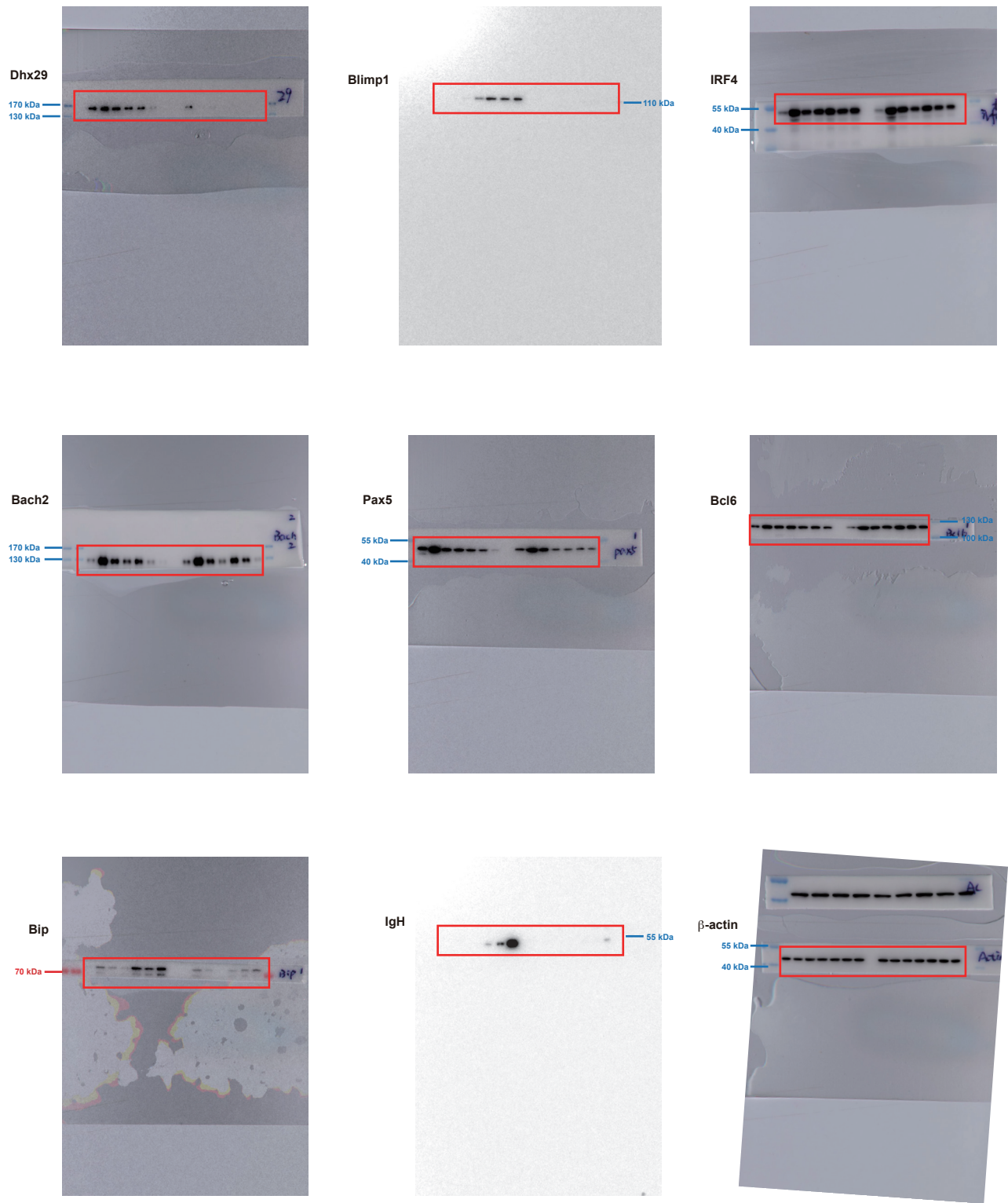

Supplement: Supplementary file 10 — Source data Fig. 5 [file 44318_2026_805_MOESM10_ESM.zip › Figure 5/Fig5E/Fig5E.pdf]

Figure 6E

Dhx29

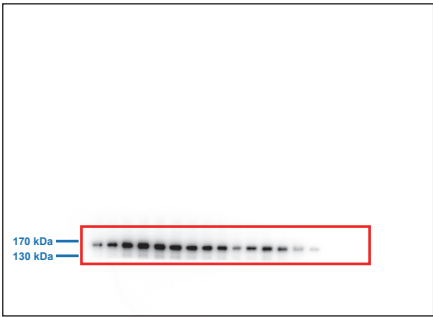

TCF3

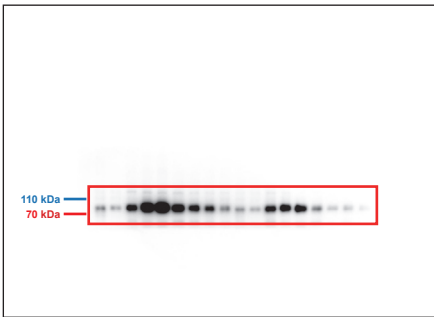

Blimp1

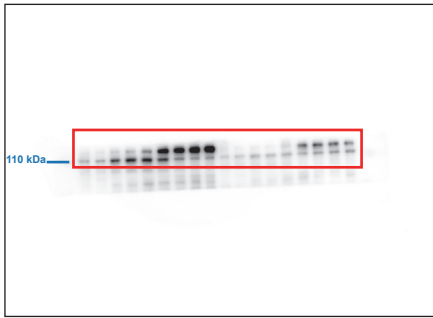

Tle3

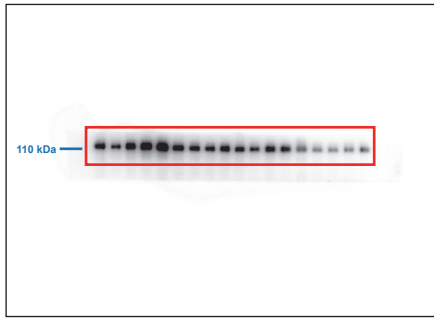

$\beta$ -actin

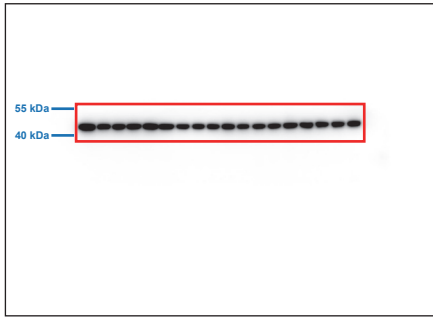

Supplement: Supplementary file 11 — Source data Fig. 6 [file 44318_2026_805_MOESM11_ESM.zip › Figure 6/Fig6E/Fig6E.pdf]

Figure 6G

Dhx29

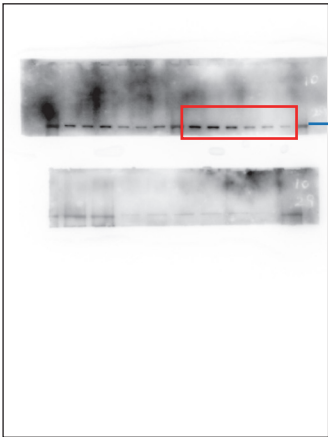

TCF3

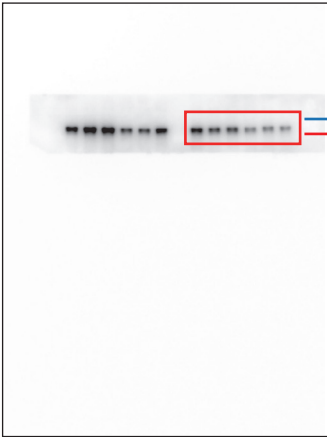

Blimp1

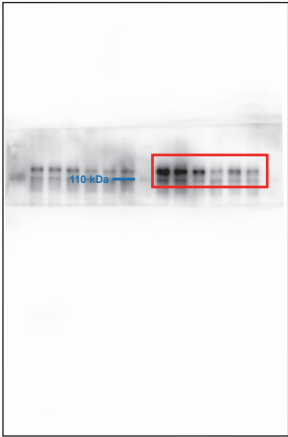

Tle3

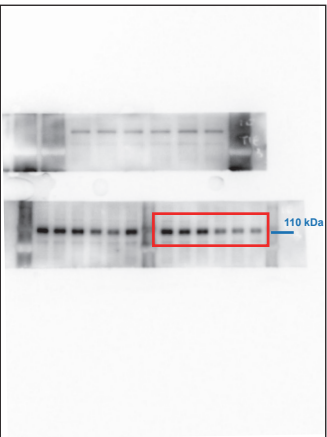

$\beta$ -actin

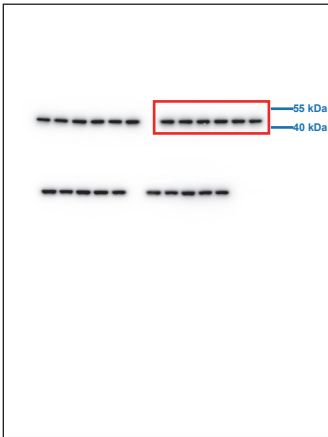

Supplement: Supplementary file 11 — Source data Fig. 6 [file 44318_2026_805_MOESM11_ESM.zip › Figure 6/Fig6G/Fig6G.pdf]

Figure 8E

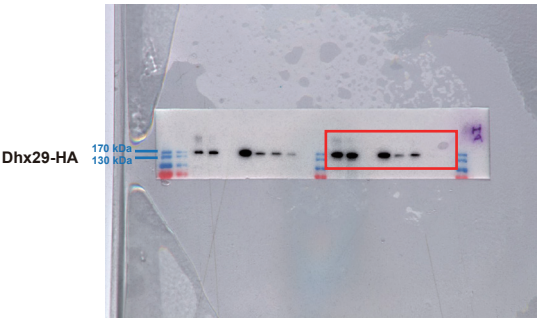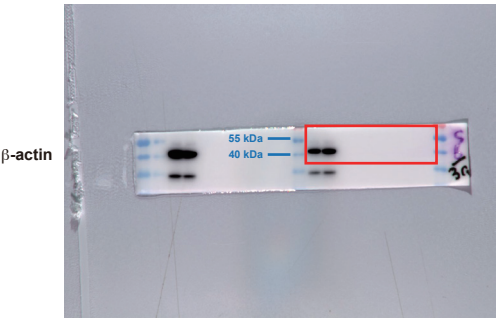

Supplement: Supplementary file 13 — Source data Fig. 8 [file 44318_2026_805_MOESM13_ESM.zip › Figure 8/Fig8E/Fig8E.pdf]

Figure 8F

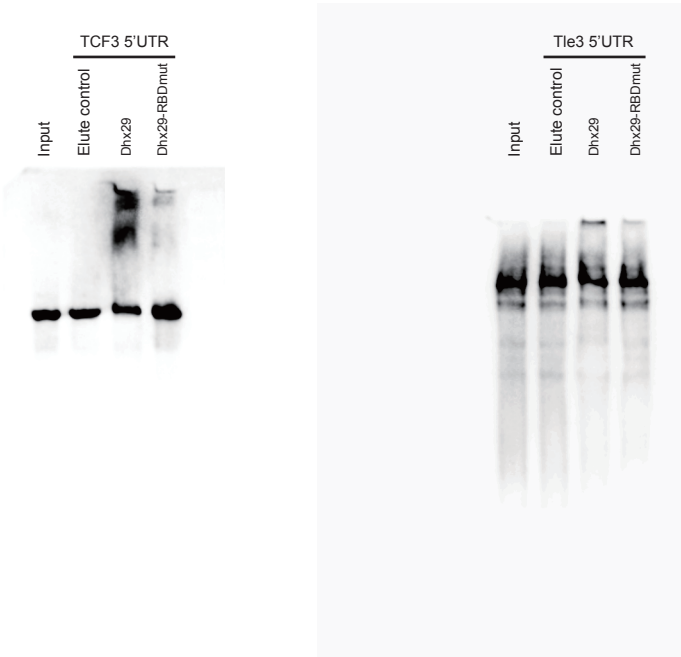

Supplement: Supplementary file 13 — Source data Fig. 8 [file 44318_2026_805_MOESM13_ESM.zip › Figure 8/Fig8F/Fig8F.pdf]
