# Supplementary material for: RNA helicase DHX29 controls the translation of transcription factors involved in germinal center response and plasma cell differentiation in mice
Source: EMBO J. 2026 May 26;45(13):4605–35. doi: 10.1038/s44318-026-00805-0 (PMC13324185; doi:10.1038/s44318-026-00805-0)
Supplement: Supplementary file 14 — Expanded View Figures [file 44318_2026_805_MOESM14_ESM.pdf]

Expanded View Figures

Figure EV1. Validation of positive hits from CRISPR/Cas9 screening.

(**A**) Naïve B cells from Cas9-GFP (CD45.2$^+$) and CD45.1$^+$ wild-type mice were mixed in a 1:1 ratio, cultured in the iGCB system, transduced with retroviruses encoding indicated sgRNAs, induced for iPC differentiation for 4 days, and analyzed by flow cytometry for the percentage of CD45.1$^+$ and Cas9-GFP$^+$ cells, as well as the percentage of iPCs (CD19$^+$CD138$^+$). (**B**) FACS gating strategy and calculation of normalized expansion deviation and differentiation deviation.

© The Author(s)

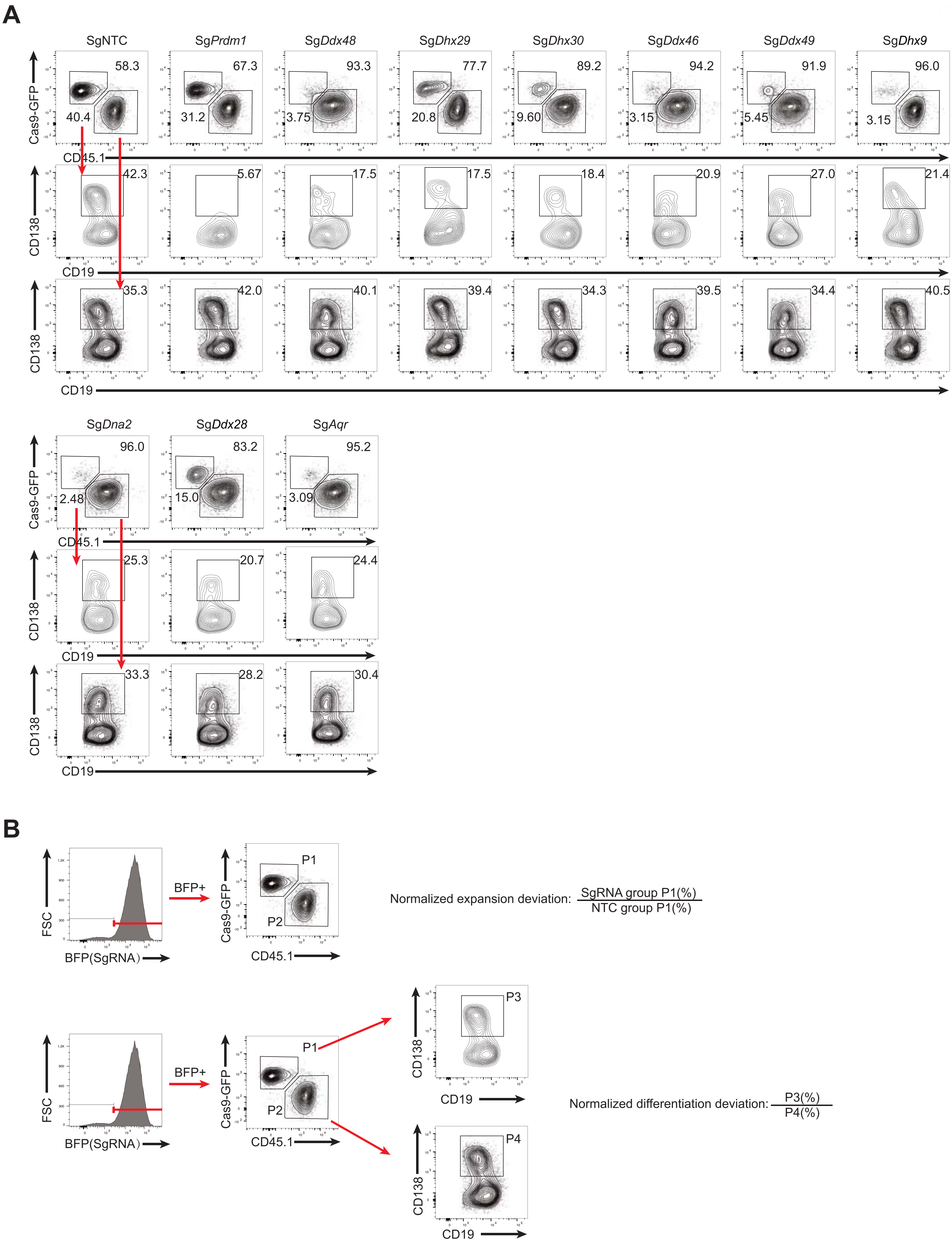
A
SgNTC
SgPrdm1
SgDdx48
SgDhx29
SgDhx30
SgDdx46
SgDdx49
SgDhx9
Cas9-GFP
CD45.1
CD138
CD19
58.3
40.4
67.3
31.2
93.3
3.75
77.7
20.8
89.2
9.60
94.2
3.15
91.9
5.45
96.0
3.15
42.3
5.67
17.5
17.5
18.4
20.9
27.0
21.4
35.3
42.0
40.1
39.4
34.3
39.5
34.4
40.5
SgDna2
SgDdx28
SgAqr
96.0
2.48
83.2
15.0
95.2
3.09
25.3
20.7
24.4
33.3
28.2
30.4
B
FSC
BFP(SgRNA)
BFP+
P1
P2
Normalized expansion deviation: SgRNA group P1(%) / NTC group P1(%)
P3
P4
Normalized differentiation deviation: P3(%) / P4(%)

 © The Author(s)

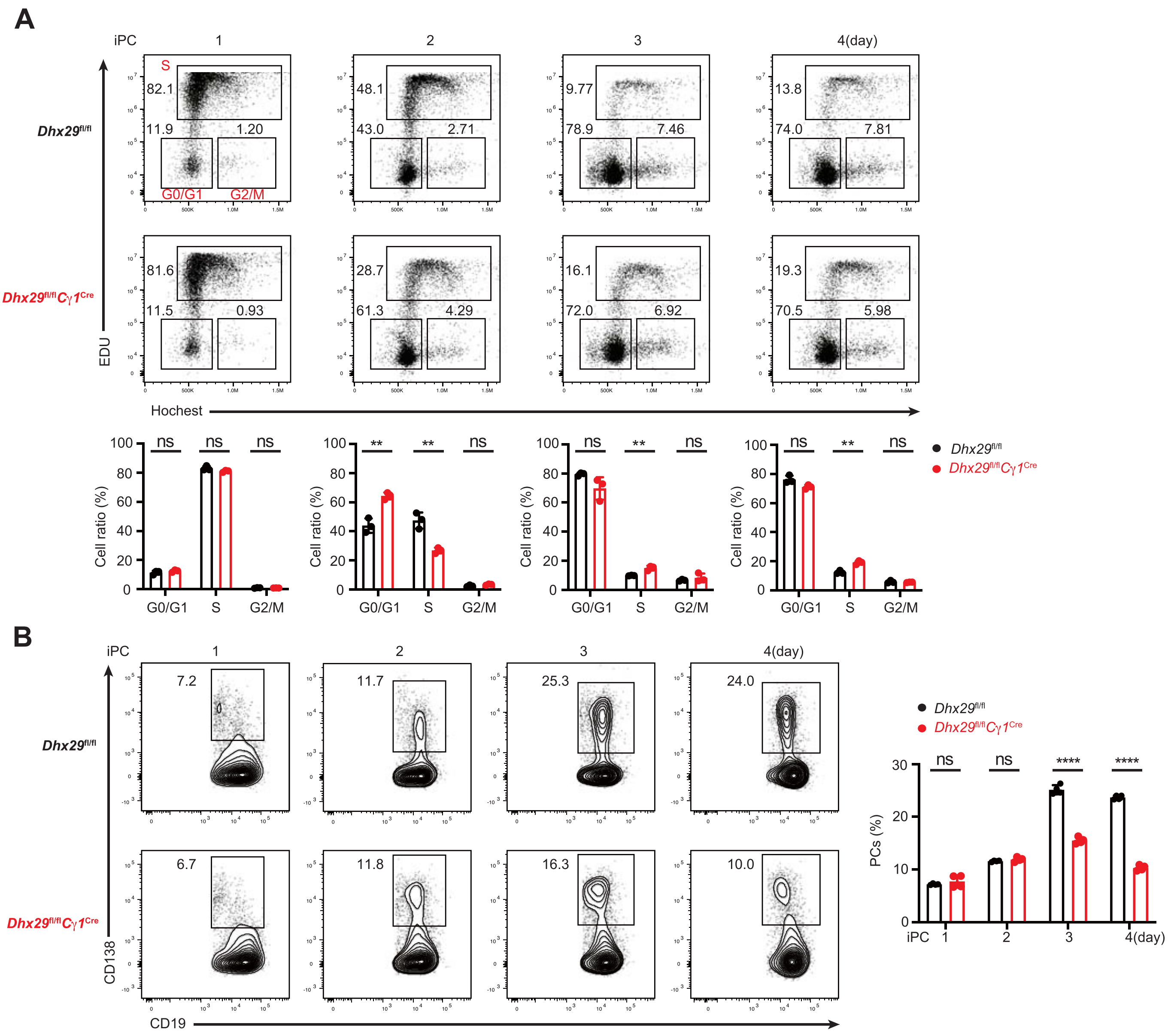

Figure EV2. Proliferation and differentiation of *Dhx29*-deficient B cells.

Flow cytometry analysis of cell cycle progression (**A**) and percentages of CD19^{+}CD138^{+} iPC cells (**B**) among *Dhx29*$^{fl/fl}$ and *Dhx29*$^{fl/fl}$*Cγ1*^{Cre} B cells at the indicated time points of iPC culture. Upper, representative FACS plots. Lower, bar graphs summarizing percentages of cells at indicated phases of cell cycle (**A**) and percentages of CD19^{+}CD138^{+} iPC cells (**B**). Data from three or four independent biological replicates (iPC D2 G0/G1: $P = 0.0030$; iPC D2 S: $P = 0.0037$; iPC D3 S: $P = 0.0053$; iPC D4 S: $P = 0.0027$; iPC D3 PCs (%): $P = 1.67E\text{-}06$; iPC D4 PCs (%): $P = 1.29E\text{-}08$). Small horizontal lines indicate the mean (± SD). Statistical significance was determined by unpaired, two-tailed Student's *t* test in (**A**, **B**). $^{**}P < 0.01$, $^{****}P < 0.0001$.

© The Author(s)

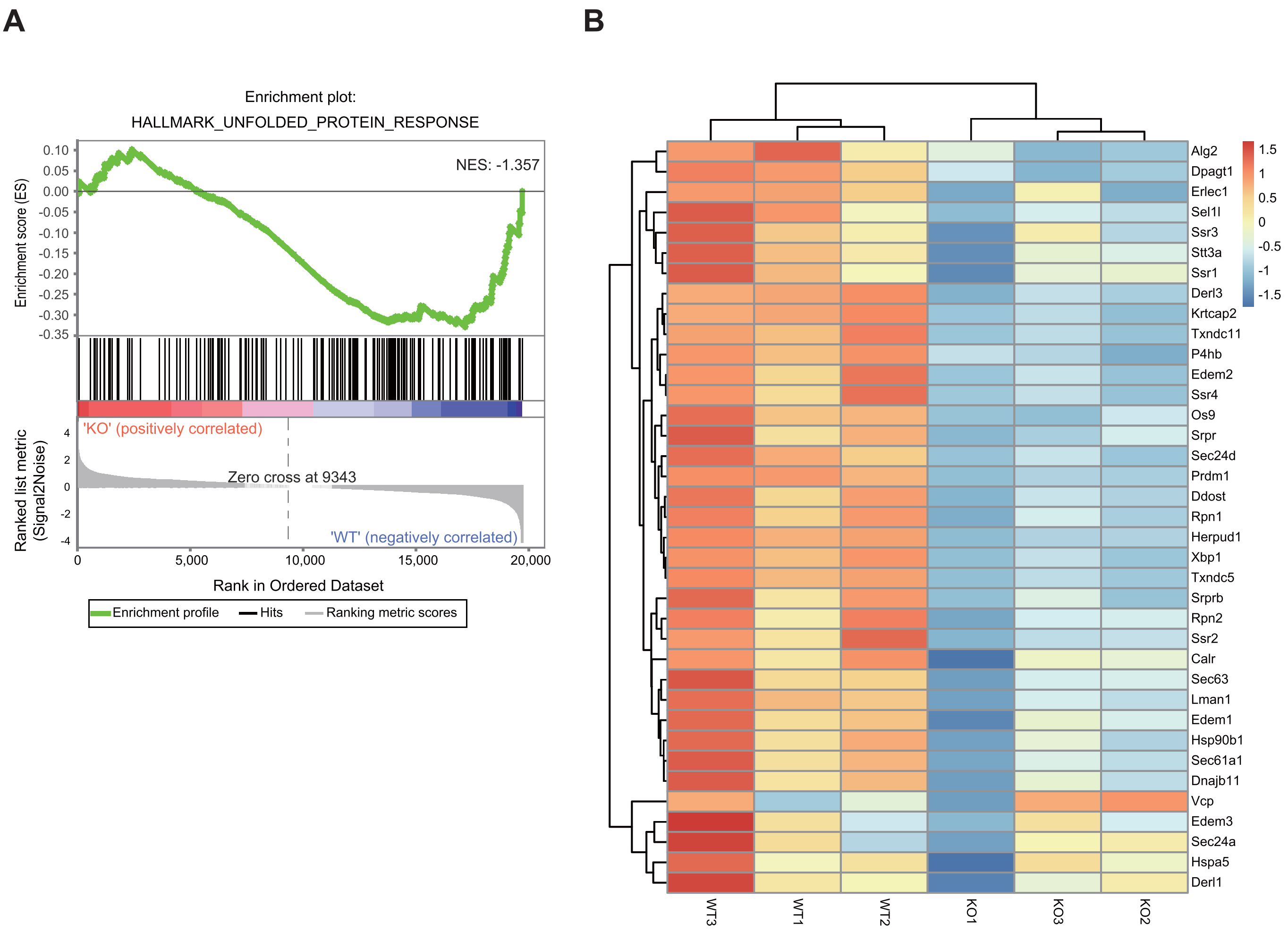

Figure EV3. Impaired expression of genes related to ER protein processing in *Dhx29*-deficient B cells.

(**A**) Gene enrichment pathway identified by GESA analysis. NES, normalized enrichment score. (**B**) Heatmap of select genes related to ER protein processing identified by RNA-Seq in Fig. 5A.

 © The Author(s)

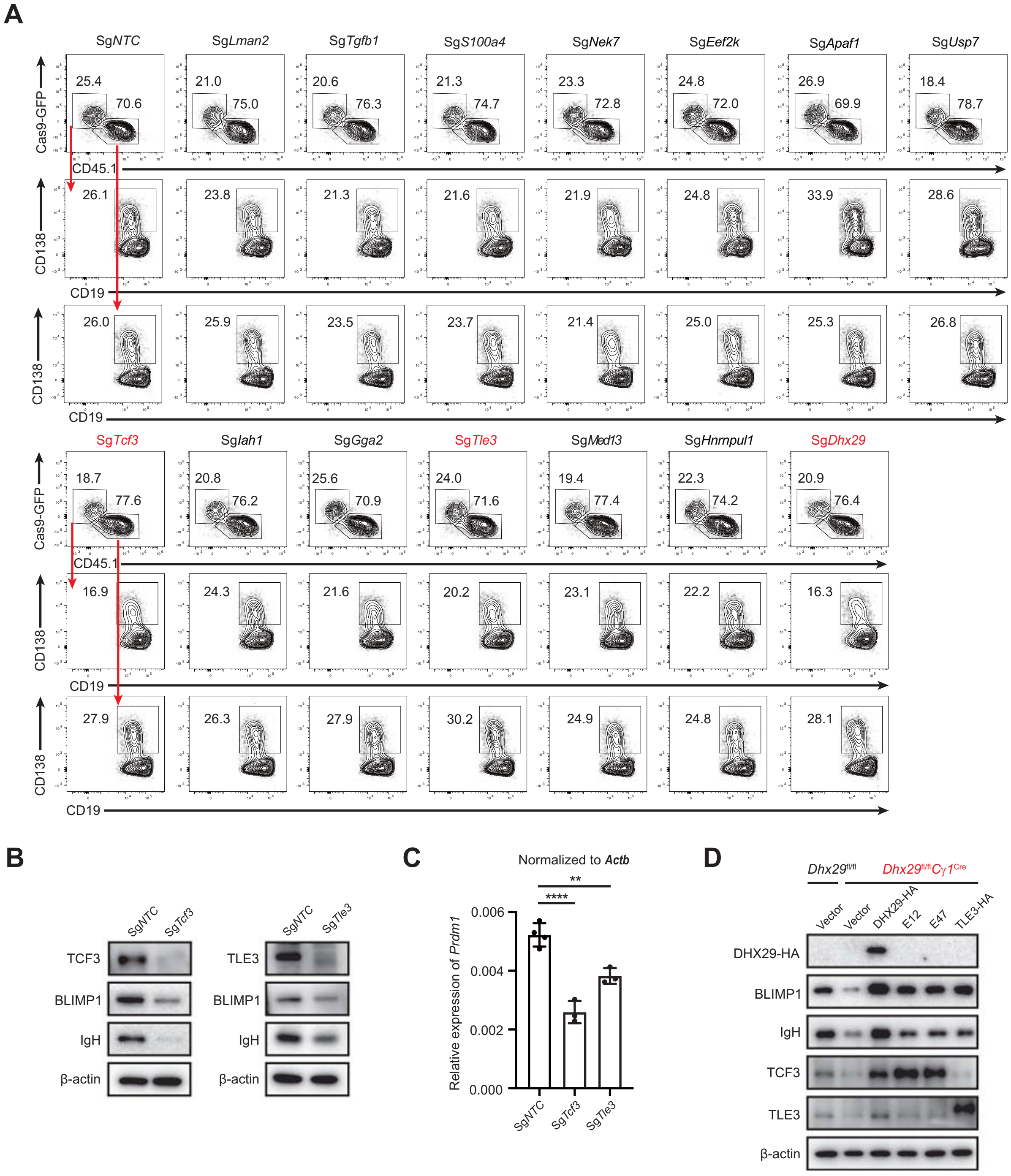

Figure EV4. Functional analysis of potential DHX29 target genes identified by mass spectrometry and ribosome profiling.

(**A**) Naïve B cells from Cas9-GFP ($CD45.2^+$) and $CD45.1^+$ wild-type mice were mixed in a 1:1 ratio, cultured in the iGCB system, transduced with retroviruses encoding indicated sgRNAs, and induced for iPC differentiation for 4 days. The percentages of $CD45.1^+$ and Cas9-GFP^+ cells, as well as iPCs ($CD19^+CD138^+$), were analyzed by flow cytometry. (**B**) Immunoblot analysis of indicated proteins in Cas9-GFP^+ B cells transduced with retroviruses encoding *Tcf3* or *Tle3* sgRNA and analyzed at iPC day 4. (**C**) Quantitative RT-PCR analysis of BLIMP1 mRNA level in Cas9-GFP^+ B cells transduced with retroviruses encoding NTC, *Tcf3*, or *Tle3* sgRNA and analyzed at iPC day 1. Data from three or four independent biological replicates (Sg*Tcf3* vs Sg*NTC*: $P =$ 5.56E-05; Sg*Tle3* vs Sg*NTC*: $P =$ 0.0027). (**D**) $Dhx29^{fl/fl}$ and $Dhx29^{fl/fl}C\gamma 1^{Cre}$ B cells were transduced with retroviruses encoding the indicated genes at iGCB day 2.5. GFP^+ cells were sorted and analyzed at iPC day 4 by immunoblot for HA-tagged DHX29 (by anti-HA antibody), BLIMP1, IgH, TCF3, and TLE3. Small horizontal lines indicate the mean (± SD). Statistical significance was determined by one-way ANOVA in (**C**). $^{**}P < 0.01$, $^{****}P < 0.0001$.

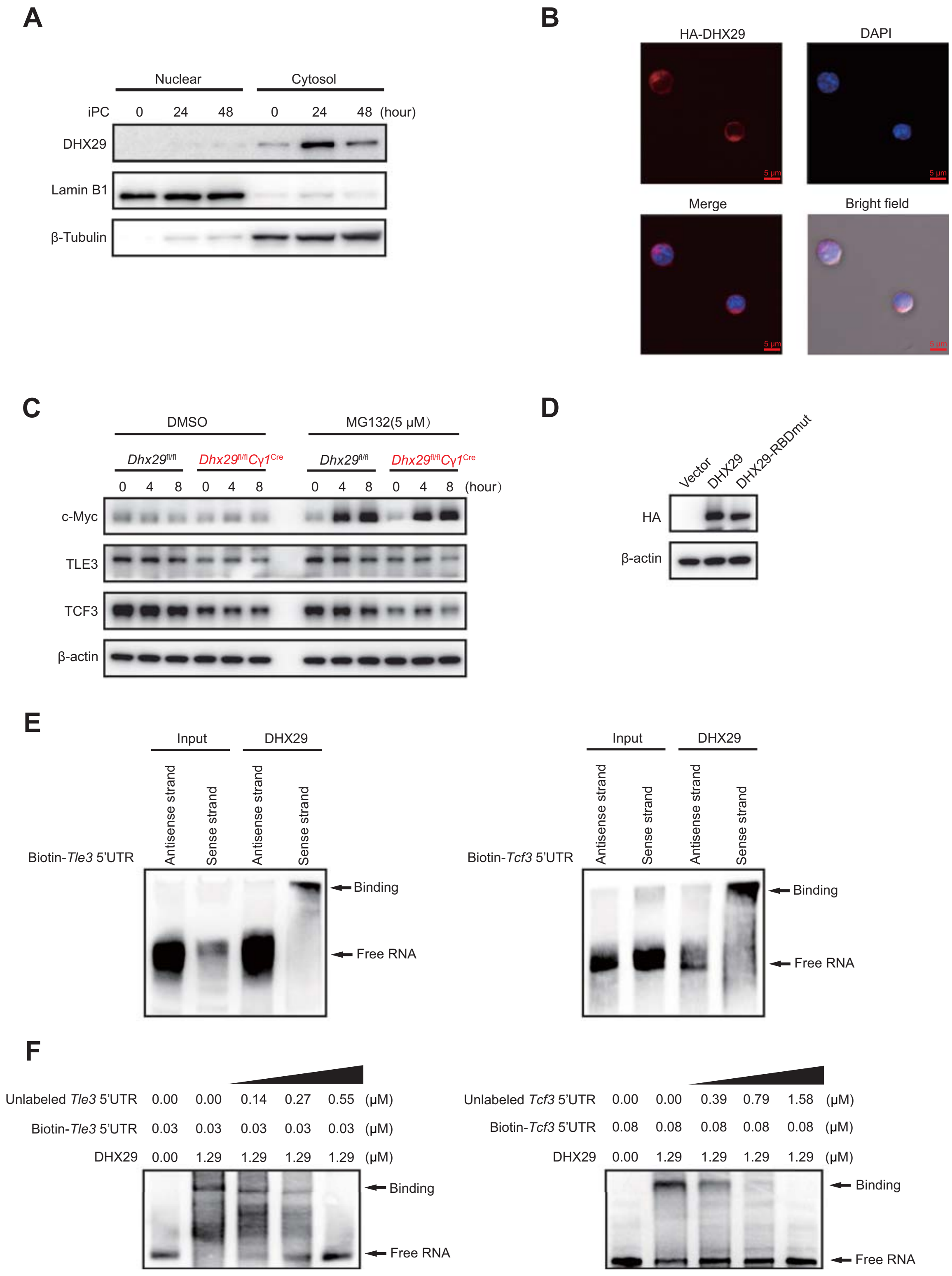
A
Nuclear
Cytosol
iPC 0 24 48 0 24 48 (hour)
DHX29
Lamin B1
β-Tubulin
B
HA-DHX29
DAPI
Merge
Bright field
C
DMSO
MG132(5 μM)
Dhx29fl/fl
Dhx29fl/flCγ1Cre
0 4 8 0 4 8 0 4 8 0 4 8 (hour)
c-Myc
TLE3
TCF3
β-actin
D
Vector
DHX29
DHX29-RBDmut
HA
β-actin
E
Input
DHX29
Antisense strand
Sense strand
Biotin-Tle3 5'UTR
Biotin-Tcf3 5'UTR
Binding
Free RNA
F
Unlabeled Tle3 5'UTR 0.00 0.00 0.14 0.27 0.55 (μM)
Biotin-Tle3 5'UTR 0.03 0.03 0.03 0.03 0.03 (μM)
DHX29 0.00 1.29 1.29 1.29 1.29 (μM)
Unlabeled Tcf3 5'UTR 0.00 0.00 0.39 0.79 1.58 (μM)
Biotin-Tcf3 5'UTR 0.08 0.08 0.08 0.08 0.08 (μM)
DHX29 0.00 1.29 1.29 1.29 1.29 (μM)
Binding
Free RNA

 © The Author(s)

Figure EV5. DHX29 promotes *Tcf3* and *Tle3* translation in the cytosol.

(**A**) Immunoblot analysis of DHX29 in the cytosolic and nuclear fractions of B cells at different time points of iPC culture. Lamin B1 and β-tubulin were used as control for nuclear and cytosolic proteins, respectively. (**B**) Representative immunofluorescence images of B cells transduced with retroviruses encoding HA-tagged DHX29 and stained for HA (red) and DAPI (blue, nuclear staining). Scale bar, 5 μm. (**C**) Immunoblot analysis of *Dhx29*$^{fl/fl}$ and *Dhx29*$^{fl/fl}$*Cγ1*^{Cre} B cells treated with DMSO and MG132 at iGCB day 3.5 for indicated amounts of time. c-Myc was used as a control. (**D**) Immunoblot analysis of DHX29 in HEK293T cells transduced with retroviruses encoding HA-tagged DHX29 or its RBD binding domain deleted mutant (DHX29-RBDmut). (**E**) EMSA analysis of DHX29 binding to the 5′UTRs of *Tle3* (left) and *Tcf3* (right). 1.29 μM DHX29 protein was incubated with 0.03 μM *Tle3* 5′UTR or 0.08 μM *Tcf3* 5′UTR labeled with biotin. The mixtures were resolved on a native TBE gel. The antisense strand of each 5′UTR was used as a negative control. (**F**) Competition-binding assay. Increasing concentrations of unlabeled *Tle3* or *Tcf3* 5′UTR were added to the reaction mixture in (**E**). The mixtures were resolved on a native TBE gel.

© The Author(s)
