# Supplementary material for: RNA helicase DHX29 controls the translation of transcription factors involved in germinal center response and plasma cell differentiation in mice
Source: EMBO J. 2026 May 26;45(13):4605–35. doi: 10.1038/s44318-026-00805-0 (PMC13324185; doi:10.1038/s44318-026-00805-0)
Supplement: Supplementary file 4 — Peer Review File [file 44318_2026_805_MOESM4_ESM.pdf]

THE EMBO JOURNAL

RNA helicase DHX29 controls the translation of transcription factors involved in germinal center response and plasma cell differentiation in mice

Jiayi Zhao, Xiaoyu He, Peicheng Hong, Lianghua Lin, Ying Du, Pengda Chen, Lixiao Zhang, JiaLe Leng, Lihui Ma, Jun Xie, Xinyong Lin, Abidan Adilijiang, Jiazhen Wang, Yazhen Hong, Zhengtao Xiao, Wen-Hsien Liu, and Changchun Xiao

Corresponding author(s): Changchun Xiao (cxiao@scripps.edu) , Wen-Hsien Liu (whliu@xmu.edu.cn)

Review Timeline:

Submission Date:	10th Jun 25
Editorial Decision:	1st Aug 25
Revision Received:	31st Jan 26
Editorial Decision:	26th Feb 26
Revision Received:	24th Mar 26
Editorial Decision:	24th Apr 26
Revision Received:	25th Apr 26
Accepted:	27th Apr 26

Editor: Ioannis Papaioannou

Transaction Report:

(Note: With the exception of the correction of typographical or spelling errors that could be a source of ambiguity, letters and reports are not edited. Depending on transfer agreements, referee reports obtained elsewhere may or may not be included in this compilation. Referee reports are anonymous unless the Referee chooses to sign their reports.)

Dear Dr. Xiao,

Thank you again for submitting your manuscript EMBOJ-2025-121591 for consideration by The EMBO Journal, and for your patience during peer review. Your manuscript has now been seen by three experts in the field, and we have received their informative and constructive reports, which you can find below.

I am pleased to say that, as you will see, the referees indicate interest in your manuscript, find your results intriguing and relevant, and point out that the mouse models that were developed in this study to investigate the function of Dhx29 are commendable. However, they also identify several major limitations in the study and the manuscript, and raise significant concerns regarding the in vivo relevance of the findings, the limited depth of the mechanistic evidence and understanding, and a number of other points that require further experimental investigation and clarification.

Given the referees' comments and recommendations, I would like to invite you to submit a thorougly revised version of your manuscript taking the referees' suggestions on board, along with a detailed point-by-point response addressing all referees' comments. I should add that we would require both further insights into the in vivo relevance of the major findings (e.g. major point 1 of referee 1), and more solid evidence for the mechanistic basis of the phenomenon (e.g. major comment of referee 3) for further consideration of the work at The EMBO Journal. All technical points should also be addressed. Please note that it is The EMBO Journal policy to allow only a single round of major revision, and acceptance of your manuscript will therefore depend on the completeness of your responses in this revised version. Please let me know if you have any questions or comments that you would like to discuss with me. If there are any major points you do not agree with or cannot address during your revision, I would encourage you to share them with me as early as possible to discuss how to proceed further in the most efficient way.

We generally allow three months as standard revision time (October 31, 2025). As a matter of policy, competing manuscripts published during this period will not negatively impact our assessment of the conceptual advance presented by your study. However, we request that you contact us as soon as possible upon publication of any related work, to discuss how to proceed. Should you foresee a problem in meeting this three-month deadline, please let us know in advance and we will be able to grant an extension.

Thank you for the opportunity to consider your work for publication in The EMBO Journal. I look forward to your revision.

Best regards,

Ioannis

Ioannis Papaioannou, PhD
Editor, The EMBO Journal
i.papaioannou@embojournal.org

*****
Instructions for preparing your revised manuscript
*****

1. When you are ready to submit the revision, please upload:

- A Word file of the manuscript text (including legends of main Figures, EV Figures and Tables). Please make sure that changes are highlighted (or "tracked") to be clearly visible.

- Individual production-quality figure files (one file per figure). When assembling your figures, please refer to our figure preparation guidelines in order to ensure proper formatting and readability in print as well as on screen:
https://bit.ly/EMBOPressFigurePreparationGuideline
If the data shown in a figure are obtained from n {less than or equal to} 2, please use scatter plots showing the individual data points.
Please also include scale bars in all microscopy images.
The following points must be specified in each figure legend:
i. the name of the statistical test used to generate error bars and P values
ii. the number (n) of independent experiments (please specify technical or biological replicates) underlying each data point (discussion of statistical methodology can be reported in the Materials and Methods section, but figure legends should contain a basic description of n, P, and the test applied)

iii. the nature of the bars and error bars (s.d., s.e.m.).
See also guidelines for figure legends: https://www.embopress.org/page/journal/14602075/authorguide#figureformat

- A point-by-point response to the referees' comments, with a detailed description of the changes made (as a word file). All referees' concerns must be fully addressed and their suggestions taken on board. When preparing your letter of response to the referees' comments, please bear in mind that this will form part of the Review Process File and will therefore be available online to the community. Please note that you have the possibility to opt out of the transparent process at any stage prior to publication by letting the editorial office know (contact@embojournal.org); if you do opt out, the Review Process File link will point to the following statement: "No Review Process File is available with this article, as the authors have chosen not to make the review process public in this case.". For more details on our Transparent Editorial Process, please visit our website: https://www.embopress.org/page/journal/14602075/authorguide#transparentprocess

- Expanded View (EV) files (replacing Supplementary Information) that are collapsible/expandable online. A maximum of 5 EV Figures can be typeset. EV Figures should be cited as "Figure EV1, Figure EV2" etc. in the text, and their respective legends should be included in the manuscript file after the legends of regular figures. See detailed instructions regarding Expanded View files here:
https://www.embopress.org/page/journal/14602075/authorguide#expandedview

- For the figures that you do NOT wish to display as Expanded View figures, they should be bundled together with their legends in a single PDF file called "Appendix", which should start with a short Table of Contents (including page numbers). Appendix figures should be referred to in the main text as: "Appendix Figure S1, Appendix Figure S2" etc. Please see detailed instructions here: https://www.embopress.org/page/journal/14602075/authorguide#expandedview

- A complete author checklist, which you can download from our author guidelines (https://www.embopress.org/page/journal/14602075/authorguide). Please note that the checklist will also be part of the Review Process File.

2. Please note that no statistics should be calculated and shown in Figures if n=2. Please also note that each p value should be reported as an exact value.

3. Before submitting your revision, primary datasets (and computer code, where appropriate) produced in this study need to be deposited in appropriate public databases (see https://www.embopress.org/page/journal/14602075/authorguide#dataavailability). In particular, you are kindly requested to deposit all DNA and RNA sequencing data, as well as the mass spectrometry data generated in the study. The accession numbers, database, and the specific URLs (links) should be listed in a formal "Data availability" section (placed after Methods), following the example below:

"The RNA-seq datasets produced in this study are available in the following database:
Gene Expression Omnibus GSE46843 (https://www.ncbi.nlm.nih.gov/geo/query/acc.cgi?acc=GSE46843)"

*** All links should resolve to a page where the data can be accessed. ***

*** Please remember to provide in the Data availability section of your revised manuscript reviewer passwords if the datasets are not yet public. ***

*** The Data Availability Section is restricted to new primary data that are part of this study. In case you have no data that require deposition in a public database, please state so instead of referring to the database: "Our study includes no data deposited in public repositories." under the heading "Data availability". ***

4. The materials and methods need to be described in the manuscript using our structured methods format, which is now required for all research articles. According to this format, the Methods section includes a single "Reagents and Tools Table" - listing key reagents, experimental models, software and relevant equipment including their sources and relevant identifiers- followed by a "Methods and Protocols" section describing the methods. Please download and fill our Reagents and Tools Table template (.docx), which you can find in our author guide:
https://www.embopress.org/page/journal/14602075/authorguide#structuredmethods. When submitting your revised manuscript, please do not include the Reagents and Tools Table in the Methods section of the manuscript but instead upload it as a separate file choosing the file type "Reagent Table".

5. Please check that the title and the abstract of the manuscript are brief, yet explicit, even to non-specialists. The length of the title should not exceed 100 characters, and the abstract should be a single paragraph not exceeding 175 words.

6. Please also note our reference format: https://www.embopress.org/page/journal/14602075/authorguide#referencesformat.

7. At EMBO Press we ask authors to provide source data for the main manuscript figures. You will receive a separate email with instructions for providing source data with your revised manuscript, including how to upload and organize the files.

8. Please remember: digital image enhancement is acceptable practice, as long as it accurately represents the original data and conforms to community standards. If a figure has been subjected to significant electronic manipulation, this must be noted in the figure legend or in the "Materials and Methods" section. The editors reserve the right to request original versions of figures and the original images that were used to assemble the figure.

9. Our journal encourages inclusion of data citations in the reference list to directly cite datasets that were obtained from public databases. Data citations in the article text are distinct from normal bibliographical citations and should directly link to the database records from which the data can be accessed. In the main text, data citations are formatted as follows: "Data ref: Smith et al, 2001" or "Data ref: NCBI Sequence Read Archive PRJNA342805, 2017". In the Reference list, data citations must be labeled with "[DATASET]". A data reference must provide the database name, accession number/identifiers, and a resolvable link to the landing page from which the data can be accessed at the end of the reference. Further instructions are available at: https://www.embopress.org/page/journal/14602075/authorguide#referencesformat.

10. We request authors to consider both actual and perceived competing interests. Please review our policy (https://www.embopress.org/page/journal/14602075/authorguide#conflictsofinterest) and update your competing interests statement if necessary. Please name this section 'Disclosure and competing interests statement' and place it after the Acknowledgements section.

11. Please note that all corresponding authors are required to provide an ORCID ID upon submission of a revised manuscript (https://orcid.org/). Please find instructions on how to link your ORCID ID to your account in our manuscript tracking system in our Author guidelines
(https://www.embopress.org/page/journal/14602075/authorguide#authorshipguidelines).

12. We use CRediT to specify the contributions of each author in the journal submission system. CRediT replaces the author contribution section, which should be removed from the manuscript. Please use the free text box to provide more detailed descriptions. See also guide to authors:
https://www.embopress.org/page/journal/14602075/authorguide#authorshipguidelines.

13. Further information is available in our Guide For Authors: https://www.embopress.org/page/journal/14602075/authorguide

14. We would also welcome the submission of cover suggestions or motifs to be used by our Graphics Illustrator in designing a cover.

15. Please use the link below to submit your revision:
https://emboj.msubmit.net/cgi-bin/main.plex

Referee #1:

In the manuscript entitled 'Translational control of germinal center response and plasma cell differentiation by RNA helicase Dhx29' Zhao et al. claim that selective translational control by the RNA binding protein Dhx29 of the transcription factors encoded by Tcf3 and Tle3 is needed for the germinal centre response. Using a Crispr-Cas9 screening, the authors identified Dhx29 amongst a list of 72 helicases as one of the few involved in plasma cell differentiation rather than in cell expansion. Then, using different mouse models, authors show the requirement of Dhx29 expression for the development of different B cell subsets and maintenance of germinal centre and antibody responses. Links to downstream regulation of Prdm1, encoding BLIMP-1, is provided as a potential mechanism for the differentiation of antibody-producing cells. Our understanding on how post-transcriptional mechanisms control adaptive immunity is limited. Thus, this report showing the requirement of transcript-specific regulatory mechanisms controlling mRNA translation is of interest. Unfortunately, not all claims are supported with evidence. Most observations are made using an in-vitro model of GC-like cell expansion and differentiation, however relevant findings are translated into an appropriate in-vivo setting.
Major comments:
1. Authors should attend to translate major finding from in-vitro iGC cell cultures to in-vivo settings. Major claims are made across the manuscript on the fact that Dhx29 is required for GC formation and class-switch recombination without formal demonstration. Authors should assess early B cell activation, establishment of germinal centres and maintenance over time. Using the Dhx29fl/fl C 1CRE , it is key to assess timely gene deletion, loss of protein expression and class switching. In-vivo, the analysis should be done before and after establishment of the germinal centre and, in-vitro, gene deletion couple to class-switching should be tested. It is expected that GCs are constituted by a mixed of non-classed switched and class-switched GC B cells, antigen-specific and non-antigen specific GC B cells. Thus, further characterization of these germinal centres is needed to

know whether establishment, maintenance, expansion/cycling of class-switched and/or antigen-specific GC cells and/or differentiation of GC B cells is affected. Both competitive and non-competitive settings are required to assess the contribution of protein-sufficient and protein-depleted B cells.
2. Following this argument, the authors should explain why NP-IgM titres are reduced in the Dhx29fl/fl C 1CRE mice. Presumably, IgM+ GC B cells and plasma cells should still express DHX29. Similarly, authors should explain which is the percentage of cells that undergo class-switching from IgM to IgG1 or IgE at all different stages of their culture. Also, they should explain why IgE+ iGC B cells are expanded in their in-vitro settings as this normally occurs after class-switch recombination to IgG1. Again, timely analysis of protein depletion in Dhx29fl/fl C 1CRE cells is needed.
3. A significant body of the manuscript reports alterations in B-cell development using Dhx29fl/fl CD19CRE and Dhx29fl/fl Mb1CRE mice without providing further explanation. The RNA targets and mechanisms regulated by Dhx29 are likely very different to those reported in activated B cells and GCs. Thus, these models are largely irrelevant to later
4. Protein expression data should complement quantitation of Dhx29 mRNA abundance in figure 2A, 2B and 2C. Authors should attempt to explain the differences in Dhx29 mRNA abundance between in-vitro activated B cells and GC B cells. This is important as different immunoblotting data show that Dhx29 is not detected in naïve follicular B cells. Thus, is it expressed in GC B cells at the protein level?
5. One critical aspect is that apparently, BLIMP1 expression is defective from as soon as the iGCB d4 stage. However, the percentage of CD138+ cells is similar until iPC d6 without further explanation. Consistent measurement of CD138+ or CD138- and IgG1+ or IgG1- cell cycle progression (in fig. EV6A, cells in S phase varies from 80% to 10% without further explanation why), cell expansion and survival should be performed.
6. In Figure 4, authors focus their attention on BLIMP1 without further explanation. Transcriptomics analyses are done at iPC 0, 12 and 24 hours, but no attempt to assess common or different alteration in mRNA abundance is done. Is Prdm1 altered at al time points? Are there other relevant targets being identified? Are Tle3 and Tcf3 altered at any time point? Are any of the genes identified in iGC or iPC cells differentially expressed in-vivo? And which is their functional relevance for the GC response and differentiation of PCs?
7. Authors move to iGCB d3.5 to integrate transcriptomics, translatomics and proteomics. First, information about whether the RNAseq data is obtained from the same cells used for ribosome profiling is not indicated. Second, there is no information provided about the quality and reproducibility of these assays. Third, the rational to focus the attention on Tle3 and Tcf3 is clear. Fourth, no attempts are made to validate TCF3 as a key transcriptional factor regulating Prdm1.
8. It remains unclear by which mechanism TCF3 and TLE3 controls Prdm1 gene transcription. Luciferase assays are provided for TLE3 but no relevant experimental validation is done in iGC B cells and in primary GC B cells.
9. Polysome profiling assays are little informative without properly testing reproducibility and quantification of binding to the 40S ribosome.
10. The direct binding of Dhx29 to the 5'UTR of Tcf3 and Tle3 mRNAs is not formally proven. Methods such as cross-linking immunoprecipitation and sequencing are capable to identify this type of interactions, with identification of the putative elements and nucleotides bound by the protein for later biochemical validation (e.g. mutation of binding sites). Analysis of Dhx29 to relevant mRNA targets in primary GC B cells should be attempted.
11. Rescue of phenotypes is done on iGC cell cultures. However, these should be attempted in-vivo in the mouse model.
12. In general, methods and figure legends are poorly described across the manuscript. There is a lack of clarity on whether authors are representing technical replicates of biological replicates in each of their in-vitro experiments. Attempts to pool together or represent data from independent experiments is desirable to assess reproducibility. The type of statistical test should be indicated for each figure. Data quality analyses for each of the different omics approaches used across the manuscript should be presented.

Referee #2:

The study of Zhao and colleagues investigates a role Dhx29 in humoral immunity. The authors used a boutique CRIPSR/Cas9 screen to identify RNA helicases involved in vitro B cell differentiation. Dhx29 was identified as being more essential for plasma cell differentiation comparted to B cell proliferation/ survival. The authors then made multiple B cell specific conditional KOs of Dhx29 and showed that it is important for the GC response, plasma cell differentiation and antibody production. As Dhx29 was thought to control mRNA translation, the authors performed quantitative mass spectrometry and then searched for proteins that were reduced in the Dhk29 KO while the mRNA level was unchanged. They ultimately identified TCF3 and TLE3 as being key targets of Dhx29 controlling plasma cell differentiation, potentially through the regulation of Prdm1/Blimp1.

Overall, this is a very nicely conducted study that addresses and interesting and understudied area of B cell biology. The authors have developed excellent mouse models to address the function of Dhx29, the figures are clear, and the manuscript well written.

I have the following concerns that should be addressed.

Major comments
1. Figure 2 DHX29 expression. The data in panel A and C suggest reasonably dynamic expression of Dhx29 mRNA. However, the expression values shown are not consistent with databases such as Immgen and the authors own protein data (Figure 4E) that shows no Dhx29 protein in naïve B cells and the expression in in vitro PCs is actually lower than activated B cells. I suggest

additional analysis protein (by western blotting or intracellular FACS) of in some ex vivo B cell populations, particularly those populations panel C with bring some more clarity to this issue.
2. Figure 3. The authors conclude that Dhx29 is indispensable for IgG1 class switch recombination. However, they are measuring the process only during the PC differentiation phase of the culture. As the CSR occurs prior to PC differentiation and requires IL4, then the experiments shown in fig 4G-H needs to be conducted in the iGCB phase, presumably day 3-4 when Dhx29 protein is absent. Finally, have the IgM+ cells been excluded from panel G.
3. Figure 6. The data is figure 6 are not convincing. The CUT&TAG product approach for TLE3 in panel A should be sequenced and a more non-biased view of TLE3 chromatin association in both Prdm1 and more globally. Moreover, the changes in luciferase expression shown are very minor. I suggest that the data in figure 6 does not offer much insight beyond the change in Blimp1 expression shown in figure 5H after TLE3 overexpression. I suggest either improving the analysis as outlined above or perhaps removing figure 6 from the manuscript.

Minor comments
4. Figure1B. Does the data point shown in the dot plot represent an individual sgRNA or the mean of 3 independent guides. This is not clear in the legend.

5. The authors should be more careful with the conclusions. On page 8 they conclude that "Dh29 is required for GC response, PC differentiation etc" and the section heading suggests that "Dhx29 is indispensable for PC differentiation and CSR to IgG1". I feel that Dhx29 being required for "optimal" differentiation is a more accurate description of the data presented, as some KO cells are clearly present at each stage.

Referee #3:

The authors have carried out a study that reveals an important role of the Dhx29 helicase in promoting the translation of mRNAs that control germinal center response and plasma cell differentiation. Using a CRISPR/Cas9 screen the authors identify Dhx29 as a regulator of the immune system. Interestingly, the authors identify TCF3 and Tle3 as mRNAs needing Dhx29 for their efficient translation. Consistently, expression of TCF3 and Tle3 largely overcomes the deletion of Dhx29 in restoring plasma cell differentiation.

Overall, this study provides important information about a novel pathway that Dhx29 regulates in vivo. The experiments are rigorously carried out and the overall model is convincing. The main limitation of the study, however, is that the authors propose a mechanism by which Dhx29 promotes the translation of TCF3 and Tle3 by binding directly to their 5'UTRs (this is even stated in the abstract). I find that the data to support this direct binding model is weak and unless additional data are provided this mechanism should be toned down. Specifically, the immunoprecipitation experiment identifies reduced binding of TCF3 and Tle3 to Dhx29, but this experiment does not in fact determine if the interaction is direct or indirect. It is entirely possible that the interaction of Dhx29 with TCF3 and Tle3 is occurring indirectly through the 40S subunit, which Dhx29 binds directly to. Additional experiments would therefore need to be carried out to directly test if Dhx29 specifically binds to the 5' UTRs of these mRNAs if the authors want to propose this model. Otherwise, the data provide good information about the functional significance of Dhx29 promoting the translation of these mRNAs, but do not provide a mechanism that supports this model.

We sincerely thank the reviewers for their thorough evaluation of our manuscript. Their insightful and constructive comments have been invaluable in improving the quality and rigor of this work. In response, we have performed numerous additional experiments and substantially revised the manuscript to address all concerns raised. We believe the revised version represents a significant improvement. Below, we provide a point-by-point response to each comment.

Referee #1:
In the manuscript entitled 'Translational control of germinal center response and plasma cell differentiation by RNA helicase Dhx29' Zhao et al. claim that selective translational control by the RNA binding protein Dhx29 of the transcription factors encoded by Tcf3 and Tle3 is needed for the germinal center response. Using a Crispr-Cas9 screening, the authors identified Dhx29 amongst a list of 72 helicases as one of the few involved in plasma cell differentiation rather than in cell expansion. Then, using different mouse models, authors show the requirement of Dhx29 expression for the development of different B cell subsets and maintenance of germinal center and antibody responses. Links to downstream regulation of Prdm1, encoding BLIMP-1, is provided as a potential mechanism for the differentiation of antibody-producing cells. Our understanding on how post-transcriptional mechanisms control adaptive immunity is limited. Thus, this report showing the requirement of transcript-specific regulatory mechanisms controlling mRNA translation is of interest. Unfortunately, not all claims are supported with evidence. Most observations are made using an in-vitro model of GC-like cell expansion and differentiation, however relevant findings are translated into an appropriate in-vivo setting.

Major comments:

1. Authors should attend to translate major finding from in-vitro iGCB cell cultures to in-vivo settings. Major claims are made across the manuscript on the fact that Dhx29 is required for GC formation and class-switch recombination without formal demonstration. Authors should assess early B cell activation, establishment of germinal centers and maintenance over time.

Response: As suggested by the reviewer, we examined early B cell activation upon various stimulation, including iGCB culture (stimulated by Baff, CD40L, and IL-4), LPS, LPS+IL-4. As shown in Reviewer Figure 1A, B, *Cγ1*^{Cre}-mediated deletion of *Dhx29* had no effect on B cell activation, as indicated by normal upregulation of activation markers (CD69, MHC II, CD80 and CD86). We also examined establishment and maintenance of germinal centers in *Dhx29*$^{fl/fl}$*Cg1*^{Cre} mice at various time points post immunization with OVA/alum. As shown in Reviewer Figure 1C, the percentage of GCB cells was markedly reduced at day 7, 14, and 21 post immunization, while the proliferation (EDU^{+} cells) and death (Annexin V^{+}7-AAD^{+} cells) of GCB cells were normal. Those results suggest that the establishment of germinal centers was impaired, while their maintenance was likely normal.

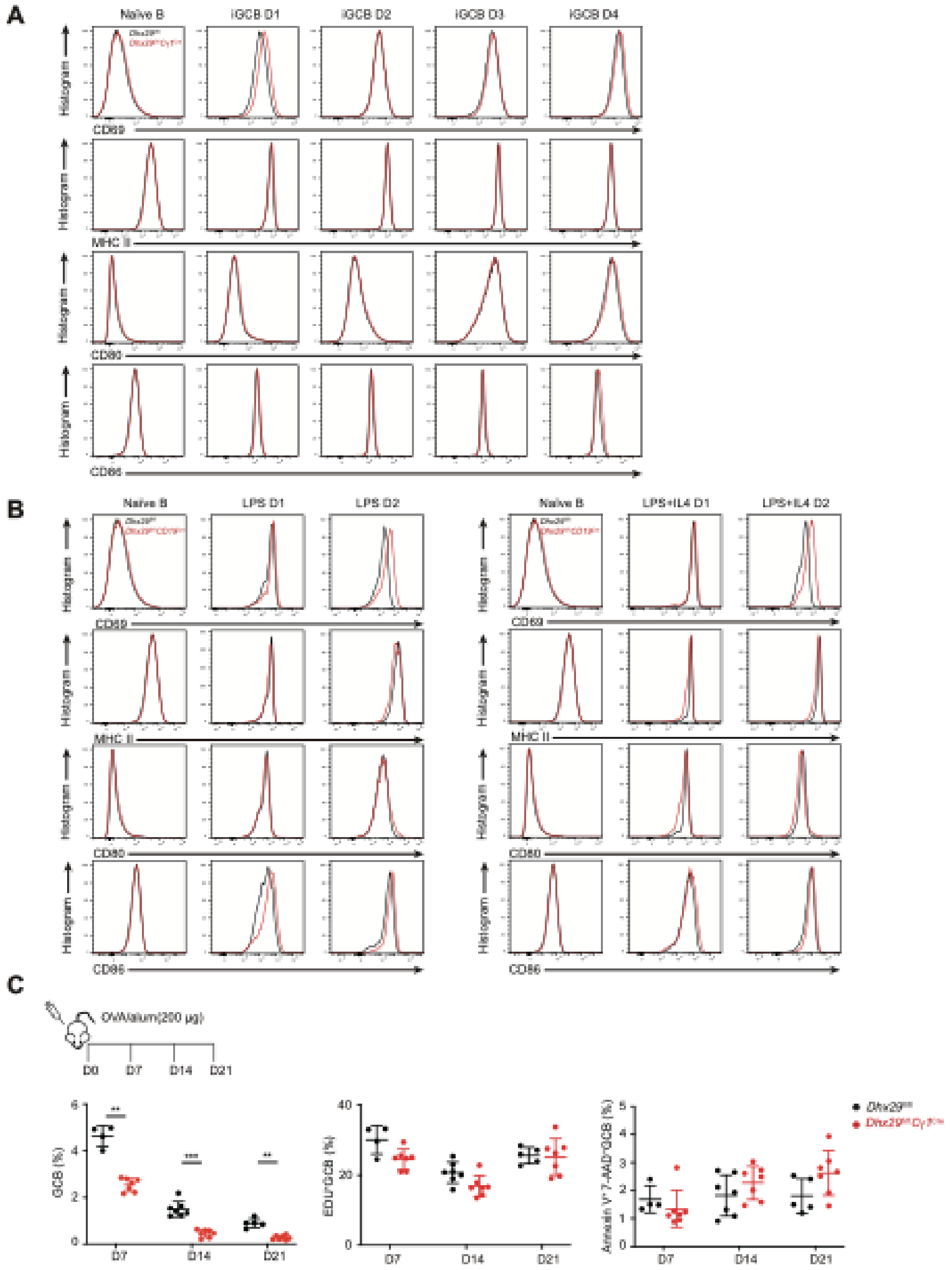

Reviewer Figure 1. The effect of Dhx29 deletion on B cell activation, establishment and maintenance of germinal centers.

(A-B) Flow cytometry analysis of activation marker expression on B cells in iGCB culture or stimulated with LPS or LPS plus IL4 for indicated amounts of time.

(C) Percentages of GCB ($GL7^{+}Fas^{+}$), EDU^{+} GCB and Annexin V^{+} $7\text{-}AAD^{+}$ GCB cells in the spleen of $Dhx29^{fl/fl}$ and $Dhx29^{fl/fl}C\gamma 1^{Cre}$ mice were determined by flow cytometry analysis at day 7, 14 and 21 post immunization with 200 μg OVA/alum (i. p). Each symbol represents an individual mouse. GCB cells (%) D7: P= 0.0021, D14: P= 0.0001, D21: P= 0. 0034.Small horizontal lines indicate the mean (± SD.). Statistical significance was determined by Two-way ANOVA in (C). $^{**}P < 0.01$, $^{***}P < 0.001$.

Using the Dhx29fl/fl Cg1CRE, it is key to assess timely gene deletion, loss of protein expression and class switching. In-vivo, the analysis should be done before and after establishment of the germinal center and, in-vitro, gene deletion couple to class-switching should be tested.

Response: As suggested by the reviewer, we have bred *Cγ1*^{Cre}; *Rosa*26$^{LSL\text{-}YFP}$ and *Dhx29*$^{fl/fl}$*Cγ1*^{Cre}; *Rosa*26$^{LSL\text{-}YFP}$ mice to examine Cre-mediated gene deletion and Dhx29 protein expression *in vivo*. In those mice, the expression of Cre recombinase, deletion of STOP cassette at the *Rosa*26$^{LSL\text{-}YFP}$ locus, and YFP expression is triggered in a very small number of antigen-specific B cells upon immunization. Those activated antigen-specific B cells would then proliferate and form germinal centers at day 4-5 post immunization. We were not able to obtain adequate number of YFP^{+} B cells for analysis at day 4 or 5 post immunization. Therefore, we sorted YFP^{+} B cells from both *Cγ1*^{Cre}; *Rosa*26$^{LSL\text{-}YFP}$ and *Dhx29*$^{fl/fl}$*Cγ1*^{Cre}; *Rosa*26$^{LSL\text{-}YFP}$ mice at day 7.5 post immunization with OVA/alum. As shown in Figure 6G, H of the revised manuscript, *Dhx29* mRNA was absent in YFP^{+} B cells from *Dhx29*$^{fl/fl}$*Cγ1*^{Cre}; *Rosa*26$^{LSL\text{-}YFP}$ mice, indicating a complete deletion of *Dhx29* gene and mRNA. The amount of Dhx29 protein was also significantly reduced in those cells (Figure 6G). In addition, we tracked gene deletion and Dhx29 protein expression in the *in vitro* culture system. As shown in Reviewer Figure 2, deletion of *Dhx29* gene started at day 1 and completed at day 2, while *Dhx29* mRNA was depleted at day 2. The depletion of Dhx29 protein started at day 2 and completed later. This is consistent with *in vivo* results: *Dhx29* gene deletion and mRNA depletion are fast, while Dhx29 protein depletion takes time.

We also examined class switch to IgG1 and IgE at various time points of iGCB culture. As shown in Reviewer Figure 3, the percentage and number of IgG1^{+} cells were notably reduced among *Dhx29*$^{fl/fl}$*Cg1*^{Cre} B cells at iPC day 1-4, accompanied by an increase in the percentage of IgE^{+} cells. However, the absolute numbers of IgE^{+} cells were comparable between WT and *Dhx29*$^{fl/fl}$*Cg1*^{Cre} B cells. Therefore, *Dhx29*-deficiency impaired class switch to IgG1, but not to IgE.

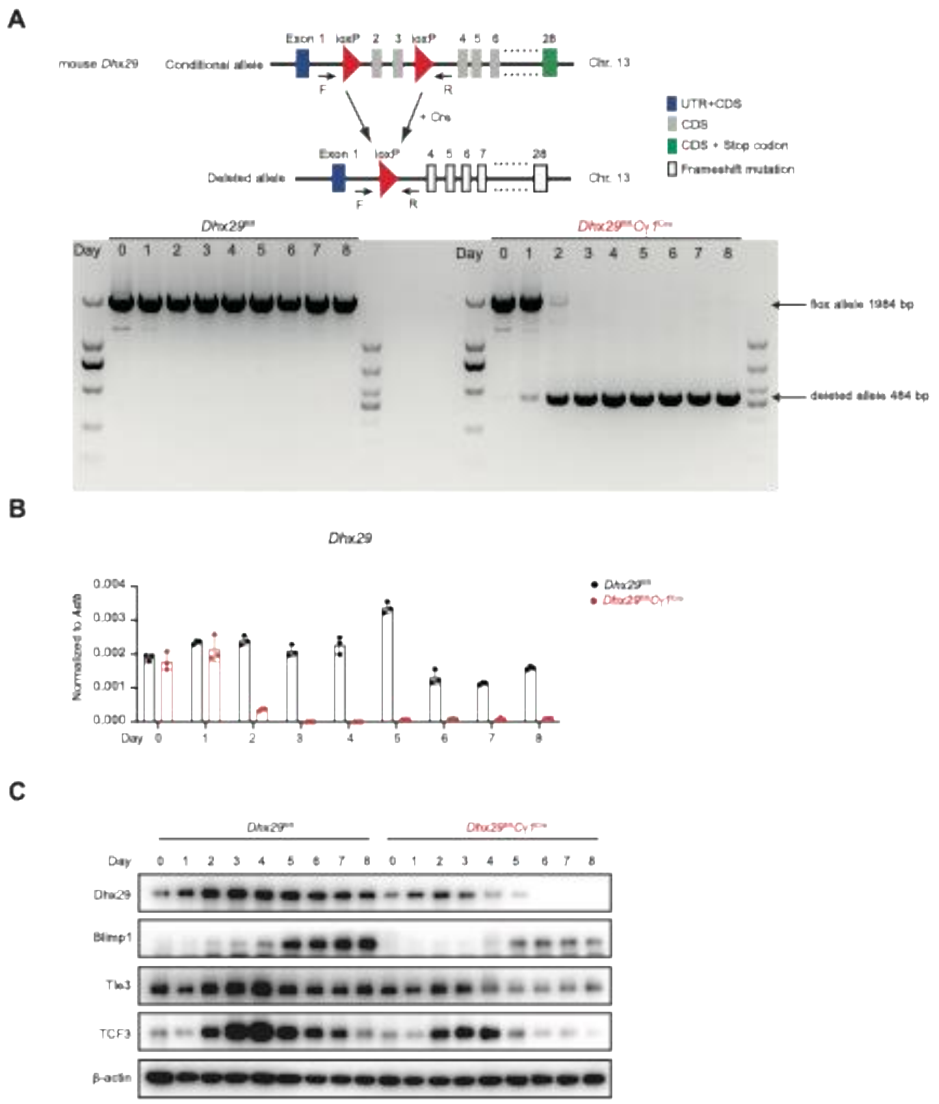

Reviewer Figure 2. ***Cγ1***$^{\mathbf{Cre}}$**-mediated deletion of *Dhx29* gene, mRNA, and protein.**

(**A**) Genotyping PCR to detect deleted and floxed Dhx29 alleles. Genomic DNA was extracted from *Dhx29*$^{fl/fl}$ and *Dhx29*$^{fl/fl}$*Cγ1*^{Cre} B cells during iGCB culture and used in genotyping PCR to evaluate the deletion efficiency of *Dhx29* allele.

(**B**) Quantitative RT-PCR analysis of *Dhx29* mRNA in *Dhx29*$^{fl/fl}$ and *Dhx29*$^{fl/fl}$*Cγ1*^{Cre} B cells at indicated time points. Data from three independent biological replicates.

(**C**) Immunoblot analysis of indicated proteins in *Dhx29*$^{fl/fl}$ and *Dhx29*$^{fl/fl}$*Cγ1*^{Cre} B cells during iGCB culture.

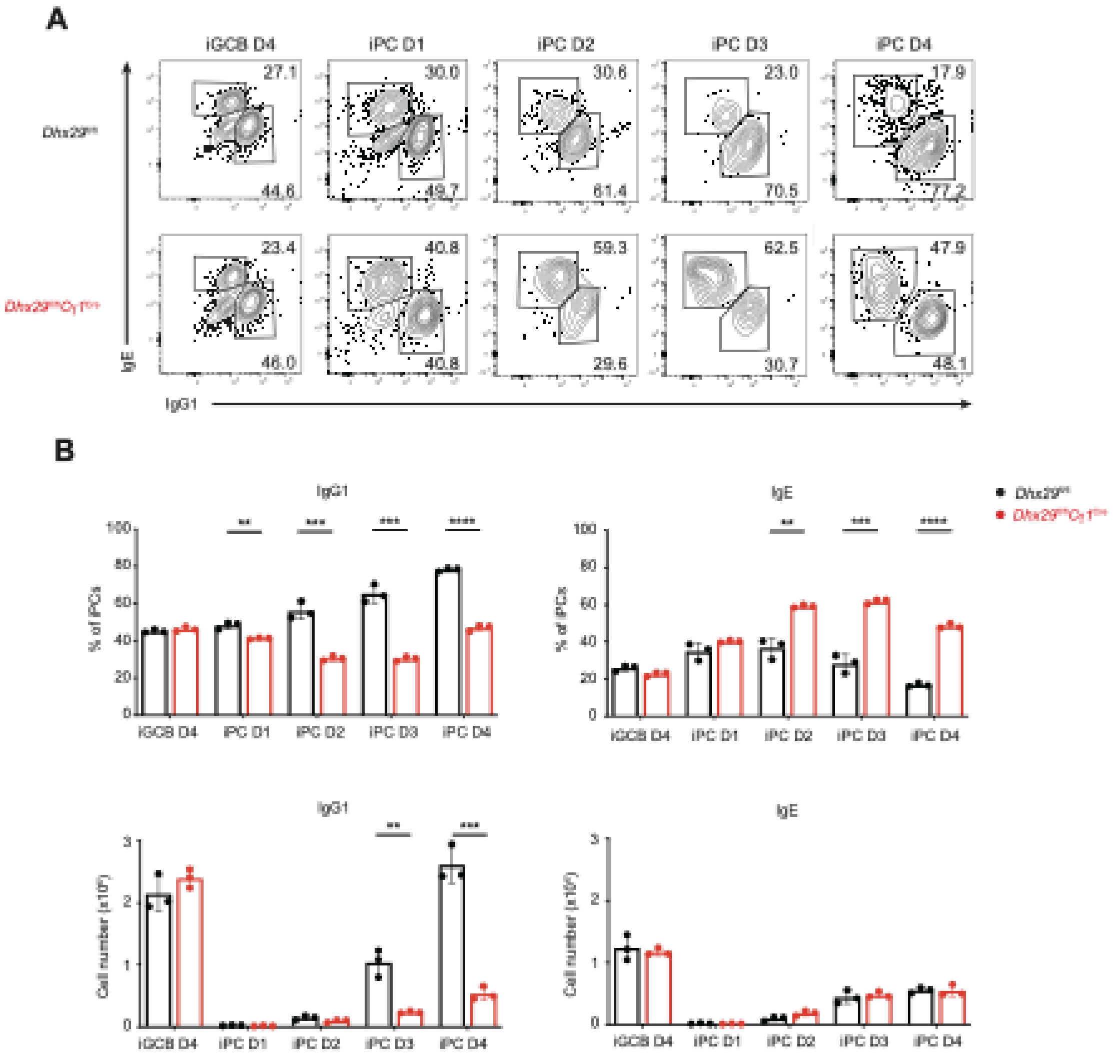

Reviewer Figure 3. Class switch to IgG1 and IgE of *Dhx29*$^{fl/fl}$*Cγ1*^{Cre} B cells.

(A) Flow cytometry analysis of IgG1^{+} and IgE^{+} cells among *Dhx29*$^{fl/fl}$ and *Dhx29*$^{fl/fl}$*Cγ1*^{Cre} B cells at indicated time points of iGCB culture.

(B) Bar graph summarizing the percentage (upper) and number (lower) of IgG1^{+} and IgE^{+} cells in (**A**). Data from three independent biological replicates (IgG1^{+} of iPCs (%): iPC D1 P= 0.0040, iPC D2 P= 0.0007, iPC D3 P= 0.0003, iPC D4 P= 9.46E-06; IgE^{+} of iPCs (%): iPC D2 P= 0.0017, iPC D3 P= 0.0003, iPC D4 P= 4.47E-06; IgG1^{+} cell number ($\times 10^{6}$): iPC D3 P= 0.0031, iPC D4 P= 0.0003). Small horizontal lines indicate the mean (± SD.). Statistical significance was determined by unpaired, two-tailed student's t-test. **P < 0.01, ***P < 0.001, ****P < 0.0001.

It is expected that GCs are constituted by a mixed of non-classed switched and class-switched GC B cells, antigen-specific and non-antigen specific GC B cells. Thus, further characterization of these germinal centers is needed to know whether establishment, maintenance, expansion/cycling of class-switched and/or antigen-specific GC cells and/or differentiation of GC B cells is affected.

Response: As shown in Reviewer Figure 1, establishment of germinal centers was impaired, while their maintenance was likely normal. To characterize antigen-specific GCB cells, we immunized *Dhx29*$^{fl/fl}$ and *Dhx29*$^{fl/fl}$*Cg1*^{Cre} mice with NP-OVA/alum. As B cells mainly switch to IgG1 in this system, we quantified total IgG1^{+} and NP-specific IgG1^{+} GCB cells at day 14 post immunization.

As shown in Reviewer Figure 4, both the frequency and absolute number of total IgG1$^+$ and NP-specific IgG1$^+$ GCB cells were reduced among the splenocytes of *Dhx29*$^{fl/fl}$*Cg1*^{Cre} mice, indicating impaired class switch to IgG1. We also identified a discrete population of NP-specific IgM$^+$ GCB cells, whose frequency and absolute number were also profoundly decreased in *Dhx29*$^{fl/fl}$*Cg1*^{Cre} mice, a finding consistent with the drop in NP-specific IgM titers (Figure 3A of the revised manuscript).

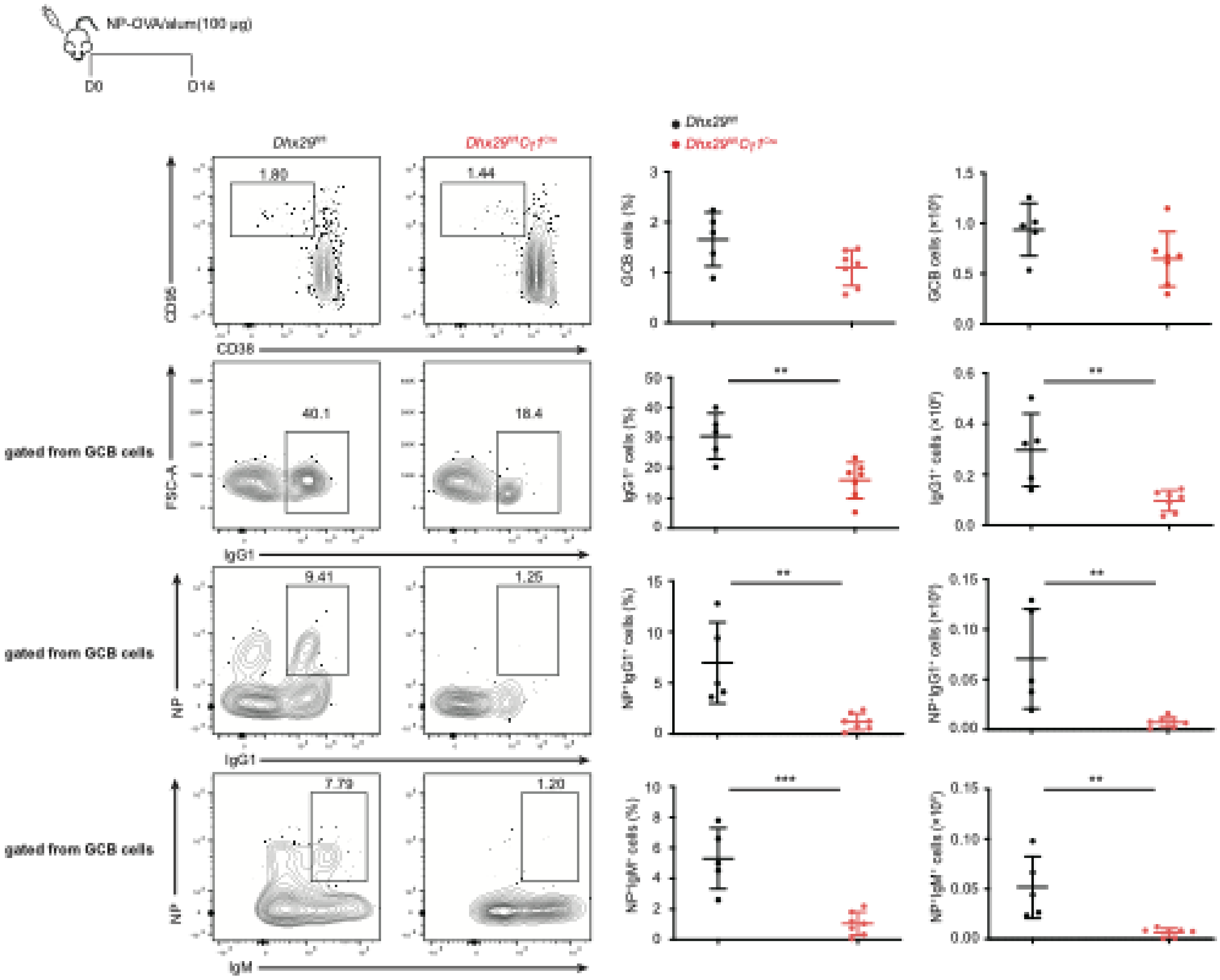

Reviewer Figure 4. Decreased class-switched and/or antigen-specific GC cells in *Dhx29*$^{fl/fl}$*Cγ1*^{Cre} mice

Flow cytometry analysis of GCB, IgG1$^+$ GCB, NP-specific IgG1$^+$ GCB and NP-specific IgM$^+$ GCB cells in the spleen of *Dhx29*$^{fl/fl}$ and *Dhx29*$^{fl/fl}$*Cγ1*^{Cre} mice at 14 days post immunization with NP-OVA/alum (i.p). Dot plots summarizing the percentage and number of GCB, IgG1$^+$ GCB, NP-specific IgG1$^+$ GCB and NP-specific IgM$^+$ GCB cells. IgG1$^+$ cells (%): P= 0.0037, IgG1$^+$ cells ($\times 10^6$): P= 0.0052; NP$^+$IgG1$^+$ cells (%): P= 0.0034, NP$^+$IgG1$^+$ cells ($\times 10^6$): P= 0.0066; NP$^+$IgM$^+$ cells (%): P= 0.0004, NP$^+$IgM$^+$ cells ($\times 10^6$): P= 0.0029. Each symbol represents an individual mouse. Small horizontal lines indicate the mean (± SD.). Statistical significance was determined by unpaired, two-tailed student's t-test. **P < 0.01, ***P < 0.001.

Both competitive and non-competitive settings are required to assess the contribution of protein-sufficient and protein-depleted B cells.

Response: We have generated mixed bone marrow chimeras by reconstituting lethally irradiated *Rag1*$^{-/-}$ mice with wild type CD45.1^{+} and *Cγ1*^{Cre} or *Dhx29*$^{fl/fl}$*Cγ1*^{Cre} (CD45.2^{+}) bone marrow cells at a 50:50 ratio. Twelve weeks after reconstitution, those chimeras were immunized with OVA/alum and analyzed by flow cytometry 7 days later. The frequencies of GCB and plasma cells derived from *Dhx29*$^{fl/fl}$*Cγ1*^{Cre} (CD45.2^{+}) B cells were much lower than those from WT (CD45.1^{+}) and *Cγ1*^{Cre} (CD45.2^{+}) B cells, demonstrating a cell-intrinsic role of Dhx29 in germinal center B cell formation and plasma cell differentiation. Those results are now included in Figure 2F, G of the revised manuscript.

2. Following this argument, the authors should explain why NP-IgM titres are reduced in the Dhx29fl/fl Cγ1CRE mice. Presumably, IgM+ GC B cells and plasma cells should still express DHX29. Similarly, authors should explain which is the percentage of cells that undergo class-switching from IgM to IgG1 or IgE at all different stages of their culture. Also, they should explain why IgE+ iGC B cells are expanded in their in-vitro settings as this normally occurs after class-switch recombination to IgG1. Again, timely analysis of protein depletion in Dhx29fl/fl Cγ1CRE cells is needed.

Response: We appreciate the referee's thoughtful comments. As reported in the original *Cγ1*^{Cre} paper (Casola *et al*, 2006)，B cell activation and subsequent germline Cγ1 transcription was sufficient to induce Cre expression and Cre-mediated recombination. They also found EYFP^{+}IgM^{+} B cells which lacked GC B cell markers in unimmunized *Cγ1*^{Cre}; *R26-EYFP* mice, suggesting class switch to IgG1 is not a precondition for Cre expression in *Cγ1*^{Cre} mice. Therefore, upon NP-OVA/alum immunization of *Dhx29*$^{fl/fl}$*Cγ1*^{Cre} mice, germline Cγ1 transcription and Cre expression occurred in certain activated IgM^{+} B cells that did not undergo class switch to IgG1. Cre-mediated deletion of Dhx29 in those cells impaired their differentiation into GCB and plasma cells (Reviewer Figure 4), resulting in reduced NP-IgM titers (Figure 3A of the revised manuscript).

We have now examined class switch to IgG1 and IgE at various time points of iGCB culture. As shown in Reviewer Figure 3, the percentage and number of IgG1^{+} cells were notably reduced among *Dhx29*$^{fl/fl}$*Cg1*^{Cre} B cells at iPC day 1-4, accompanied by an increase in the percentage of IgE^{+} cells. However, the absolute numbers of IgE^{+} cells were comparable between WT and *Dhx29*$^{fl/fl}$*Cg1*^{Cre} B cells. Therefore, *Dhx29*-deficiency impaired class switch to IgG1, but not to IgE.

We have tracked gene deletion and Dhx29 protein expression at various time points of iGCB culture. As shown in Reviewer Figure 2, deletion of *Dhx29* gene started at day 1 and completed at day 2, while *Dhx29* mRNA was depleted at day 2. The depletion of Dhx29 protein started at day 2 and completed at day 3-4.

3. A significant body of the manuscript reports alterations in B-cell development using Dhx29fl/fl CD19CRE and Dhx29fl/fl Mb1CRE mice without providing further explanation. The RNA targets and mechanisms regulated by Dhx29 are likely very different to those reported in activated B cells and GCs. Thus, these models are largely irrelevant to later.

Response: We appreciate the insightful comments of the reviewer. Conditional deletion of *Dhx29* at different stages of B cell development resulted in distinct phenotypes, suggesting that Dhx29 plays

different roles at different stages of B cell development and differentiation. We agree with the reviewer that the RNA targets and mechanisms regulated by Dhx29 are likely to be cellular context-dependent. Those are interesting scientific questions warranting further investigation. We have now moved those phenotypic findings to the Appendix section of the revised manuscript, mainly for the purpose of information sharing. Hopefully, those results will stimulate the interests of other scientists studying B cell biology and/or Dhx29 and provide a foundation for them to initiate their investigation.

4. Protein expression data should complement quantitation of Dhx29 mRNA abundance in figure 2A, 2B and 2C. Authors should attempt to explain the differences in Dhx29 mRNA abundance between in-vitro activated B cells and GC B cells. This is important as different immunoblotting data show that Dhx29 is not detected in naïve follicular B cells. Thus, is it expressed in GC B cells at the protein level?

Response: As suggested by the reviewer, we have now examined Dhx29 protein expression and included the results in the revised manuscript (Fig 2A-D and Appendix Fig. S1B). Dhx29 mRNA and protein exhibit different expression patterns in Fr. B-E, the development stages when bone marrow B cell precursors enter a phase of active translation and rapid proliferation, suggesting post-transcriptional regulation of Dhx29 expression. As shown in Figure 2D of the revised manuscript, Dhx29 protein expression was higher in GCB cells than naïve follicular B cells, while the highest expression was found in plasma cells. GCB cells sorted from immunized mice differ significantly from *in vitro* activated B cells, which may underlie the differences in *Dhx29* mRNA and protein expression between those cells.

5. One critical aspect is that apparently, BLIMP1 expression is defective from as soon as the iGCB d4 stage. However, the percentage of CD138+ cells is similar until iPC d6 without further explanation. Consistent measurement of CD138+ or CD138- and IgG1+ or IgG1- cell cycle progression (in fig. EV6A, cells in S phase varies from 80% to 10% without further explanation why), cell expansion and survival should be performed.

Response: The reviewer raised a critical point. We have reproducibly found that Blimp1 expression is defective at iGCB day 4, while the difference in the percentage of $CD138^+$ cells does not manifest until iPC day 3 (Figure 5E and Figure EV2B of the revised manuscript). A previous study reported the presence of Blimp1-independent initiation of plasma cell differentiation and the presence of $CD138^+CD19^+$ cells in *Prdm1*$^{gfp/gfp}$ mice, which harbor a *Prdm1* locus with *gfp knock-in*, resulting in GFP expression under the control of the *Prdm1* locus and production of a truncated and functionless Blimp1 protein (Kallies *et al*, 2007). We speculate that the generation of $CD138^+$ cells at iPC day 1 and 2 is Blimp1-independent and those cells are counterparts of $CD138^+CD19^+$ cells in *Prdm1*$^{gfp/gfp}$ mice.

As recommended by the reviewer, we have now performed quantification of cell cycle progression and survival of various cell subsets in iPC culture, including *$CD138^+$ or $CD138^-$ and $IgG1^+$ or $IgG1^-$* cells (Reviewer Figure 5). The proliferation rate of iPCs at day 1 and 2 is higher than iPCs at day 3 and 4, likely due to the presence of fresh medium and cytokine (IL-21) at the beginning of iPC

culture. We speculate that both cytokine and nutrients have been consumed at the later stage of iPC culture (i.e. day 3 and 4), resulting in much reduced proliferation rate.

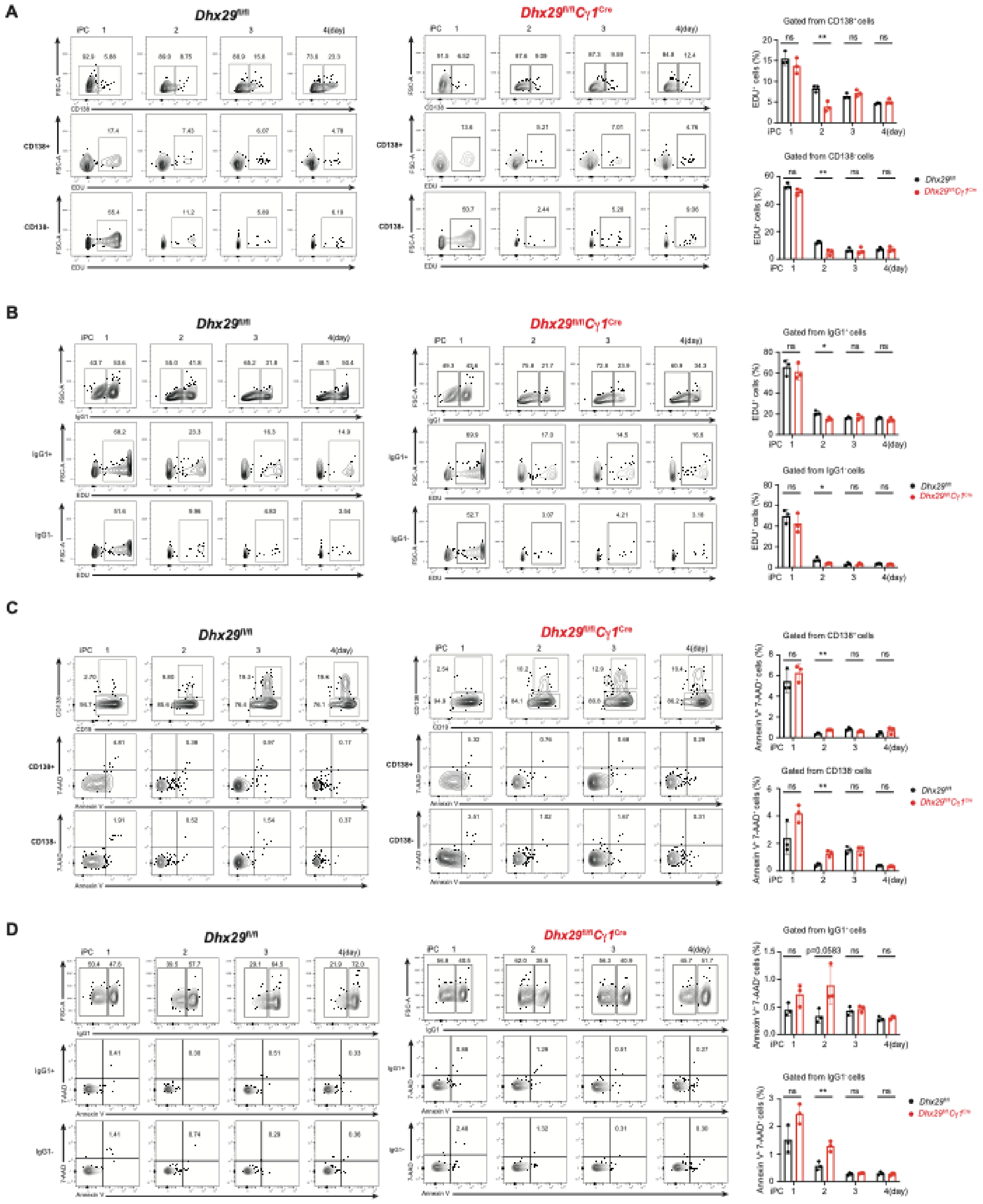

Reviewer Figure 5. Proliferation, expansion and survival of *$Dhx29^{fl/fl}$* and *$Dhx29^{fl/fl}C\gamma1^{Cre}$* B cells during iPC culture.

(A) Flow cytometry analysis of EDU-based measurement of cell proliferation of CD138^{+} and CD138^{-} cells among *Dhx29*$^{fl/fl}$ and *Dhx29*$^{fl/fl}$*Cγ1*^{Cre} B cells during iPC culture. Bar graph summarizing the percentage of EDU^{+}CD138^{+} and EDU^{+}CD138^{-} cells in (**A**). Data from three independent biological replicates (EDU^{+}CD138^{+} cells (%): iPC D2 P= 0.0064; EDU^{+}CD138^{-} cells (%): iPC D2 P= 0.0054).
(B) Flow cytometry analysis of EDU-based measurement of cell proliferation of IgG1^{+} and IgG1^{-} cells among *Dhx29*$^{fl/fl}$ and *Dhx29*$^{fl/fl}$*Cγ1*^{Cre} B cells during iPC culture. Bar graph summarizing the percentage of EDU^{+} IgG1^{+} and EDU^{+} IgG1^{-} cells. Data from three independent biological replicates (EDU^{+} IgG1^{+} cells (%): iPC D2 P= 0.0193; EDU^{+} IgG1^{-} cells (%): iPC D2 P= 0.0408).
(C) Flow cytometry analysis of Annexin V^{+}7-AAD-based measurement of survival of CD138^{+} and CD138^{-} cells among *Dhx29*$^{fl/fl}$ and *Dhx29*$^{fl/fl}$*Cγ1*^{Cre} B cells during iPC culture. Bar graph summarizing the percentage of Annexin V^{+}7-AAD^{+}CD138^{+} and Annexin V^{+}7-AAD $^{+}$CD138^{-} cells. Data from three independent biological replicates (Annexin V^{+}7-AAD $^{+}$CD138^{+} cells (%): iPC D2 P= 0.0014; Annexin V^{+}7-AAD $^{+}$CD138^{-} cells (%): iPC D2 P= 0.0053).
(D) Flow cytometry analysis of Annexin V^{+}7-AAD-based measurement of survival of IgG1^{+} and IgG1^{-} cells among *Dhx29*$^{fl/fl}$ and *Dhx29*$^{fl/fl}$*Cγ1*^{Cre} B cells during iPC culture. Bar graph summarizing the percentage of Annexin V^{+}7-AAD^{+} IgG1^{+} and Annexin V^{+}7-AAD^{+} IgG1^{-} cells. Data from three independent biological replicates (Annexin V^{+}7-AAD $^{+}$ IgG1^{+} cells (%): iPC D2 P= 0.0583; Annexin V^{+}7-AAD $^{+}$ IgG1^{-} cells (%): iPC D2 P= 0.0072).
Small horizontal lines indicate the mean (± SD.). Statistical significance was determined by unpaired, two-tailed student's t-test. *P < 0.05, **P < 0.001.

6. In Figure 4, authors focus their attention on BLIMP1 without further explanation. Transcriptomics analyses are done at iPC 0, 12 and 24 hours, but no attempt to assess common or different alteration in mRNA abundance is done. Is Prdm1 altered at all time points?

Response: As suggested by the reviewer, we have now re-analyzed the RNA-seq data of 0, 12 and 24 hour iPCs. Principal component analysis (PCA) of the transcriptomes and Venn plots show that the number of differentially expressed genes gradually increased from 0 to 24 h, and that *Prdm1* expression was altered at all time points (Reviewer Figure 6).

Are there other relevant targets being identified?

Response: Xbp1 and Eaf2, two downstream target genes of Blimp1 (encoded by *Prdm1*), are also among differentially expressed genes. Xbp1 controls unfolded protein response, drives ER expansion, and induces the expression of many genes involved in ER protein homeostasis. Eaf2 is a critical activator of Ell2, which promotes choice of the distal *Igh* polyadenylation site and generation of *Igh* mRNA encoding the secreted antibody.

Are Tle3 and Tcf3 altered at any time point?

Response: We have now examined the mRNA and protein levels of *Prdm1*, *Tle3* and *Tcf3* throughout iGCB culture. While decrease in Prdm1 mRNA and protein expression started at iGCB day 4 and continued afterwards (Figure 5C, E of the revised manuscript), *Tle3* and *Tcf3* mRNA expression remained the same in WT and *Dhx29*$^{fl/fl}$*Cγ1*^{Cre} B cells. Meanwhile, the protein levels of

Tle3 and TCF3 began to decline at iGCB day 2-3, immediately following the decrease in Dhx29 protein (Figure 6E, F of the revised manuscript). This is consistent with our working model that Dhx29 controls the translation of *Tle3* and *TCF3* mRNAs, while Tle3 and TCF3 promote *Prdm1* transcription and subsequent protein production.

Are any of the genes identified in iGC or iPC cells differentially expressed in-vivo?

Response: We have now sorted YFP^{+} cells from immunized *Cg1*^{Cre}; *Rosa26*$^{LSL-YFP}$ and *Dhx29*$^{fl/fl}$*Cg1*^{Cre}; *Rosa26*$^{LSL-YFP}$ mice and examined Tle3 and TCF3 mRNA and protein expression. Consistent with *in vitro* data, *Dhx29*-deficiency led to substantial reduction in Tle3 and TCF3 protein expression, without decreasing their mRNA levels (Figure 6G, H of the revised manuscript).

And which is their functional relevance for the GC response and differentiation of PCs?

Response: TCF3 functions as a transcription factor and plays an indispensable role in GCB formation and plasma cell differentiation. By regulating 3′ Igk and Igh enhancers and a distal element at the *Prdm1* locus, TCF3 controls CSR to IgG1 in plasmablasts (Gloury *et al*, 2016; Wohner *et al*, 2016; Wu *et al*, 2022). The role of Tle3 in germinal center response and plasma cell differentiation was incompletely understood. Our study shows that Tle3 binds to the promoter region of *Prdm1* and controls its transcriptional activity (Figure 7 of the revised manuscript). In addition, sgRNA-mediated knockout of *Tle3* impaired plasma cell differentiation, while retroviral expression of Tle3 restored plasma cell differentiation of *Dhx29*$^{fl/fl}$*Cg1*^{Cre} B cells (Figure 6D and I of the revised manuscript). Therefore, both TCF3 and Tle3 are functional targets of Dhx29.

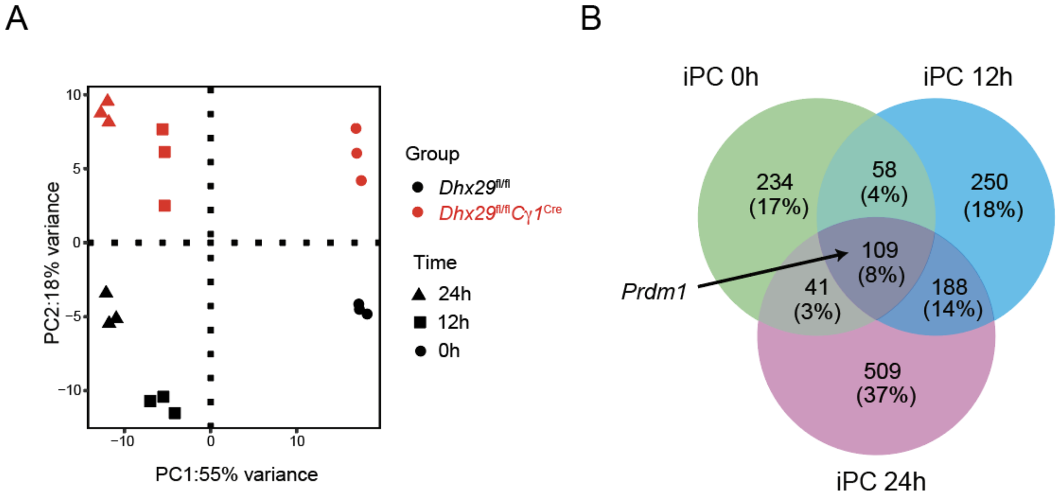

Reviewer Figure 6. Principal component analysis (PCA) and Venn plot analysis of differentially expressed genes in WT and *Dhx29*$^{fl/fl}$*Cγ1*^{Cre} iPCs.

(A) Principal component analysis (PCA) of transcriptomes of *Dhx29*$^{fl/fl}$ and *Dhx29*$^{fl/fl}$*Cγ1*^{Cre} B cells at iPC 0h, 12h and 24h.

(B) Venn plots showing the numbers and percentages of differentially expressed genes at iPC 0h, 12h and 24h.

7. Authors move to iGCB d3.5 to integrate transcriptomics, translatomics and proteomics. First,

information about whether the RNAseq data is obtained from the same cells used for ribosome profiling is not indicated.

Response: Yes, RNA-seq data is obtained from the same cells used for ribosome profiling.

Second, there is no information provided about the quality and reproducibility of these assays.

Response: We have now included the quality and reproducibility of these assays in Appendix section.

Third, the rational to focus the attention on Tle3 and Tcf3 is not clear.

Response: In this study, we found that the mRNA and protein levels of *Prdm1*, the master regulator of plasma cell differentiation, were markedly reduced in *Dhx29*-deficient B cells. As Dhx29 is an RNA helicase that resides in the cytosol and promotes translation of mRNAs with structured 5'UTRs, we speculate that: (1) Dhx29 directly controls translation of its target genes; (2) these target genes encode proteins that promote *Prdm1* transcription directly or indirectly; (3) these target genes should exhibit: a. reduced protein levels; b. reduced translation efficiency; and c. unaltered mRNA levels in *Dhx29*-deficient B cells. We therefore performed mass spectrometry (MS), RNA-seq, and Ribo-seq analysis of *Dhx29*$^{fl/fl}$ and *Dhx29*$^{fl/fl}$*Cγ1*^{Cre} B cells to identify genes meeting those criteria. MS analysis identified 153 proteins downregulated in *Dhx29*$^{fl/fl}$*Cγ1*^{Cre} B cells. Among those 153 downregulated proteins, RNA-seq and Ribo-seq analyses identified 13 with reduced translation efficiency but unaltered mRNA levels. We next performed CRISPR/Cas9-mediated functional analysis of those 13 genes to identify the ones regulating plasma cell differentiation. Knockout of TCF3 and Tle3 showed effect similar to, but milder than, Dhx29 knockout in terms of impaired plasma cell differentiation and reduced Blimp1 expression. Importantly, retroviral expression of TCF3 isoforms (E12 and E47) and Tle3 restored Blimp1 expression and plasma cell differentiation of Dhx29-deficient B cells, suggesting that TCF3 and Tle3 are functional target genes of Dhx29.

Fourth, no attempts are made to validate TCF3 as a key transcriptional factor regulating Prdm1.

Response: We have previously reported that TCF3 binds to an upstream element of the *Prdm1* genomic locus and promotes *Prdm1* transcription (Wu *et al.*, 2022). We have cited this publication in our manuscript.

8. It remains unclear by which mechanism TCF3 and TLE3 controls Prdm1 gene transcription. Luciferase assays are provided for TLE3 but no relevant experimental validation is done in iGCB cells and in primary GCB cells.

Response: We have previously reported that TCF3 binds to an upstream element of the *Prdm1* genomic locus and promotes *Prdm1* transcription. We therefore focus on elucidating the mechanism underlying Tle3 regulation of *Prdm1* transcription. CUT&Tag followed by PCR showed that Tle3 binds to the *Prdm1* promoter region, while luciferase reporter assay showed that Tle3 promotes the transcriptional activity of this Tle3 binding sequence. These results suggest that Tle3 binds to the

promoter of *Prdm1* and facilitates its transcription. We have now included those data in Figure 7 of the revised manuscript.

We have performed luciferase assays for Tle3 in iGCB cells and the results are now included in Figure 7D of the revised manuscript. Due to the relatively small number of GCB cells from immunized mice, we were not able to obtain sufficient amounts of primary GCB cells for luciferase assays.

9.Polysome profiling assays are little informative without properly testing reproducibility and quantification of binding to the 40S ribosome.

Response: As shown in Figure 8A of the revised manuscript, mRNA distribution of β-actin, TCF3 and Tle3 in polysome fractions were pooled from three independent experiments, demonstrating high reproducibility of this assay. Reviewer Figure 7 shows polysome profiles of two independent experiments. In this experiment, we employed a quantitative approach in which equal numbers of *Dhx29*$^{fl/fl}$ and *Dhx29*$^{fl/fl}$*Cγ1*^{Cre} B cells were harvested after CHX treatment, and equal amount of RNA were loaded onto the sucrose gradient prior to ultracentrifugation. These conditions were controlled to minimize experimental variations.

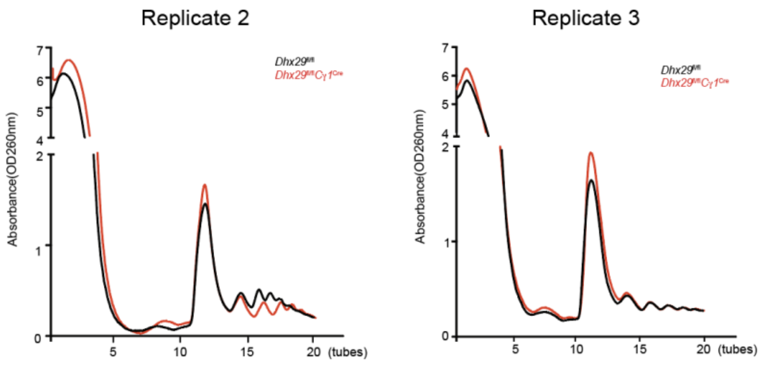

Reviewer Figure 7. Polysome profiling analysis of *Dhx29*$^{fl/fl}$ and *Dhx29*$^{fl/fl}$*Cγ1*^{Cre} B cells. Polysome profiles of *Dhx29*$^{fl/fl}$ and *Dhx29*$^{fl/fl}$*Cγ1*^{Cre} B cells at iGCB day 3.5 from two independent experiments.

10. The direct binding of Dhx29 to the 5'UTR of Tcf3 and Tle3 mRNAs is not formally proven. Methods such as cross-linking immunoprecipitation and sequencing are capable to identify this type of interactions, with identification of the putative elements and nucleotides bound by the protein for later biochemical validation (e.g., mutation of binding sites). Analysis of Dhx29 to relevant mRNA targets in primary GC B cells should be attempted.

Response: We appreciate the insightful comments and suggestion of the reviewer. We were not able to perform eCLIP experiments to corroborate direct binding of Dhx29 to the 5'UTR of TCF3 and Tle3 mRNAs due to the lack of high-quality antibodies able to pull down endogenous Dhx29 protein from primary B cells. Instead, we have performed RIP assay and biotin-labelled RNA pull-down assay, and showed that Dhx29 bound to 5’ UTR of TCF3 and Tle3 mRNAs, while Dhx29-RBDmut, which lacks the dsRNA-binding domain, exhibited much reduced binding. In

addition, we have also performed electrophoretic mobility shift assay (EMSA), which showed that recombinant wild type Dhx29 protein directly interacted with both 5' UTRs and that those interactions were attenuated after deletion of Dhx29 dsRNA-binding domain. Those results are now included in Figure 8D-F of the revised manuscript.

11. Rescue of phenotypes is done on iGC cell cultures. However, these should be attempted in-vivo in the mouse model.

Response: We appreciate the suggestion of the reviewer. We have bred *Dhx29*$^{fl/fl}$*Cγ1*^{Cre}; B1-8 ^{hi} mice during the period of manuscript revision. B cells from B1-8^{hi} and *Dhx29*$^{fl/fl}$*Cγ1*^{Cre}; B1-8^{hi} mice were transduced with retroviruses encoding *Dhx29*, *Tcf3* or *Tle3*, and co-transferred with OVA-specific OT-II CD4^{+} T cells into CD45.1^{+} wild type recipient mice, followed by NP-OVA/alum immunization. As shown in Reviewer Figure 8, we assessed both cell viability and transduction efficiency (i.e. GFP^{+} cells) before transfer. While GFP^{+} B1-8^{hi} B cells were easily detectable after NP-OVA/alum immunization, we were not able to detect any GFP^{+} *Dhx29*$^{fl/fl}$*Cγ1*^{Cre}; B1-8^{hi} B cells after immunization. Those results are perplexing. We speculate that our *Dhx29*$^{fl/fl}$*Cγ1*^{Cre}; B1-8^{hi} mice are not on pure C57BL/6 genetic background and this may lead to rejection of retrovirally transduced *Dhx29*$^{fl/fl}$*Cγ1*^{Cre}; B1-8^{hi} B cells by recipient mice.

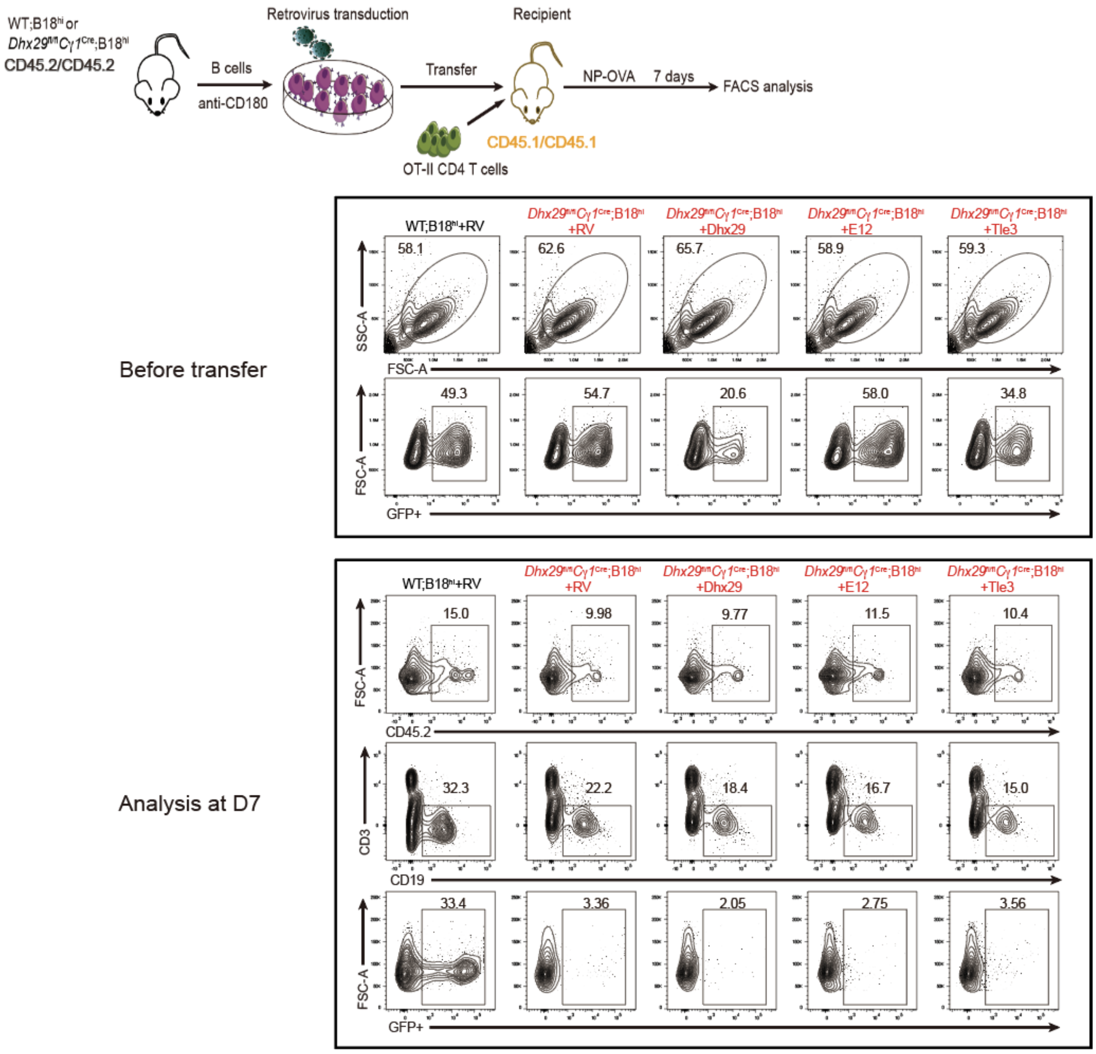

Reviewer Figure 8. Adoptive transfer of B1-8^{hi} B cells into CD45.1^{+} wild type recipient mice.

5×10^6 B cells from B1-8[hi] and *Dhx29*[fl/fl]*Cγ1*[Cre]; B1-8[hi] mice were transduced with retroviruses encoding vector, *Dhx29*, *Tcf3* or *Tle3*, transferred intravenously (i.v.) into CD45.1$^+$ wild type recipient mice together with 5×10^5 OVA-specific OT-II CD4$^+$ T cells. Twelve hours later, recipient mice were immunized i.p. (intraperitoneal injection) with 100 μg NP-OVA/alum and analyzed 7 days post immunization. Cell viability and transduction efficiency (indicated by GFP expression) were examined before transfer. GFP$^+$ cells among CD45.2$^+$ B cells were examined after immunization.

12. In general, methods and figure legends are poorly described across the manuscript. There is a lack of clarity on whether authors are representing technical replicates of biological replicates in each of their in-vitro experiments. Attempts to pool together or represent data from independent experiments is desirable to assess reproducibility. The type of statistical test should be indicated for each figure. Data quality analyses for each of the different omics approaches used across the manuscript should be presented.

Response: As the reviewer suggested, we have revised the methods and figure legends, explicitly stating the types of replicates and the statistical tests used in each figure. In addition, a comprehensive data-quality assessment for each omics experiment is now provided in the Appendix section.

Referee #2:

The study of Zhao and colleagues investigates a role Dhx29 in humoral immunity. The authors used a boutique CRIPSR/Cas9 screen to identify RNA helicases involved in vitro B cell differentiation. Dhx29 was identified as being more essential for plasma cell differentiation comparted to B cell proliferation/ survival. The authors then made multiple B cell specific conditional KOs of Dhx29 and showed that it is important for the GC response, plasma cell differentiation and antibody production. As Dhx29 was thought to control mRNA translation, the authors performed quantitative mass spectrometry and then searched for proteins that were reduced in the Dhk29 KO while the mRNA level was unchanged. They ultimately identified TCF3 and TLE3 as being key targets of Dhx29 controlling plasma cell differentiation, potentially through the regulation of Prdm1/Blimp1.

Overall, this is a very nicely conducted study that addresses and interesting and understudied area of B cell biology. The authors have developed excellent mouse models to address the function of Dhx29, the figures are clear, and the manuscript well written.

I have the following concerns that should be addressed.

Major comments

1. Figure 2 DHX29 expression. The data in panel A and C suggest reasonably dynamic expression of Dhx29 mRNA. However, the expression values shown are not consistent with databases such as Immgen and the authors own protein data (Figure 4E) that shows no Dhx29 protein in naïve B cells and the expression in in vitro PCs is actually lower than activated B cells. I suggest additional analysis protein (by western blotting or intracellular FACS) of in some ex vivo B cell populations, particularly those populations panel C with bring some more clarity to this issue.

Response: We appreciate this insightful suggestion of the reviewer. As our qPCR-based mRNA measurements did not align with the RNA-seq data of Immgen, we have now examined Dhx29 protein levels by immunoblot. As shown in Appendix Figure S1B, C of the revised manuscript, Dhx29 mRNA and protein exhibit different expression patterns in Fr. B-E, the development stages when bone marrow B cell precursors enter a phase of active translation and rapid proliferation, suggesting post-transcriptional regulation of Dhx29 expression.

As shown in Figure 2D of the revised manuscript, Dhx29 protein expression in GCB cells was slightly higher than naïve follicular B cells, while the highest expression was found in plasma cells. GCB cells were sorted from immunized mice at day 7 post immunization and they may differ significantly from those activated *in vitro* for 24 or 48 hrs by various stimuli (Figure 2B and D), which may underlie the differences in *Dhx29* mRNA and protein abundance between those cells.

2. Figure 3. The authors conclude that Dhx29 is indispensable for IgG1 class switch recombination. However, they are measuring the process only during the PC differentiation phase of the culture. As the CSR occurs prior to PC differentiation and requires IL4, then the experiments shown in fig 4G-H needs to be conducted in the iGCB phase, presumably day 3-4 when Dhx29 protein is absent. Finally, have the IgM+ cells been excluded from panel G ?

Response: The reviewer raised a critical point. As shown in Reviewer Figure 9, we have examined class switching to IgG1 and IgE in WT and *Dhx29*$^{fl/fl}$*Cg1*^{Cre} B cells at every single day between

iGCB day 4 and iPC day 4. As shown in Reviewer Figure 9A, B, iGCB day 4 WT and *Dhx29*$^{fl/fl}$*Cg1*^{Cre} B cells exhibited similar class switching to IgG1 and IgE, while the percentage and number of IgG1^{+} cells were significantly reduced in *Dhx29*$^{fl/fl}$*Cg1*^{Cre} B cells starting from iPC day 1.

We also examined germline transcription of IgG1, which should precede class switching to IgG1. Indeed, *Dhx29*$^{fl/fl}$*Cg1*^{Cre} B cells showed a slight decrease in germline transcription of IgG1 at iGCB day 4 and the difference became more pronounced at iPC day 1 (Reviewer Figure 9C). As the reviewer pointed out, reduced germline transcription of and class switching to IgG1 occurred prior to plasma cell differentiation.

As shown in Reviewer Figure 9A, cells were gated from total B cells. Due to robust cytokine stimulation in this system, class-switching has occurred in almost all B cells by iPC day 2.

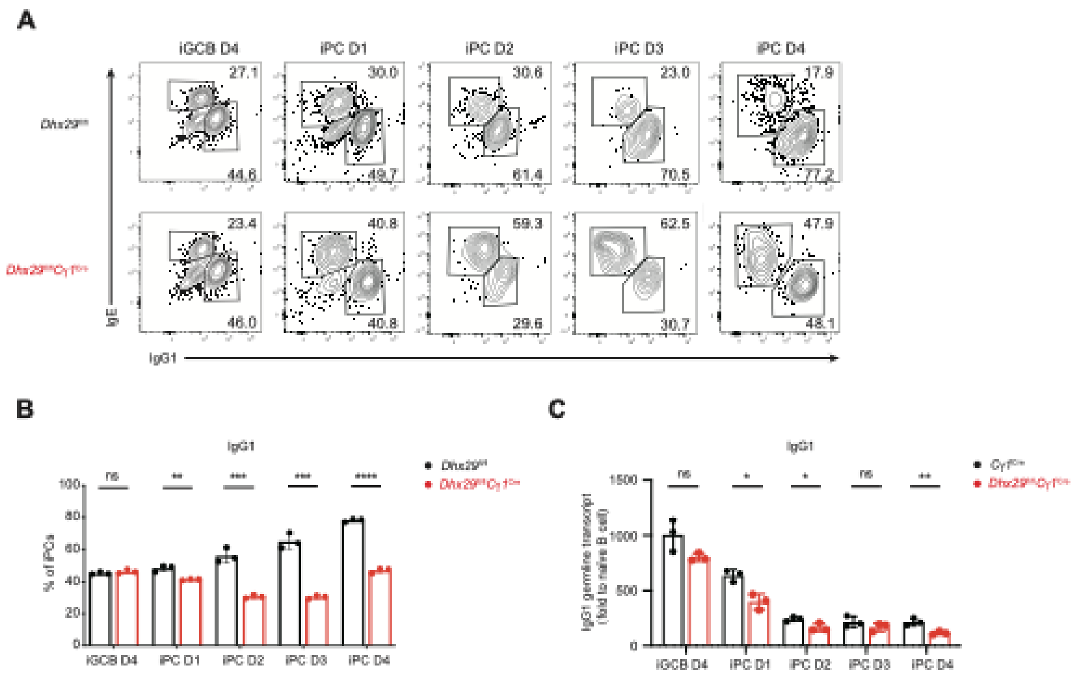

Reviewer Figure 9. Germline transcription of and class switch to IgG1 in iGCB culture.
(A-B) Flow cytometry analysis of IgG1^{+} and IgE^{+} cells among *Dhx29*$^{fl/fl}$ and *Dhx29*$^{fl/fl}$*Cγ1*^{Cre} B cells in iGCB culture. Bar graph summarizing the percentage of IgG1^{+} cells. Data from three independent biological replicates (IgG1^{+} of iPCs (%): iPC D1 P= 0.0040, iPC D2 P= 0.0007, iPC D3 P= 0.0003, iPC D4 P= 9.46E-06).
(C) Quantitative RT-PCR analysis of germline IgG1 transcript in *Cγ1*^{Cre} and *Dhx29*$^{fl/fl}$*Cγ1*^{Cre} B cells at indicated time points of iGCB culture. Data from three independent biological replicates (iPC D1: P= 0.0101, iPC D2: P= 0.0328, iPC D4: P= 0.0095). Small horizontal lines indicate the mean (± SD.). Statistical significance was determined by unpaired, two-tailed student's t-test in (B, C). $^{*}P < 0.05$, $^{**}P < 0.01$, $^{***}P < 0.001$, $^{****}P < 0.0001$.

3. Figure 6. The data is figure 6 are not convincing. The CUT&TAG product approach for TLE3 in panel A should be sequenced and a more non-biased view of TLE3 chromatin association in both Prdm1 and more globally. Moreover, the changes in luciferase expression shown are very minor. I

suggest that the data in figure 6 does not offer much insight beyond the change in Blimp1 expression shown in figure 5H after TLE3 overexpression. I suggest either improving the analysis as outlined above or perhaps removing figure 6 from the manuscript.

Response: We appreciate the reviewer's critical suggestion. We have now re-sequenced the CUT&TAG libraries and presented an unbiased genome-wide map of Tle3 occupancy, including its binding to the *Prdm1* promoter region. We also performed luciferase reporter assays and qPCR in iGCB day 5 cells after transduction with retroviruses encoding distinct sgRNAs. Those results are now included in Figure 7 of the revised manuscript.

Minor comments

4. Figure1B. Does the data point shown in the dot plot represent an individual sgRNA or the mean of 3 independent guides. This is not clear in the legend.

Response: Each dot represents an individual sgRNA. Although three gRNAs were designed for each RNA helicase, some of them did not cause significant differentiation or expansion deviation and were not included in the plot in Figure 1B. Consequently, only sgRNAs causing significant differentiation or expansion deviation were shown. This has now been clarified in the figure legend of revised manuscript.

5. The authors should be more careful with the conclusions. On page 8 they conclude that "Dh29 is required for GC response, PC differentiation etc" and the section heading suggests that "Dhx29 is indispensable for PC differentiation and CSR to IgG1". I feel that Dhx29 being required for "optimal" differentiation is a more accurate description of the data presented, as some KO cells are clearly present at each stage.

Response: We appreciate the referee's critical suggestion. We have toned down the conclusions of our study throughout the revised manuscript.

Referee #3:

The authors have carried out a study that reveals an important role of the Dhx29 helicase in promoting the translation of mRNAs that control germinal center response and plasma cell differentiation. Using a CRISPR/Cas9 screen the authors identify Dhx29 as a regulator of the immune system. Interestingly, the authors identify TCF3 and Tle3 as mRNAs needing Dhx29 for their efficient translation. Consistently, expression of TCF3 and Tle3 largely overcomes the deletion of Dhx29 in restoring plasma cell differentiation.

Overall, this study provides important information about a novel pathway that Dhx29 regulates in vivo. The experiments are rigorously carried out and the overall model is convincing. The main limitation of the study, however, is that the authors propose a mechanism by which Dhx29 promotes the translation of TCF3 and Tle3 by binding directly to their 5'UTRs (this is even stated in the abstract). I find that the data to support this direct binding model is weak and unless additional data are provided this mechanism should be toned down. Specifically, the immunoprecipitation experiment identifies reduced binding of TCF3 and Tle3 to Dhx29, but this experiment does not in fact determine if the interaction is direct or indirect. It is entirely possible that the interaction of Dhx29 with TCF3 and Tle3 is occurring indirectly through the 40S subunit, which Dhx29 binds directly to. Additional experiments would therefore need to be carried out to directly test if Dhx29 specifically binds to the 5' UTRs of these mRNAs if the authors want to propose this model. Otherwise, the data provide good information about the functional significance of Dhx29 promoting the translation of these mRNAs, but do not provide a mechanism that supports this model.

Response: We are grateful for the reviewer's appreciation of our work and appreciate this insightful suggestion. We have now performed RIP assay and biotin-labelled RNA pull-down assay, and showed that Dhx29 bound to 5' UTR of TCF3 and Tle3 mRNAs, while Dhx29-RBDmut, which lacks the dsRNA-binding domain, exhibited much reduced binding. In addition, we have also performed electrophoretic mobility shift assay (EMSA), which showed that recombinant wild type Dhx29 protein directly interacted with both 5' UTRs and that those interactions were attenuated after deletion of Dhx29 dsRNA-binding domain. Those results are now included in Figure 8D-F of the revised manuscript.

REFERENCES

Casola S, Cattoretti G, Uyttersprot N, Koralov SB, Seagal J, Hao Z, Waisman A, Egert A, Ghitza D, Rajewsky K (2006) Tracking germinal center B cells expressing germ-line immunoglobulin gamma1 transcripts by conditional gene targeting. *Proc Natl Acad Sci U S A* 103: 7396-7401

Gloury R, Zotos D, Zuidscherwoude M, Masson F, Liao Y, Hasbold J, Corcoran LM, Hodgkin PD, Belz GT, Shi W *et al* (2016) Dynamic changes in Id3 and E-protein activity orchestrate germinal center and plasma cell development. *J Exp Med* 213: 1095-1111

Kallies A, Hasbold J, Fairfax K, Pridans C, Emslie D, McKenzie BS, Lew AM, Corcoran LM, Hodgkin PD, Tarlinton DM *et al* (2007) Initiation of plasma-cell differentiation is independent of the transcription factor Blimp-1. *Immunity* 26: 555-566

Wohner M, Tagoh H, Bilic I, Jaritz M, Poliakova DK, Fischer M, Busslinger M (2016) Molecular functions of the transcription factors E2A and E2-2 in controlling germinal center B cell and plasma cell development. *J Exp Med* 213: 1201-1221

Wu J, Yang K, Cai S, Zhang X, Hu L, Lin F, Wu SQ, Xiao C, Liu WH, Han J (2022) A p38alpha-BLIMP1 signalling pathway is essential for plasma cell differentiation. *Nat Commun* 13: 7321

Dear Changchun,

Thank you again for submitting your revised manuscript (EMBOJ-2025-121591R) to The EMBO Journal for our consideration, and for your patience during re-review. Your revised manuscript has now been seen by the three original referees who had previously assessed the first version of your manuscript, and we have received their comments, which you can find appended below.

As you will see, the referees are supportive and recognize that the revised manuscript is substantially improved and the majority of the initially raised concerns have been successfully addressed. The referees point out two remaining limitations, namely the lack of phenotype rescue in vivo (mentioned by referee #1), and the insufficient demonstration of a direct interaction between DHX29 and the identified mRNAs (referee #3).

We have discussed these two points further with all three referees, and we all agree that the lack of phenotype rescue in vivo -a challenging experiment- should not preclude further consideration of the manuscript here and will not be required for publication in The EMBO Journal. On the other hand, all three referees and our team are in agreement that the mechanistic concern of referee #3 is highly relevant as it relates to a central mechanistic claim of the study and must be fully and conclusively addressed before we can accept the manuscript for publication here.

Although at The EMBO Journal we normally allow only a single round of major experimental revision, the supportive comments of all three referees and your substantial efforts to improve the manuscript during revision warrant an exception to this policy. I would thus be happy to invite you to submit a final version of the manuscript fully addressing this remaining concern, along the lines suggested by referee #3. Please include in your resubmission a point-by-point response describing and explaining any changes and additions to the manuscript. Please let me know if you have any questions or comments you would like to discuss further with me.

From the editorial side, there are also a few changes and corrections we kindly request you make in the final version of the manuscript, before we can proceed with its handling further:

- We noticed that your manuscript has an unusually high number of "co-first" authors (6 authors). Please correct this if it is a mistake, or -if it was intentional- we kindly suggest that you carefully review the actual contributions of each co-author (which will have to be specified for each co-author of the manuscript using the CRediT system - please see our author guidelines for more information: https://link.springer.com/partners/embo-press/editorial-policies#Authorship) and reduce the number of co-first authors as appropriate to more accurately reflect their actual contributions to the study and the manuscript.

- Thank you for depositing your datasets in appropriate public repositories and for providing their details in the Data availability section of your manuscript. We would be grateful if you could please add to this section the full URL for the mass spectrometry dataset deposited to the ProteomeXchange Consortium.

- Please note that if the final revision of your manuscript is accepted for publication in The EMBO Journal, all deposited datasets will have to be publicly available at the time of publication, and that we will then remove the reviewer passwords from the manuscript, as they will no longer be relevant.

- Please rename heading "Competing interests" to "Disclosure and competing interests statement".

- Please remove statement "Correspondence and requests for materials should be addressed to W.-H.L. or C.X.", as this information is redundant with the co-corresponding information already provided on the title page of the manuscript.

- All funding information provided in the Acknowledgements section of the manuscript should also be entered in our manuscript tracking system during your next submission. Currently, information regarding "National Natural Science Foundation of China (31770950 and 32070877)" is missing from the online system.

- We noticed that callouts for Appendix Figures S5 and S6 are missing from the manuscript. Please make sure that all Figures (and their panels) are called out as appropriate.

- Please rename "Table EV2" to "Dataset EV1" (its legend should be provided in a separate tab of the same Excel file), and update all callouts of EV Tables and EV Datasets accordingly throughout the manuscript.

- Please note that EMBO press papers are accompanied online by:
A) a short (2 sentences) summary of the findings and their significance,
B) 2-5 short bullet points highlighting the key results, and
C) a synopsis image in .jpg or .png format that is exactly 550 pixels wide and 300-600 pixels high (the height is variable). Please note that all text needs to be legible at the final size.

Please upload this information along with your revised manuscript (the text for A and B should be provided in a separate Word file).

- During our routine data checks, our data editors have raised the following queries regarding Figures, data, and legends. Please make sure that the following requests are fully addressed in the next version of your manuscript (all changes in the Figure legends should be highlighted in the revised manuscript):
- Please provide the exact p-values in the legends of Figures 6D, I; 7D; 8C, D.
- Please indicate the statistical test used for data analysis in the legends of Figures 5A, B.
- Please note that information related to "n" is missing in the legends of Figures 2C, E, G; 3A-C; 5A; 6B, D, H.

We look forward to seeing a final version of your manuscript as soon as possible. Please let us know if you have any questions and use this link to submit your revision: https://emboj.msubmit.net/cgi-bin/main.plex.

Best regards,

Ioannis

Ioannis Papaioannou, PhD
Editor, The EMBO Journal
i.papaioannou@embojournal.org

Referee #1:

In this revised version of the manuscript entitled "Translational control of germinal center response and plasma cell differentiation by RNA helicase Dhx29", authors successfully addressed major criticisms, reinforced key findings on the need of Dhx29 on triggering GC responses and plasma cell differentiation, and provided further mechanistic insight linking Dhx29-mediated regulation of Tcf3 and Tle3 mRNA translation and the expression of BLIMP1. In general, manuscript is much improved with experimental evidences sustaining key findings. The lack of phenotype rescue in-vivo, despite being attempted, should not be detrimental for publication, and does not diminish the relevance of the study. In this revised version, no major or minor concerns can be highlighted.

Referee #2:

The authors have done a thorough job addressing the concerns of myself and the other 2 reviewers. I have no further concerns.

Overall this is a strong and innovative study that sheds light on an understudied process in immune cells. I am in favor of publication of this manuscript as is.

Referee #3:

I appreciate the authors' efforts to address my original comment asking to demonstrate a direct interaction between DHX29 and the identified mRNAs by performing EMSA experiments. While encouraging, the current gel shift data do not yet provide convincing evidence for a specific, direct interaction between the identified protein and the 5′ UTRs of these mRNAs. The native gels show at least two shifted species, including a slowly migrating signal that appears consistent with material retained near the well, and the identity of these bands is not defined. In addition, the assays appear to be performed at very high RNA and protein concentrations (concentrations are not reported, only total amounts), and they lack appropriate specificity controls, making it difficult to distinguish bona fide binding from nonspecific or aggregated complexes.

To support the proposed model, additional experiments are required, including protein titration to demonstrate dose-dependent and saturable binding, as well as specificity controls such as unrelated control RNAs and competition with excess unlabeled cognate versus nonspecific RNA. These experiments would help define the shifted species and establish whether the observed interactions reflect specific, direct binding to the 5′ UTRs.

We thank the reviewers for their encouraging and constructive comments. In response to the concerns raised by Reviewer #3, we have performed additional experiments and revised the manuscript accordingly. We have also addressed the editorial issues related to formatting and data annotation. Below, we provide a point-by-point response to the reviewers' comments and hope that the revised manuscript is now suitable for publication.

Referee #1:
In this revised version of the manuscript entitled "Translational control of germinal center response and plasma cell differentiation by RNA helicase Dhx29", authors successfully addressed major criticisms, reinforced key findings on the need of Dhx29 on triggering GC responses and plasma cell differentiation, and provided further mechanistic insight linking Dhx29-mediated regulation of Tcf3 and Tle3 mRNA translation and the expression of BLIMP1. In general, manuscript is much improved with experimental evidences sustaining key findings. The lack of phenotype rescue in-vivo, despite being attempted, should not be detrimental for publication, and does not diminish the relevance of the study. In this revised version, no major or minor concerns can be highlighted.

Referee #2:
The authors have done a thorough job addressing the concerns of myself and the other 2 reviewers. I have no further concerns.

Overall this is a strong and innovative study that sheds light on an understudied process in immune cells. I am in favor of publication of this manuscript as is.

Referee #3:
I appreciate the authors' efforts to address my original comment asking to demonstrate a direct interaction between DHX29 and the identified mRNAs by performing EMSA experiments. While encouraging, the current gel shift data do not yet provide convincing evidence for a specific, direct interaction between the identified protein and the 5' UTRs of these mRNAs. The native gels show at least two shifted species, including a slowly migrating signal that appears consistent with material retained near the well, and the identity of these bands is not defined. In addition, the assays appear to be performed at very high RNA and protein concentrations (concentrations are not reported, only total amounts), and they lack appropriate specificity controls, making it difficult to distinguish bona fide binding from nonspecific or aggregated complexes.

To support the proposed model, additional experiments are required, including protein titration to demonstrate dose-dependent and saturable binding, as well as specificity controls such as unrelated control RNAs and competition with excess unlabeled cognate versus nonspecific RNA. These experiments would help define the shifted species and establish whether the observed interactions reflect specific, direct binding to the 5' UTRs.

Response: As suggested by the reviewer, we performed additional EMSA experiments to further substantiate that Dhx29 binds directly and specifically to the 5'UTRs of *Tle3* and *Tcf3*. As shown in Reviewer Figure 1, purified Dhx29 bound the 5'UTRs of *Tle3* and *Tcf3* in a dose-dependent manner (A), and this interaction was efficiently competed by excess unlabeled cognate 5'UTR RNA (B). In addition, Dhx29 bound the sense strands of these 5'UTRs, but not the corresponding antisense strands (C). Together, these results support specific and direct binding of Dhx29 to the 5'UTRs

of *Tle3* and *Tcf3*. These new data have now been incorporated into Figure EV5 of the revised manuscript.

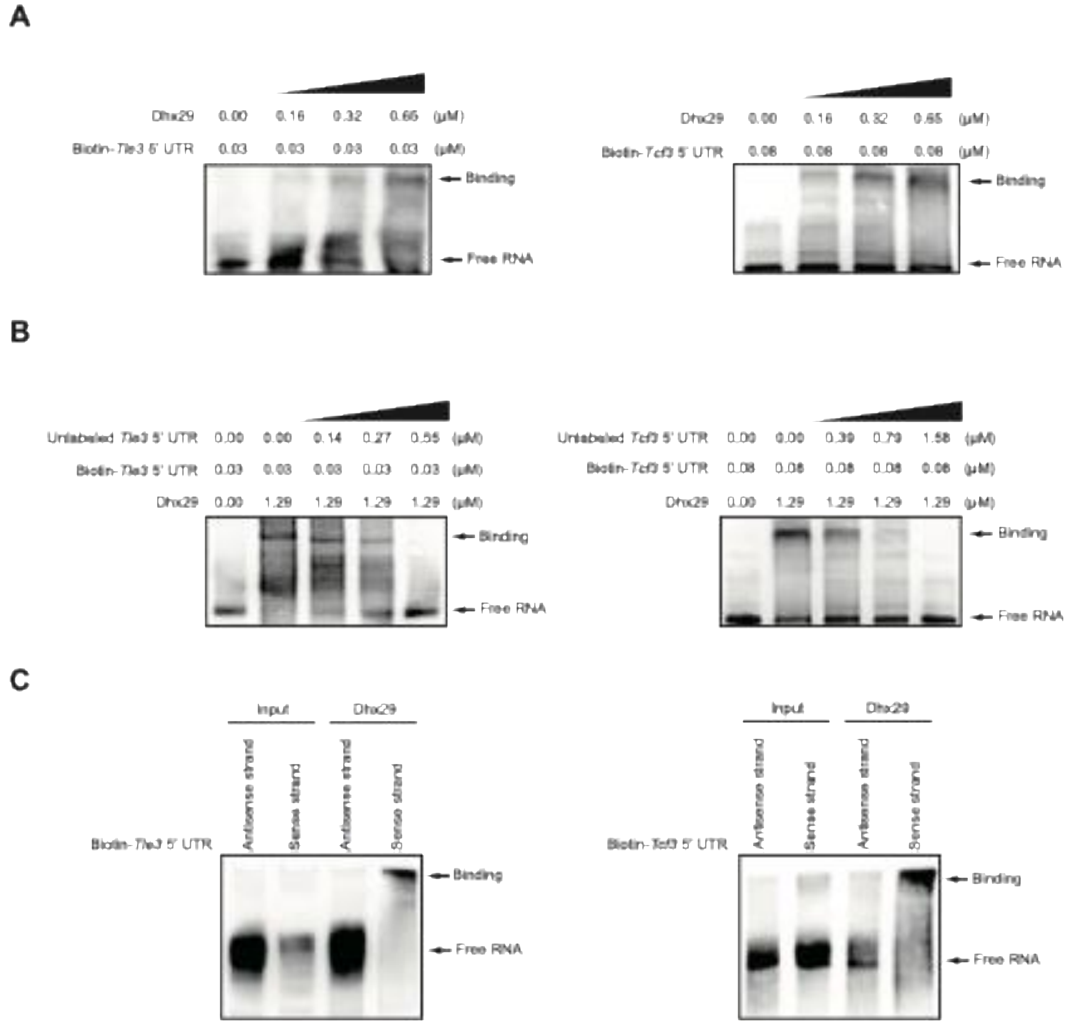

Reviewer Figure 1. Dhx29 binds directly and specifically to the 5'UTRs of *Tle3* and *Tcf3*

(A) Dhx29 binds the 5'UTRs of *Tle3* and *Tcf3* in a dose-dependent manner.

(B) Dhx29 interactions with the 5'UTRs of *Tle3* and *Tcf3* were competed by excess unlabeled cognate 5'UTR RNA.

(C) Dhx29 binds the sense strands of these 5'UTRs, but not the corresponding antisense strands. *Tle3* 5'UTR sense and antisense strands, 0.03 μM; *Tcf3* 5'UTR sense and antisense strands, 0.08 μM; Dhx29 protein, 1.29 μM.

2nd Revision - Editorial Decision 24th Apr 2026

Dear Changchun,

Thank you for the submission of your new revision (EMBOJ-2025-121591R1) to The EMBO Journal for our consideration, and for your patience during re-review. The new version of your manuscript has now been seen by referee #3, and we have received their comments, which you can find appended below.

I am very pleased to say that, as you will see, the referee is satisfied with the revision and recognizes that the previously raised concern has now been adequately addressed. I am therefore happy to inform you that your manuscript has been accepted in principle for publication in The EMBO Journal.

Before we can proceed with the next steps towards online publication of the article, there are a few remaining minor issues that we kindly ask you to address:

- Please note that all funding information provided in the Acknowledgements section of the manuscript should also be entered in our online manuscript tracking system. Currently, information regarding support by the "Sanofi Institute for Biomedical Research (SIBR)" is missing in the online system.

- Callouts "Appendix Figure 5" and "Appendix Figure 6" must be corrected to "Appendix Figure S5" and "Appendix Figure S6", respectively, throughout the manuscript.

- Please check once again that the contents and callouts of "Table EV1", "Table EV2", and "Dataset EV1" are correct, as intended.

- Please also note that as part of the EMBO Press transparent editorial process, The EMBO Journal publishes online a Peer Review File along with each accepted manuscript. This File will be published in conjunction with your paper and will include the referee reports, your point-by-point responses and all pertinent correspondence relating to the manuscript. Your Author's Checklist will also be published at the end of the Peer Review File. Please let us know in case you want to remove any data or figures from your point-by-point responses before they are published as part of the Peer Review File. Retaining unpublished data in the Peer Review File means that these count as published and that the Peer Review File would need to be referenced in future publications. Please let the editorial office know in case you want to remove any data from this file (contact@embojournal.org).

We look forward to seeing a final version of your manuscript as soon as possible. Please let us know if you have any questions and use this link to submit your revision: https://emboj.msubmit.net/cgi-bin/main.plex.

Best regards,

Ioannis

Ioannis Papaioannou, PhD
Editor, The EMBO Journal
i.papaioannou@embojournal.org

Referee #3:

I am satisfied that the authors have suitably addressed my main comments regarding the need for improved gel shifts.

All editorial and formatting issues were resolved by the authors.

3rd Revision - Editorial Decision 27th Apr 2026

Dear Changchun,

Congratulations on an excellent work! I am very pleased to inform you that your manuscript has been accepted for publication in The EMBO Journal. Thank you for comprehensively addressing the initially raised referee concerns and our editorial requests for corrections and changes.

Your manuscript will be processed for publication by EMBO Press. It will be copy edited and you will receive page proofs prior to publication. Please note that you will be contacted by Springer Nature Author Services to complete licensing and payment information.

You may qualify for financial assistance for your publication charges - either via a Springer Nature fully open access agreement or an EMBO initiative. Check your eligibility: https://link.springer.com/journal/44318/how-to-publish-with-us

Should you be planning a Press Release on your article, please get in contact with embo_production@springernature.com as early as possible in order to coordinate publication and release dates.

If you have any questions, please do not hesitate to contact the Editorial Office. Thank you for your contribution to The EMBO Journal. Working with you has been a pleasure.

Best regards,

Ioannis

Ioannis Papaioannou, PhD
Editor, The EMBO Journal
i.papaioannou@embojournal.org

*****
Please note that it is The EMBO Journal policy for the transcript of the editorial process (containing referee reports and your response letters) to be published as an online supplement to each paper. If you should prefer removal of any referee-only figures included in the point-by-point response(s), e.g. because they may still be used for future publication or because they have been reproduced from published work by others, please do let us know immediately via response email.
More information is available here: https://link.springer.com/partners/embo-press/editorial-policies#Peer%20review

** Click here to be directed to your login page: https://emboj.msubmit.net

EMBO Press Author Checklist

Corresponding Author Name: Changchun Xiao
Journal Submitted to: The EMBO Journal
Manuscript Number: EMBOJ-2025-121591

USEFUL LINKS FOR COMPLETING THIS FORM
The EMBO Journal - Author Guidelines
EMBO Reports - Author Guidelines
Molecular Systems Biology - Author Guidelines
EMBO Molecular Medicine - Author Guidelines

Reporting Checklist for Life Science Articles (updated January
This checklist is adapted from Materials Design Analysis Reporting (MDAR) Checklist for Authors. MDAR establishes a minimum set of requirements in transparent reporting in the life sciences (see Statement of Task: 10.31222/osf.io/9sm4x). Please follow the journal's guidelines in preparing your
Please note that a copy of this checklist will be published alongside your article.

Abridged guidelines for figures
1. Data
The data shown in figures should satisfy the following conditions:
- the data were obtained and processed according to the field's best practice and are presented to reflect the results of the experiments in an accurate and unbiased manner.
- ideally, figure panels should include only measurements that are directly comparable to each other and obtained with the same assay.
- plots include clearly labeled error bars for independent experiments and sample sizes. Unless justified, error bars should not be shown for technical
- if n<5, the individual data points from each experiment should be plotted. Any statistical test employed should be justified.
- Source Data should be included to report the data underlying figures according to the guidelines set out in the authorship guidelines on Data

2. Captions
Each figure caption should contain the following information, for each panel where they are relevant:
- a specification of the experimental system investigated (eg cell line, species name).
- the assay(s) and method(s) used to carry out the reported observations and measurements.
- an explicit mention of the biological and chemical entity(ies) that are being measured.
- an explicit mention of the biological and chemical entity(ies) that are altered/varied/perturbed in a controlled manner.
- the exact sample size (n) for each experimental group/condition, given as a number, not a range;
- a description of the sample collection allowing the reader to understand whether the samples represent technical or biological replicates (including how many animals, litters, cultures, etc.).
- a statement of how many times the experiment shown was independently replicated in the laboratory.
- definitions of statistical methods and measures:
  - common tests, such as t-test (please specify whether paired vs. unpaired), simple χ2 tests, Wilcoxon and Mann-Whitney tests, can be unambiguously identified by name only, but more complex techniques should be described in the methods section;
  - are tests one-sided or two-sided?
  - are there adjustments for multiple comparisons?
  - exact statistical test results, e.g., P values = x but not P values < x;
  - definition of 'center values' as median or average;
  - definition of error bars as s.d. or s.e.m.

Please complete ALL of the questions below.
Select "Not Applicable" only when the requested information is not relevant for your study.

Materials

Newly Created Materials	Information included in the manuscript?	In which section is the information available? (Reagents and Tools Table, Materials and Methods, Figures, Data Availability Section)
New materials and reagents need to be available; do any restrictions apply?	Not Applicable	

Antibodies	Information included in the manuscript?	In which section is the information available? (Reagents and Tools Table, Materials and Methods, Figures, Data Availability Section)
For **antibodies** provide the following information:<br>- Commercial antibodies: RRID (if possible) or supplier name, catalogue number and or/clone number<br>- Non-commercial: RRID or citation	Yes	Reagents and Tools Table

DNA and RNA sequences	Information included in the manuscript?	In which section is the information available? (Reagents and Tools Table, Materials and Methods, Figures, Data Availability Section)
**Short novel DNA or RNA including primers, probes**: provide the sequences.	Yes	Table EV2 and Dataset EV1

Cell materials	Information included in the manuscript?	In which section is the information available? (Reagents and Tools Table, Materials and Methods, Figures, Data Availability Section)
**Cell lines:** Provide species information, strain. Provide accession number in repository **OR** supplier name, catalog number, clone number, and/**OR** RRID.	Yes	Reagents and Tools Table
**Primary cultures:** Provide species, strain, sex of origin, genetic modification status.	Yes	Reagents and Tools Table，Methods
Report if the cell lines were recently **authenticated** (e.g., by STR profiling) and tested for mycoplasma contamination.	Yes	Methods

Experimental animals	Information included in the manuscript?	In which section is the information available? (Reagents and Tools Table, Materials and Methods, Figures, Data Availability Section)
**Laboratory animals or Model organisms:** Provide species, strain, sex, age, genetic modification status. Provide accession number in repository **OR** supplier name, catalog number, clone number, **OR** RRID.	Yes	Reagents and Tools Table，Methods
**Animal observed in or captured from the field:** Provide species, sex, and age where possible.	Not Applicable	
Please detail **housing and husbandry conditions**.	Yes	Methods

Plants and microbes	Information included in the manuscript?	In which section is the information available? (Reagents and Tools Table, Materials and Methods, Figures, Data Availability Section)
**Plants:** provide species and strain, ecotype and cultivar where relevant, unique accession number if available, and source (including location for collected wild specimens).	Not Applicable	
**Microbes:** provide species and strain, unique accession number if available, and source.	Yes	Methods

Human research participants	Information included in the manuscript?	In which section is the information available? (Reagents and Tools Table, Materials and Methods, Figures, Data Availability Section)
If collected and within the bounds of privacy constraints report on age, sex and gender or ethnicity for all study participants.	Not Applicable	

Core facilities	Information included in the manuscript?	In which section is the information available? (Reagents and Tools Table, Materials and Methods, Figures, Data Availability Section)
If your work benefited from core facilities, was their service mentioned in the acknowledgments section?	Yes	Acknowledgements

Design

Study protocol	Information included in the manuscript?	In which section is the information available? (Reagents and Tools Table, Materials and Methods, Figures, Data Availability Section)
If study protocol has been **pre-registered, provide DOI in the manuscript**. For clinical trials, provide the trial registration number **OR** cite DOI.	Not Applicable	
Report the **clinical trial registration number** (at ClinicalTrials.gov or equivalent), where applicable.	Not Applicable	

Laboratory protocol	Information included in the manuscript?	In which section is the information available? (Reagents and Tools Table, Materials and Methods, Figures, Data Availability Section)
Provide DOI OR other citation details if **external detailed step-by-step protocols** are available.	Not Applicable	

Experimental study design and statistics	Information included in the manuscript?	In which section is the information available? (Reagents and Tools Table, Materials and Methods, Figures, Data Availability Section)
Include a statement about **sample size** estimate even if no statistical methods were used.	Yes	Figures
Were any steps taken to minimize the effects of subjective bias when allocating animals/samples to treatment (e.g. **randomization procedure**)? If yes, have they been described?	Not Applicable	
Include a statement about **blinding** even if no blinding was done.	Not Applicable	
Describe **inclusion/exclusion criteria** if samples or animals were excluded from the analysis. Were the criteria pre-established?<br><br>If sample or data points were omitted from analysis, report if this was due to attrition or intentional exclusion and provide justification.	Not Applicable	
For every figure, are **statistical tests** justified as appropriate? Do the data meet the assumptions of the tests (e.g., normal distribution)? Describe any methods used to assess it. Is there an estimate of variation within each group of data? Is the variance similar between the groups that are being statistically compared?	Yes	Figure legends

Sample definition and in-laboratory replication	Information included in the manuscript?	In which section is the information available? (Reagents and Tools Table, Materials and Methods, Figures, Data Availability Section)
In the figure legends: state number of times the experiment was **replicated** in laboratory.	Yes	Figure legends
In the figure legends: define whether data describe **technical or biological replicates**.	Yes	Figure legends

Ethics

Ethics	Information included in the manuscript?	In which section is the information available? (Reagents and Tools Table, Materials and Methods, Figures, Data Availability Section)
Studies involving **human participants**: State details of **authority granting ethics approval** (IRB or equivalent committee(s), provide reference number for approval.	Not Applicable	
Studies involving **human participants**: Include a statement confirming that **informed consent** was obtained from all subjects and that the experiments conformed to the principles set out in the WMA Declaration of Helsinki and the Department of Health and Human Services Belmont Report.	Not Applicable	
Studies involving **human participants:** For publication of **patient photos**, include a statement confirming that consent to publish was obtained.	Not Applicable	
Studies involving experimental **animals**: State details of **authority granting ethics approval** (IRB or equivalent committee(s), provide reference number for approval. Include a statement of compliance with ethical regulations.	Yes	Methods
Studies involving **specimen and field samples:** State if relevant **permits** obtained, provide details of authority approving study; if none were required, explain why.	Not Applicable	

Dual Use Research of Concern (DURC)	Information included in the manuscript?	In which section is the information available? (Reagents and Tools Table, Materials and Methods, Figures, Data Availability Section)
Could your study fall under dual use research restrictions? Please check biosecurity documents and list of **select agents and toxins** (CDC): https://www.selectagents.gov/sat/list.htm	Not Applicable	
If you used a select agent, is the security level of the lab appropriate and reported in the manuscript?	Not Applicable	
If a study is subject to dual use research of concern regulations, is the name of the **authority granting approval and reference number** for the regulatory approval provided in the manuscript?	Not Applicable	

Reporting

The MDAR framework recommends adoption of discipline-specific guidelines, established and endorsed through community initiatives. Journals have their own policy about requiring specific guidelines and recommendations to complement MDAR.

Adherence to community standards	Information included in the manuscript?	In which section is the information available? (Reagents and Tools Table, Materials and Methods, Figures, Data Availability Section)
State if relevant guidelines or checklists (e.g., **ICMJE, MIBBI, ARRIVE, PRISMA**) have been followed or provided.	Not Applicable	
For **tumor marker prognostic studies,** we recommend that you follow the **REMARK** reporting guidelines (see link list at top right). See author guidelines, under 'Reporting Guidelines'. Please confirm you have followed these guidelines.	Not Applicable	
For **phase II and III randomized controlled trials**, please refer to the **CONSORT** flow diagram (see link list at top right) and submit the CONSORT checklist (see link list at top right) with your submission. See author guidelines, under 'Reporting Guidelines'. Please confirm you have submitted this list.	Not Applicable	

Data Availability

Data availability	Information included in the manuscript?	In which section is the information available? (Reagents and Tools Table, Materials and Methods, Figures, Data Availability Section)
Have **primary datasets** been deposited according to the journal's guidelines (see 'Data Deposition' section) and the respective accession numbers provided in the Data Availability Section?	Yes	Data availability section
Were **human clinical and genomic datasets** deposited in a public access-controlled repository in accordance to ethical obligations to the patients and to the applicable consent agreement?	Not Applicable	
Are **computational models** that are central and integral to a study available without restrictions in a machine-readable form? Were the relevant accession numbers or links provided?	Not Applicable	
If publicly available data were reused, provide the respective **data citations in the reference list**.	Not Applicable	
